# Protocol update for a multi-centre randomised controlled trial of exercise rehabilitation for people with pulmonary hypertension: the SPHERe trial

**DOI:** 10.1186/s13063-024-08341-0

**Published:** 2024-07-20

**Authors:** Stuart Ennis, Julie Bruce, Harbinder Sandhu, Mariam Ratna, Ranjit Lall, Chen Ji, James Mason, Rebecca Kandiyali, Kate Seers, Prithwish Banerjee, Stephanie J. C. Taylor, Elizabeth Robertson, Martin Underwood, Gordon McGregor

**Affiliations:** 1 Warwick Medical School, University of Warwick, Coventry, UK; 2 Research & Development, University Hospitals Coventry & Warwickshire NHS Trust, Coventry, UK; 3 Institute of Population Health Sciences, Barts and The London School of Medicine and Dentistry, Queen Mary University of London, London, UK; 4 Patient & Public Involvement Representative, London, UK; 5 Centre for Healthcare & Communities, Coventry University, Coventry, UK

**Keywords:** Pulmonary hypertension, Cardiopulmonary rehabilitation, Complex intervention, Online exercise

## Abstract

**Background:**

The SPHERe (Supervised Pulmonary Hypertension Exercise Rehabilitation) trial is a multi-centre, pragmatic, randomised controlled trial assessing the clinical and cost-effectiveness of supervised exercise rehabilitation with psychosocial and motivational support compared to best-practice usual care for people with pulmonary hypertension (PH). The original protocol was published in BMC Pulmonary Medicine (accessible online). We randomised our first participant in January 2020. In response to the COVID-19 pandemic, the trial was stopped in March 2020. In person delivery of the SPHERe intervention to a vulnerable population was not possible during the COVID-19 pandemic. We describe here how trial procedures and intervention delivery were adapted in response to the COVID-19 pandemic.

**Methods:**

Restrictions imposed by the COVID-19 pandemic on the clinically vulnerable PH population meant that trial delivery was changed from a centre-based rehabilitation programme to remotely delivered group online sessions. This led to minor alterations to the eligibility criteria. These changes followed a consultation process with stakeholders and people with PH and were approved by the funder and independent trial committees.

**Conclusions:**

We describe the modified SPHERe trial protocol in response to restrictions imposed by the COVID-19 pandemic. SPHERe is the first randomised controlled trial to assess the clinical and cost-effectiveness of an online group rehabilitation programme for people with PH compared to usual care.

**Trial registration:**

ISRCTN no. 10608766. Prospectively registered on 18th March 2019, updated 16th August 2023.

**Supplementary Information:**

The online version contains supplementary material available at 10.1186/s13063-024-08341-0.

## Background

The SPHERe (Supervised Pulmonary Hypertension Exercise Rehabilitation) trial is a multi-centre, pragmatic, randomised controlled trial assessing the clinical and cost-effectiveness of supervised exercise rehabilitation with psychosocial and motivational support compared to best-practice usual care for people with pulmonary hypertension (PH) [1].

In March 2020, trial delivery was changed from a centre-based rehabilitation programme to remotely delivered group online sessions due to the impact of the COVID-19 pandemic on the clinically vulnerable PH population. Accordingly, following a consultation process with stakeholders and people with PH, and funder and independent trial committees approval, minor alterations to eligibility criteria were required along with adaptations to outcomes assessment and intervention delivery procedures.

The SPHERe trial will assess the clinical and cost-effectiveness of a remotely supervised online rehabilitation programme for people with PH compared to best-practice usual care. Here we report enforced adaptions to the trial protocol and intervention delivery in the context of the COVID-19 pandemic.

## Methods/design

### On‑line intervention redesign meetings

The research team, stakeholders and patient partners attended several online meetings to adapt the intervention for safe delivery in the context of the COVID-19 pandemic and associated restrictions on movement. People with PH and other trial team members (specialising in psychology, exercise physiology, physiotherapy, methodology), attended the meetings. We emailed the suggested adaptations of the SPHERe and usual care interventions to all attendees before each meeting. Suggested changes were explained to the group and discussed from the perspectives of all representatives. It was an iterative process, using multiple communication strategies, to ensure the inclusion of people who were affected differently by PH, e.g. IT literacy, symptoms and exercise history. Discussions were structured into themes e.g. safety, online technology, delivery, standardisation etc., which would aid the revised design of the SPHERe and usual care interventions.

Although a remotely supervised, online, home-based exercise rehabilitation programme for people with PH would be completely novel, we considered the available evidence for online/remote rehabilitation programmes in COPD [2, 3] and heart failure [4, 5]. This previous work informed the adaptation of the SPHERe intervention from centre-based to supervised online delivery. We concluded that the intervention should be structured, and resource-based (participant manual, live and prerecorded online content), using home-based functional exercise (bodyweight or chair-based) and equipment (upright/ recumbent exercise bike), facilitated by trained practitioners, with remotely supervised live sessions [6, 7].

### Overview of trial adaptations

It was agreed that all SPHERe exercise and psychosocial and motivational support sessions would be delivered remotely in groups online using a bespoke platform (www.beamfeelgood.com) and facilitated by practitioners at a central trial hub (Atrium Health Ltd, Centre for Exercise and Health, Coventry) under sub-contract to University Hospitals Coventry and Warwickshire NHS Trust. The revised protocol was approved by the funder and independent trial committees. Accordingly, the eligibility criteria, outcome assessments, and SPHERe/control intervention components were revised. Full description of the revised exercise and psychosocial and motivational support sessions is available elsewhere [6]. The trial—ISRCTN 10608766—was prospectively registered on 18th March 2019, and updated on 16th August 2023.

### Eligibility criteria

One change was made to the inclusion criteria. Prospective participants were required to have access to appropriate IT infrastructure (Table 1). To avoid discriminating against those without the appropriate IT infrastructure, a suitable device (tablet) and guidance for use were provided as required. This change was reflected in the participant information leaflet. Participants were required to have internet access, and an email address to enrol on the online platform.

**Table 1:** Inclusion and exclusion criteria

**Inclusion criteria**	**Exclusion criteria**
▪ Adults (18 +) with confirmed PH (groups 1 to 5) as detailed in European Society of Cardiology/European Respiratory Society guidelines [7]. ▪ Clinically stable: Groups 1, 4, and 5—stable on optimal PH-specific drug therapy (for those in whom it is appropriate) for at least 1 month, or evidence that these drugs cannot be tolerated. PH Groups 2 and 3—stable on drug therapy for underlying cardiac or pulmonary disease for at least 1 month. Clinical stability defined as presenting with, reproducible, manageable symptoms, not requiring any treatment other than routine follow-up care, and no PH-related hospital admission in the last 4 weeks. ▪ World Health Organisation (WHO) functional class II, III or IV. ▪ Fluent in spoken English to allow engagement with intervention and physical outcome measures. ▪ Live within reasonable travelling distance (as defined by the participant) of a SPHERe outcomes assessment centre. ▪ Able to access appropriate IT infrastructure (internet, email, computer, laptop, tablet, smart phone) ▪ Able to provide informed consent.	▪ Absolute contraindications to exercise as per international clinical guidelines. ▪ PH-related complications, or comorbidities severe enough to prevent attendance at a SPHERe outcomes assessment centre, or participation in exercise rehabilitation. ▪ Any mental health issue that will prevent engagement with trial procedures. ▪ Previous randomisation in the present trial ▪ Pregnant at time of recruitment.

### Outcome assessments

The only direct in-person contact between participants and trial staff was during outcomes assessment appointments. The primary outcome, the incremental shuttle walk test, could not be completed remotely online. Participants needed to be able to travel to one of our previously planned intervention sites for assessment. To minimise the risk of COVID-19 transmission in this clinically vulnerable population, stringent COVID-19 risk-reducing procedures were implemented. During the initial telephone screening call participants were informed of the procedures for outcome assessment appointments, to allay any concerns they might have. All trial sites were advised that only one member of staff should have direct contact with the participant during outcomes assessment appointments, full personal protective equipment (PPE) should be worn, and regular self-testing and social distancing guidelines were strictly followed in accordance with NHS policy at the time.

With the aim of minimising the time that participants spent at outcomes assessment appointments, the 6-min walk test was removed from secondary outcomes. Additionally, all sites were instructed to send the baseline questionnaire to participants to complete prior to attendance at their initial assessment.

### The SPHERe intervention

Participants randomised to the SPHERe intervention joined online groups of up to eight participants to undertake live exercise and psychosocial and motivational support sessions, remotely facilitated by a SPHERe practitioner [8]. They also had access to an exercise bike delivered to their home, and received weekly individual catch-up calls from the practitioner to discuss and amend their home exercise plan. They were also given access to an online library of on-demand exercise demonstration videos to provide instructional support [8].

### Online individual assessment

Participants underwent a 1-hour one-to-one, online appointment with a SPHERe practitioner to record a comprehensive medical and physical activity history. Participants were also introduced to the online platform, and the logistics of the home exercise programme. Following this, the SPHERe practitioner prescribed a tailored, individualised exercise programme within pre-specified parameters, as outlined in the practitioner manual [8]. Clinical information, data from the baseline walking test, and patient-centred goal setting were used to devise a safe and effective exercise prescription. The participant was sent a copy of the programme via email.

### Supervised live online exercise sessions (weeks 1 to 8)

Participants were invited to attend eight once-weekly practitioner-led, online group exercise sessions, each session lasting up to one hour. During these sessions, practitioners closely monitored participants and suggested alternative/adapted exercises to individualise and personalise the session as much as possible. Sessions consisted of warm-up and mobility activities followed by moderate-intensity exercise (40–70% heart rate reserve; rating of perceived exertion 12–14; breathlessness scale 3–4) (16). Exercise sessions included aerobic, muscular strength and endurance, and ‘functional’ exercise [8].

### Psychosocial and motivational support and education (weeks 2 to 8)

Participants were invited to attend six 30-min, online group psychosocial and motivational support sessions with a trained SPHERe practitioner, delivered over six weeks of the 8-week programme. These online sessions were held either before or after the exercise session. The primary goal of these sessions was to promote adherence to exercise, allay fears around fatigue and breathlessness, and promote peer support. They featured pre-recorded videos containing an introduction to each session topic produced by the trial health psychologist with graphical slides to assist understanding. Videos were played online to the group to generate discussion and to promote the sharing of participants’ experiences of living with PH.

### Guided home exercise plan (weeks 1 to 8)

An individualised, unsupervised exercise plan was given to participants to follow at least twice per week at home. Most participants were provided with a stationary upright or recumbent exercise bike, delivered to their home, except in circumstances where this was not feasible (e.g. lack of space). In agreement with the practitioner, participants completed a fixed duration and intensity exercise programme at least once per week using the exercise bike. Participants were also given a ‘home exercise circuit’ involving 6–8 functional fitness exercises from a menu of 24 exercises available on-demand on the online platform. The participant was instructed to perform these selected exercises in a ‘circuit’; i.e. one after another, and multiple circuits repeated as required. The practitioner reviewed the individualised supervised and unsupervised home exercise programmes weekly with exercises adapted and progressed as required.

### Safety

Delivering online exercise to the high-risk PH population required stringent monitoring and safety procedures to minimise adverse events during or after exercise. All live exercise sessions were delivered by experienced exercise practitioners based at the central trial hub. Live group exercise sessions allowed real-time supervision and instruction. At each live exercise session, one practitioner led the exercise session while a designated ‘co-pilot’ was available to deal with any safety concerns, adverse events or IT issues that arose. Each session had a maximum of eight attendees to ensure safety could be monitored. A comprehensive safety protocol was implemented.

For the guided home exercise bike and functional fitness programme, short instructional exercise demonstration videos were available for participants to view on-demand via an online platform. This was to promote correct technique and reduce the chance of injury when participants exercised unsupervised. Intervention practitioners were specialist exercise physiologists or physiotherapists, experienced in assessment, prescription, and delivery of exercise in high-risk clinical populations. Exercise intensity was set at the lower end of therapeutic levels to minimise the chance of participants overexerting themselves when unsupervised. All participants were advised to perform their home exercise programme when someone else was at home/contactable should they experience any difficulty.

SPHERe outcome assessments were undertaken in NHS facilities with access to emergency equipment and qualified staff. Condition-specific monitoring of exercise responses, e.g. pulse oximetry, heart rate, as per cardio-pulmonary rehabilitation guidelines, were used to mitigate and manage risk [9–11].

The guided home exercise plan was low-moderate intensity and fully manualised with video demonstrations available online to minimise adverse events for participants exercising unsupervised. Training in the standardised delivery of the SPHERe interventions and trial procedures was provided for all practitioners, and bespoke manuals were produced to guide the delivery of all intervention components.

### Technological considerations

Participants were advised of the minimum IT requirements for the trial. Where necessary, the trial team spent considerable time advising and instructing participants on the use of devices/applications. Additional written instructions were also made available in the participant manual.

### Control intervention: best-practice usual care

The control intervention was described as ‘best-practice usual care’, in the form of a one-to-one online appointment, with general advice on safe and effective physical activity for those living with PH. A single online 30-min appointment allowed the practitioner to discuss individualised ways in which the participant could undertake physical activity safely at home.

### Sample size revision

The COVID-19 pandemic made the recruiting of NHS trusts as sites for SPHERe very difficult due to the lack of capacity in many NHS research and development departments and the prioritisation of COVID-19-focused trials from 2020 to 2022. With the guidance of the Data Monitoring and Ethics Committee, we revised our sample size at an appropriate time in the study. This timing was dictated by the Data Monitoring Committee when sample observed data was available. The re-estimation included the observed correlation coefficient between baseline and primary outcome follow-up ISWT. We recalculated our sample size using observed parameters from 79 randomisations and 43 participants with complete primary outcome data using the following observations:

1. Number of patients in PH groups 2/3 = 35/79 (44%)
2. Intervention group size = between 5 and 10
3. Intra-cluster coefficient (ICC) = 0.03
4. Allocation ratio = 1.04:1 (cluster size = 5) and 1.10:1 (cluster size = 10)
5. Effect size = 0.5
6. Lost to follow-up = 24%
7. Correlation coefficient = 0.8

To show our target difference, with this level of correlation, we needed to recruit 85–90 participants with type 2/3 PH to show a benefit in this key group (specified in the original National Institute for Health and Care Research Health Technology Assessment (NIHR HTA) brief). Thus, we aimed for an overall target of around 200 participants but with an intention to stop recruitment when 85–90 participants with type 2/3 PH had been enrolled.

The sample size revision was reviewed and approved by the TSC, DMC, Sponsor and Funder (NIHR HTA) in November 2022. Updates to the Statistics and Health Economics Analysis Plan (SHEAP) were made in consultation with our trial statisticians. We present the detailed SHEAP for the SPHERe trial in supplementary material, which has not previously been published,

### Trial status

Recruitment to the internal pilot began in January 2020 and was subsequently temporarily suspended in March 2020 due to the COVID-19 pandemic. The trial was then adapted during 2020 and NHS sites slowly reopened, and recruitment recommenced in May 2021. The trial received contract extensions from the funder (NIHR), finishing recruitment in August 2023. The trial is now in follow-up. The proportion of people with type 2/3 PH was smaller than anticipated at the time of trial design. This may reflect the move to online intervention delivery during the COVID-19 pandemic, meaning fewer older people with type 2/3 PH were able/willing to access the intervention (issues with access/competence in the use of technology).

## Discussion

Further to the publication of the original protocol in May 2020, the SPHERe trial protocol underwent considerable redesign in response to the COVID-19 pandemic. The SPHERe intervention and best-practice usual care are now delivered exclusively remotely online. Results from the SPHERe trial will inform many areas of clinical practice. Firstly, clinicians and rehabilitation practitioners will gain valuable insight into the specific exercise rehabilitation requirements of people with PH. Secondly, they will have definitive answers as to the clinical efficacy of NHS online exercise rehabilitation programmes for this diverse population. Thirdly, commissioners will be able to appraise the clinical and cost-effectiveness of remote online programmes and inform commissioning strategies accordingly. Finally, people with all forms of PH will be provided with a greater understanding of the potential benefit or harm of online exercise rehabilitation and will be better placed to make informed decisions as to their future participation.

### Supplementary Information

Supplementary Material 1.

## Data Availability

All data requests should be submitted to WCTUDataAccess@warwick.ac.uk for consideration. Access to anonymised data may be granted following review.

## Abbreviations

CHF: Chronic heart failure
CI: Confidence interval
COPD: Chronic obstructive pulmonary disease
DMC: Data Monitoring Committee
HTA: Health technology assessment
ICC: Intra-class correlation co-efficient
ISRCTN: International Standard Randomised Controlled Trial Number
ISWT: Incremental shuttle walk test
NICE: National Institute for Health and Care Excellence
NIHR: National Institute for Health and Care Research
MRC: Medical Research Council
PH: Pulmonary hypertension
PPE: Personal protective equipment
HRQoL: Health-related quality of life
RCT: Randomised controlled trial
SHEAP: Statistical and health economics analysis plan
TSC: Trial steering committee
UHCW: University Hospitals Coventry & Warwickshire
WCTU: Warwick Clinical Trials Unit
WHO: World Health Organisation
6MWT: Six-minute walk test
