# Supplementary material for: Protocol update for a multi-centre randomised controlled trial of exercise rehabilitation for people with pulmonary hypertension: the SPHERe trial
Source: Trials. 2024 Jul 20;25:495. doi: 10.1186/s13063-024-08341-0 (PMC11264996; doi:10.1186/s13063-024-08341-0)
Supplement: Supplementary file 1 — Supplementary Material 1. [file 13063_2024_8341_MOESM1_ESM.zip › SPHERe Protocol V9.0R1.docx]

| 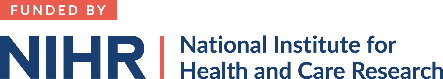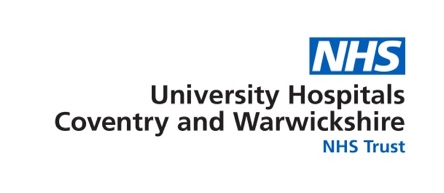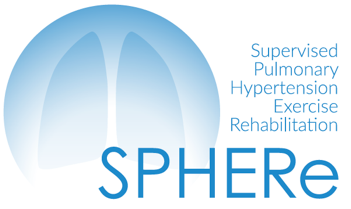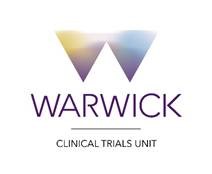 | | | | |
| --- | --- | --- | --- | --- |
| **PROTOCOL** | | | | |
| Supervised Pulmonary Hypertension Exercise REhabilitation (SPHERe): a multi-centre randomised controlled trial | | | | |
|  | |  | | |
| ISRCTN Number: | | ISRCTN 10608766 | | |
| Sponsor: | | UHCW NHS Trust | | |
| Funding Body: | | NIHR Health Technology Assessment (HTA) programme (HTA: 17/129/02) | | |
| IRAS ID: | | 261218 | | |
| Ethics Approval date: | | West Midlands- Coventry & Warwickshire REC, approved 13Jul2019 | | |
| Version Number: | | 9.0 | | |
| Date: | | 21 Sep 2023 | | |
| Stage: | | Final | | |
|  | |  | | |
| This protocol has regard for current HRA guidance and content.  **Protocol Amendments:** | | | | |
| **Version No.** |  | **Date** |  | **Date of Approval**  **(REC & HRA)** |
| 1.0 |  | 12 Apr 2019 |  | 30 Jul 2019 |
| 2.0 |  | 08 Jan 2020 |  | Substantial Amendment 1- 25 Feb 2020 |
| 3.0 |  | 01 Dec 2020 |  | Substantial Amendment 3- 20 Jan 2021 |
| 4.0 |  | 03 Feb 2021 |  | Non Substantial Amendment 5- 24 Mar 2021 |
| 5.0 |  | 04 Oct 2021 |  | Non Substantial Amendment 9- 07 Oct 2021 |
| 6.0 |  | 10 Nov 2021 |  | Substantial Amendment 4- 31 Jan 2022 |
| 7.0 |  | 06 Apr 2022 |  | Substantial Amendment 6- 05 May 2022 |
| 8.0 |  | 01 Nov 2022 |  | Substantial Amendment 7- 08 Feb 2023 |
| 9.0 |  | 21 Sep 2023 |  | Non Substantial Amendment 16- 13 Oct 2023 |

*This study/project is funded by the National Institute for Health Research (NIHR) HTA Programme* HTA: 17/129/02*. The views expressed are those of the author(s) and not necessarily those of the NIHR or the Department of Health and Social Care.*

**Contact NAMES and numbers**

| Role | Name, address, telephone |
| --- | --- |
| **Sponsor:** | UHCW NHS Trust  R&D Department  Tel: 02476 966 195  Email: [ResearchSponsorship@uhcw.nhs.uk](mailto:ResearchSponsorship@uhcw.nhs.uk) |
| **Chief Investigator:** | Dr Gordon McGregor  Warwick Clinical Trials Unit  The University of Warwick  Gibbet Hill Road  Coventry CV4 7AL  Email: [gordon.mcgregor@warwick.ac.uk](mailto:gordon.mcgregor@warwick.ac.uk) |
| **Senior Project Manager:** | Rowena Williams  Warwick Clinical Trials Unit  Email: [rowena.williams@warwick.ac.uk](mailto:rowena.williams@warwick.ac.uk) |
| **Trial Coordinator:** | Pritpal Klear  Warwick Clinical Trials Unit  Tel: 02476 150 300  Email: [sphere@warwick.ac.uk](mailto:sphere@warwick.ac.uk) |
| **Co-investigators:** | Professor Julie Bruce  Professor of Clinical Trials  Warwick Clinical Trials Unit  Email: [Julie.Bruce@warwick.ac.uk](mailto:Julie.Bruce@warwick.ac.uk)  Professor Martin Underwood  Professor of Primary Care Research  Warwick Clinical Trials Unit  Email: [m.underwood@warwick.ac.uk](mailto:m.underwood@warwick.ac.uk)  Professor Harbinder Sandhu  Professor of Health Psychology  Warwick Clinical Trials Unit  Email: [harbinder.K.Sandhu@warwick.ac.uk](mailto:harbinder.K.Sandhu@warwick.ac.uk)  Professor Kate Seers  Professor of Health Research  Warwick Research in Nursing, Health Sciences Division  Email: [kate.seers@warwick.ac.uk](mailto:kate.seers@warwick.ac.uk)  Professor Tamar Pincus  Professor of Health Psychology  Royal Holloway University of London  Email: [t.pincus@rhul.ac.uk](mailto:t.pincus@rhul.ac.uk)  Professor Sally Singh  Head of Cardiac & Pulmonary Rehabilitation  University Hospitals of Leicester NHS Trust  Email: [sally.singh@uhl-tr.nhs.uk](mailto:sally.singh@uhl-tr.nhs.uk)  Professor Stephanie Taylor  Professor in Public Health & Primary Care  Barts & the London School of Medicine & Dentistry  Email: [s.j.c.taylor@qmul.ac.uk](mailto:s.j.c.taylor@qmul.ac.uk)  Professor Prithwish Banerjee  Consultant Cardiologist  UHCW NHS Trust  Email: [Prithwish.Banerjee@uhcw.nhs.uk](mailto:Prithwish.Banerjee@uhcw.nhs.uk) |
|  | Dr Sarah Bowater  Consultant Cardiologist  UHB NHS Trust  Email: [Sarah.Bowater@uhb.nhs.uk](mailto:Sarah.Bowater@uhb.nhs.uk)  Dr Paul Clift  Consultant Cardiologist  UHB NHS Trust  Email: [Paul.Clift@uhb.nhs.uk](mailto:Paul.Clift@uhb.nhs.uk) |
| **Statisticians:** | Professor Ranjit Lall  Professor of Clinical Trials and Biostatistics  Warwick Clinical Trials Unit  Email: [R.Lall@warwick.ac.uk](mailto:R.Lall@warwick.ac.uk)  Dr Chen Ji  Senior Research Fellow - Statistics  Warwick Clinical Trials Unit  Email: [C.Ji.3@warwick.ac.uk](mailto:C.Ji.3@warwick.ac.uk)  Mrs Mariam Ratna  Research Fellow (Statistician)  Warwick Clinical Trials Unit  Email: Mariam.ratna@warwick.ac.uk |
| **Health Economists:** | Professor James Mason  Professor of Health Economics  Warwick Medical School  Email: [J.Mason@warwick.ac.uk](mailto:J.Mason@warwick.ac.uk)  Dr Rebecca Kandiyali  Associate Professor Warwick Medical School  Email: [Rebecca.Kandiyali@warwick.ac.uk](mailto:Rebecca.Kandiyali@warwick.ac.uk) |
| **Trial Steering Committee:**  **Independent Members:**  **Non-independent Members:** | **Chair:** Dr Ivonne Solis-Trapala Senior Lecturer in Medical Statistics Keele Clinical Trials Unit  Tel: 01782 734 711  Email: [i.solis-trapala@keele.ac.uk](https://email.uhcw.nhs.uk/owa/redir.aspx?C=4W2B1ZU0pez5dDNjzn5UMS3VMqnkXXGrBV7yMUXJERILEd4yPYzWCA..&URL=mailto%3ai.solis-trapala%40keele.ac.uk)  Dr James Oliver  Consultant Cardiologist  Leeds Teaching Hospitals NHS Trust  Tel: 0121 414 7065  Email: [james.oliver5@nhs.net](mailto:james.oliver5@nhs.net)  Dr Raymond Oppong  Health Economist  University of Birmingham  Tel: 07905 962 739  Email: [r.a.oppong@bham.ac.uk](mailto:r.a.oppong@bham.ac.uk)  Katie Dowling  Specialist Physiotherapist  Royal Brompton and Harefield NHS Trust  Tel: 0330 128 3615  Email: [k.dowling1@nhs.net](mailto:k.dowling1@nhs.net)  Rinku Puri  PPI Representative  Email: [rinku.puri@covwarkpt.nhs.uk](mailto:rinku.puri@covwarkpt.nhs.uk)  Dr Gordon McGregor  Warwick Clinical Trials Unit  The University of Warwick  Gibbet Hill Road  Coventry CV4 7AL  Email: [gordon.mcgregor@warwick.ac.uk](mailto:gordon.mcgregor@warwick.ac.uk) |
| **Data Monitoring Committee:**  **Independent Members:**  **Non-independent Members:** | **Chair:** Professor Stephen Walters Statistician University of Sheffield  Telephone: 0114 2220730  Email: [s.j.walters@sheffield.ac.uk](mailto:s.j.walters@sheffield.ac.uk)  Dr Joanna Pepke-Zaba Consultant Respiratory Physician  Royal Papworth Hospital NHS Foundation Trust  Tel: 01480 364230  Email: [joanna.pepke-zaba@nhs.net](mailto:joanna.pepke-zaba@nhs.net)  Professor Garry Tew  Professor of Clinical Exercise Science  York St John University  Tel: 01904 876 967  Email: [g.tew@yorksj.ac.uk](mailto:g.tew@yorksj.ac.uk)  Professor Ranjit Lall  Professor of Clinical Trials and Biostatistics  Warwick Clinical Trials Unit  Email: [R.Lall@warwick.ac.uk](mailto:R.Lall@warwick.ac.uk) |
| For general queries and supply of trial materials, please contact the coordinating centre:  Warwick Clinical Trials Unit (WCTU)  The University of Warwick  Gibbet Hill Road  Coventry  CV4 7AL  Tel: 02476 150 300  Email: [SPHERE@warwick.ac.uk](mailto:SPHERE@warwick.ac.uk) | |
| **Randomisation:** | Web system, available 24/7: https://ctu.warwick.ac.uk/SPHERe  Randomisation Telephone Line: 02476 150 402 (Mon-Fri, 9am to 5pm) |

| TABLE OF CONTENTS | Page |
| --- | --- |

TABLE OF CONTENTS 8

TRIAL SUMMARY 11

List of abbreviations/GLOSSARY 12

1. Background 13

1.1 Epidemiology and Burden of the Condition 13

1.2 Existing Knowledge 13

1.3 Hypothesis 15

1.4 Need for a Trial 15

1.5 Ethical Considerations 15

1.6 Consort 16

2. Trial Design 16

2.1 Pre-pilot Feasibility Trial 16

2.2 Trial Summary and Flow Diagram 16

2.3 Aims and Objectives 19

2.3.1 Objectives 19

2.4 Outcome Measures 19

2.4.1 Efficacy 19

2.4.2 Safety 21

2.4.3 Management of COVID-19 Transmission Risk 21

2.5 Eligibility Criteria 22

2.5.1 Inclusion Criteria 22

2.5.2 Exclusion Criteria 23

2.6 Participant Identification/Screening 24

2.6.1 Participant Identification Centres (PIC) 25

2.7 Consent 28

2.7.1 Consent for Qualitative Participant Interviews 29

2.7.2 Consent for Photographs and Video Clips 29

2.7.3 Consent for Qualitative Practitioner Interviews 29

2.8 Randomisation 29

2.8.1 Randomisation 29

2.8.2 Post-randomisation Withdrawals, Discontinuation of Treatment, and Lost to Follow Up 30

2.8.3 4 and 12 Month Follow-up Assessments (+/- four weeks) 30

2.9 Trial Treatments/Intervention 31

2.9.1 Trial Treatment(s)/Intervention 31

2.9.2 Compliance with Intervention/Contamination 35

2.10 Sites 35

2.11 Allocation Concealment 36

2.12 Site Staff Training 36

2.13 Concomitant Medication 37

2.14 End of Trial 37

3. METHods and assessments 37

3.1 Schedule of Data Collection 37

3.2 Longer Term Follow-up Assessments (England and Wales only) 38

3.3 Process Evaluation 38

4. adverse event management 39

4.1 Definitions 39

4.1.1 Adverse Events (AE) 39

4.1.2 Serious Adverse Events (SAEs) 39

4.2 Reporting Related and Unexpected SAEs 41

4.3 Responsibilities 42

4.4 Notification of Deaths 43

4.5 Reporting Urgent Safety Measures 43

5. Data management 43

5.1 Data Collection and Management 43

5.2 Database 44

5.3 Online Platform 44

5.4 One-to-one Consultations/Weekly Catch-ups Platform 44

5.5 Data Storage 44

5.6 Data Access and Quality Assurance 45

5.7 Data Shared with Third Parties 45

5.8 Archiving 45

6. Statistical analysis 45

6.1 Power and Sample Size 45

6.1.1 Revised Sample Size 47

6.2 Statistical Analysis of Efficacy and Harms 47

6.2.1 Statistics and Data Analysis 47

6.2.2 Planned Recruitment Rate 47

6.2.3 Statistical Analysis Plan 48

6.2.3.1 Summary of Baseline Data and Flow of Participants 48

6.2.3.2 Primary Outcome Analysis 48

6.3 Subgroup Analyses 48

6.4 Subject Population 49

6.5 Procedure(s) to Account for Missing or Spurious Data 49

6.6 Qualitative Data Analysis 49

6.7 Health Economic Evaluation 50

7 Trial organisation and oversight 51

7.1 Sponsor and Governance Arrangements 51

7.2 Ethical Approval 51

7.3 Trial Registration 51

7.4 Notification of Serious Breaches to GCP and/or Trial Protocol 52

7.5 Indemnity 52

7.6 Administration 52

7.7 Trial Management Group (TMG) 52

7.8 Trial Steering Committee (TSC) 52

7.9 Data Monitoring Committee (DMC) 52

7.10 Essential Documentation 53

7.11 Financial Support 53

8 Monitoring, AUDIT AND INSPECTION 53

9 Patient and Public InvolvEment (PPI) 54

10 Dissemination and publication 54

11 References 56

**LIST OF TABLES AND FIGURES PAGE**

Figure 1. Trial flow diagram 18

Figure 2. Participant identification and approach flow-chart……………………………………………..27

Table 1. Data collected at trial time points 37

Table 2. SAE causal relationship 41

## TRIAL SUMMARY

| **Trial Title** | Supervised Pulmonary Hypertension Exercise Rehabilitation: a multicentre RCT |
| --- | --- |
| **Sponsor ref. number** | SPHERe/GM427119 |
| **Clinical Phase** | Phase III |
| **Trial Design** | Multi-centre randomised controlled trial with embedded process evaluation and health economic evaluation |
| **Trial Participants** | Adults with pulmonary hypertension |
| **Planned sample size** | 200 (originally 352) people randomly allocated to receive the SPHERe intervention or active control |
| **Treatment Duration** | Maximum 4 months post randomisation |
| **Follow-up Duration** | 12 months post randomisation  (5 years postal follow-up, outside trial) |
| **Planned Trial Period** | 01 Jun 2019 to 31 Aug 2022 (39 months), extended to 29/02/2024 (57 months) |
| **Objectives** | To run a definitive multi-centre RCT testing the clinical and cost-effectiveness of SPHERe vs. best-practice usual care, including:   1. A pre-pilot to confirm feasibility, refine intervention delivery and manualised practitioner training, and prepare trial set-up at selected centres; 2. An internal pilot, with formative process evaluation, at a sample of out-patient centres to test recruitment and trial procedures; 3. A main trial with embedded process evaluation. |
| **Outcomes**  **Primary**  **Secondary** | Assessed at baseline, four months (post-randomisation) and 12 months  Incremental shuttle walk test at four months   1. Cambridge Pulmonary Hypertension Outcome Review 2. Hospital Anxiety and Depression Scale 3. Generalised self-efficacy scale 4. Fatigue Severity Scale 5. WHO functional class 6. Medication use 7. Time to clinical worsening 8. Hospital admissions 9. Adverse events 10. All-cause mortality 11. EQ-5D-5L 12. Health and care resource use |
| **Qualitative objective** | To explore and contextualise participant and practitioner experience, barriers and enablers, to inform interpretation of quantitative data and facilitate wider implementation |
| **Qualitative outcomes** | Semi-structured interviews with participants and practitioners |

List of abbreviations/GLOSSARY

| Abbreviation | Explanation |
| --- | --- |
| ACHD | Adult congenital heart disease |
| AE | Adverse Event |
| CAMPHOR | Cambridge Pulmonary Hypertension Outcome Review |
| CI | Chief Investigator |
| CONSORT | Consolidated *S*tandards *o*f *R*eporting *T*rials |
| CRF | Case Report Form |
| CTU | Clinical Trials Unit |
| CTEP | Chronic thromboembolic pulmonary hypertension |
| DMC | Data Monitoring Committee |
| FSS | Fatigue severity scale |
| GCP | Good Clinical Practice |
| HADS | Hospital Anxiety and Depression Scale |
| IRAS | Integrated Research Application System |
| ISRCTN | International Standard Randomised Controlled Trial Number |
| ISWT | Incremental shuttle walk test |
| NICE | National Institute for health and Care Excellence |
| MRC | Medical Research Council |
| PAH | Pulmonary Arterial Hypertension |
| PH | Pulmonary hypertension |
| PI | Principal investigator |
| PPI | Patient & Public Involvement |
| PPMO | Performance and Programme Management Office |
| QoL | Quality of Life |
| RCT | Randomised controlled trial |
| REC | Research Ethics Committee |
| R&D | Research and Development |
| SAE | Serious Adverse Event |
| SOP | Standard Operating Procedure |
| TSC | Trial Steering Committee |
| UHCW | University Hospitals Coventry & Warwickshire |
| WCTU | Warwick Clinical Trials Unit |
| 6MWT | Six-minute walk test |

# Background

## Epidemiology and Burden of the Condition

Pulmonary hypertension (PH) is a debilitating long-term condition characterised by severe exercise intolerance [1]. Pulmonary arterial pressure is abnormally raised due to dysfunctional endothelial cells and vascular smooth muscle, leading to maladaptive pulmonary vascular remodelling and increased right ventricular afterload [2, 3]. Pulmonary and cardiovascular haemodynamics are progressively compromised, often during minimal physical exertion [4, 5]. Consequently, exertional dyspnoea, fatigue and syncope are the most common symptoms, impacting profoundly on quality of life (QoL), morbidity and mortality [6].

Guidance from the World Symposium on Pulmonary Hypertension [7] identified five distinct subgroups:

Group 1 - Pulmonary arterial hypertension (PAH)

Group 2 - PH due to left heart disease

Group 3 - PH due to lung diseases or hypoxia, or both

Group 4 - Chronic thromboembolic pulmonary hypertension (CTEPH)

Group 5 - PH with unclear multifactorial mechanisms.

Drug treatment and pulmonary endarterectomy may help people with PAH [8] and CTEPH [9], respectively, but benefit is often limited. For people with PH secondary to cardiac or pulmonary disease (groups 2 & 3), there are no specific treatments of proven benefit [10, 11].

## Existing Knowledge

There are many similarities between PH and conditions like chronic obstructive pulmonary disease (COPD) and chronic heart failure (CHF); indeed, they often co-exist [6]. For people living with these conditions, exercise rehabilitation is recommended by NICE [12], and the British Thoracic Society [13], supported by a considerable evidence base [14]. Exercise rehabilitation can improve fitness in these populations, and increase ability to ‘self-manage’, often reducing health and care utilisation [14, 15]. Thus, it is plausible, that exercise may also help people with PH groups 2 & 3 due to underlying cardiac and pulmonary disease [6, 11]. In PH, exercise rehabilitation appears to be safe and may help people with PAH and CTEPH, particularly when undertaken as an in-patient [3]. Recent recommendations support a conservative approach, under the supervision of appropriately skilled practitioners [16, 17]. However, exercise rehabilitation has not yet been adequately tested in PH groups 2 & 3, or in an out-patient setting in the UK [10, 11].

A 2017 Cochrane review of exercise rehabilitation for PH identified six RCTs (N=206 mainly people with PAH or CTEPH) with short follow-up (3-15 weeks) [18]. Low quality evidence showed that exercise rehabilitation programmes increased six-minute walk test (6MWT) distance by 60m, compared to usual care (95% CI 30m to 90m), without any serious adverse events [19]. The SF-36 physical component score improved by 4.63 points (95% CI 0.80 to 8.47), which the review authors did not consider clinically important. Updating this review, identified one further trial (n=40) with eight-week follow-up [20], two ongoing trials with published protocols [21, 22], and seven trial registry entries, testing exercise rehabilitation in PAH or CTEPH. Few studies have tested exercise rehabilitation for PH groups 2 & 3 [4, 23], and there is only one ongoing trial according to trial registries (extension of existing long-term recruitment trial using an in-patient intervention protocol [24]).

A 2018 review examined the specific components and reporting quality of exercise interventions in 19 RCTs and non-randomised studies [25]. The highest quality reporting and best outcomes came from two studies at one centre using a three-week residential exercise intervention prior to 12 weeks of home exercise [24, 26]. Distance on 6MWT improved at three weeks; 111m (95% CI 65m to 139m) and 41m (no 95% CI) respectively. These clinically important benefits were maintained at 15 weeks. Current data do not confirm the effectiveness, or safety, of out-patient/community outreach exercise rehabilitation, or report on any outcomes beyond 15 weeks. The exercise interventions were well described, but only one of 19 studies adequately described behavioural or motivational strategies aimed at improving exercise adherence and compliance.

Exercise training might increase the risk of serious adverse events for some people with PH, due to reduced cardiac output, arrhythmias, pulmonary venous congestion, and hypoxemia [5]. Historically, there has been a reluctance to provide exercise rehabilitation for this population [27], and many patients were advised against exercise, leading to heightened anxiety. As such, modifiable psychosocial variables such as depression, anxiety and/or fear of exercise should be addressed when treating PH, as they are equally important as physical factors at predicting health-related outcomes in people living with PAH and CTEPH [28, 29]. These modifiable factors are also likely to be relevant for people with PH groups 2, 3, or 5.

**COVID-19:** In light of the COVID-19 pandemic the SPHERe intervention will move to an online home-based delivery model. In compiling the content of the home-based exercise intervention, we have drawn on existing resources and data from home-based rehabilitation programmes aimed at breathless, fatigued and anxious clinical populations. With a facilitated, functional (body weight or chair-based) exercise prescription, the REACH-HF trial (N=185) showed a clinically meaningful between-group difference in QoL at 12 months compared to usual care (MLHFQ score – 5.7 points [95% CI – 10.6 to – 0.7], p.0.025) [30, 31] in people with systolic heart failure. Likewise, the feasibility of facilitated, home programmes has been demonstrated in angina [32] and ischaemic heart disease [33].

Data supporting the feasibility and potential efficacy of home-based versus centre-based programmes is also available in COPD. Using a minimal resource approach, a between-group difference (N=166) for dyspnoea-related QoL (Chronic Respiratory Disease Questionnaire, CRQ) confirmed the non-inferiority of supervised, home-based rehabilitation on completion of the programme (1.6 points, [95% CI − 0.3 to 3.5]) [34]. Further, with a structured, unsupervised programme (N=287), whilst CRQ improved in both groups, there was inconclusive evidence that home-based rehabilitation was non-inferior to centre-based rehabilitation for improving dyspnoea (−0.24, [95% CI −0.61 to 0.12], p=0.18) at 7 weeks [35], indicating the importance of supervision.

Consideration of the available evidence in COPD, heart failure, angina and ischaemic heart disease, therefore, informed the design of the SPHERe home-based intervention. We concluded that SPHERe should be **structured and resource based** (manual, online content) using **functional (body weight or chair-based) exercise** and a structured home-based **exercise bike** programme which must be **remotely supervised and facilitated by trained practitioners.**

## Hypothesis

We will run a multi-centre RCT to test if SPHERe, a programme of remotely supervised, home based exercise rehabilitation, with psychosocial and motivational support, can improve walking distance and QoL, compared to best practice usual care, in people with PH (particularly groups 2 & 3).

We hypothesise that the SPHERe intervention will improve clinical, and patient reported, outcomes when compared to best practice usual care.

## Need for a Trial

In-patient exercise rehabilitation may have a short-term benefit on exercise capacity in selected people with PAH or CTEPH. However, it is not known if these benefits extend to PH groups 2, 3, & 5, if exercise rehabilitation delivered in an NHS out-patient/home based setting is effective or cost effective, or if there are any long-term health benefits or harms. Further, current exercise rehabilitation interventions for PH do not explicitly target modifiable psychosocial factors. To address these evidence gaps, a definitive RCT is required.

## Ethical Considerations

The trial will be conducted in full conformance with the principles of the Declaration of Helsinki and Good Clinical Practice (GCP) guidelines. It will also comply with all applicable UK legislation and University of Warwick Standard Operating Procedures (SOPs). All data will be stored securely and held in accordance with the General Data Protection Regulation.

Before enrolling people into the trial, each trial site will ensure that the local conduct of the trial has the agreement of the relevant NHS Trust Research & Development (R&D) department. Sites will not be permitted to enrol people into the trial until written confirmation of R&D agreement is received by Warwick Clinical Trials Unit (WCTU).

Trial staff will ensure that participants’ anonymity is maintained. At WCTU participant identifiable information will be stored securely on the electronic database and when in paper form, will be stored separately from CRFs using only that person’s participant ID number. SPHERe practitioners at UHCW will keep paper records of participant contact details and medical notes stored securely in locked filing cabinets. All documents will be stored securely and will only be accessed by trial staff and authorised personnel. The trial will comply with relevant UK data protection legislation, which requires data to be pseudo-anonymised as soon as it is practical to do so.

Data will be collected on paper CRFs at assessments appointments, or entered directly into a secure online database provided by WCTU. Paper CRFs and questionnaires will be posted and stored on site at WCTU under locked conditions for the duration of the trial; these will be considered source documents for the trial. Sites may also scan and send these to the SPHERe resource email account. Direct access to source data and documents will be granted to authorised representatives from the sponsor, host institutions and the regulatory authorities to permit trial related monitoring, audits and inspections.

All approaches to potential participants will come from clinical teams involved in their care, or a clinically trained member of the SPHERe team (for PIC sites). Where screening activity will be done by someone not involved in the direct care team, letters of access will be in place and approval will be sought from the Confidentiality Advisory Committee for section 251 support.

All individual data will be maintained within NHS sites until participants have agreed to provide the trial team with their personal details. By completing an expression of interest (EOI) form, patients are agreeing to provide the trial team with their contact details pre-consent.

Participants who are not fluent in written English will be eligible to take part. The primary outcome is a measure of exercise capacity which, unlike patient reported outcomes, does not require literacy; fluency in spoken English, however, is required for trial entry. This group may have problems reading trial material. When confirming consent for those unable to read English, a second person will be present to ensure correct explanation. The CAMPHOR and the EQ-5D-5L questionnaires will be collected orally, where necessary, to ensure that those unable to read English are able to contribute participant reported outcomes to the trial.

For adults lacking capacity to consent, e.g. people with PH secondary to Down’s syndrome but who are able to participate in the SPHERe intervention, we will seek advice from a personal consultee on whether they would wish to be included in our research trial. This applies to sites in England only- adults lacking capacity will not be recruited in Scotland.

Historically there have been concerns that exercise might involve an element of risk for people with PH. Recent evidence, however, does not indicate any increased risk of death during exercise for people with PAH & CTEPH. Nevertheless, deaths will be closely monitored in all trial participants and these data presented to the DMC regularly. A robust safety reporting procedure will be in place, in accordance with WCTU SOPs, to ensure participant safety and well-being are protected.

## Consort

The trial will be reported in line with the CONSORT (Consolidated Standards *o*f *R*eporting *T*rials) statement[36].

# Trial Design

## Pre-pilot Feasibility Trial

A pre-pilot feasibility phase (n= 6-10) will be undertaken to complete development of intervention and trial materials, refine recruitment processes, pilot practitioner training, and confirm feasibility of intervention delivery. Over a three month period, the constituent parts of the SPHERe intervention will be tested with six to ten participants recruited from up to three centres. The purpose of the pre-pilot feasibility will be to refine and test intervention and control materials, including participant (see section 2.4.2.1) and practitioner manuals, and staff training procedures, and to commence preparation for trial set-up at selected centres. This will allow us to confirm the feasibility of all aspects of the trial and make final alterations prior to the internal pilot.

## Trial Summary and Flow Diagram

The SPHERe intervention will be produced and refined during a six-month development phase. A subsequent pre-pilot feasibility phase will be undertaken to complete development, refine recruitment processes, pilot practitioner training, and confirm feasibility of intervention delivery. Subsequently, in an internal pilot at multiple sites, trial recruitment and retention will be confirmed. This will also provide provisional data on the fidelity of the intervention, its safety, and participant compliance and experiences. Finally, a multi-centre RCT with an embedded process evaluation will be conducted at up to 20 NHS exercise rehabilitation centres principally in the East and West Midlands.

By the end of the internal pilot we aim to have recruited at least 60 participants, set up at least 8 centres, and be recruiting 27 participants per month in total from all centres combined. We will, at this time, review progress against these three targets. As a benchmark, if we achieve less than 50%, the trial may be stopped. If we achieve 50% to 99%, we will critically review recruitment and centre set-up, report to TSC/DMC/HTA, implement significant remedial strategies and continue with a substantially modified protocol and close monitoring as required. If we achieve 100% the trial will continue with no modifications*.

*The internal pilot was completed on 15 Dec 2021, meeting the Amber target (50-99%) with 31 participants and 5 open centres. The trial moved into the main RCT phase thereafter, with remedial strategies to aid recruitment and centre set-up.

|  | **Red**  *<50%* | **Amber**  *50-99%* | **Green**  *100%* |
| --- | --- | --- | --- |
| **Recruitment** | n<30 | n=30-59 | n*≥*60 |
| **Number of centres open** | N<4 | n=4-7 | n*≥*8 |
| **Recruitment rate** | n<1.3/  centre/month | n=1.3-2.6/  centre/month | n*≥*2.7/  centre/month |

Trial overview: Adults with PH will be identified by the clinical care team, or a clinically trained member of the SPHERe team (for PIC sites), using multiple screening strategies, primarily via existing secondary care disease registers, out-patient clinic attendance, and hospital discharge data. Those with a formal PH diagnosis (European Society of Cardiology (ESC)/ European Respiratory Society (ERS) [17], and confirmed trial eligibility will be invited to participate.

We aim to recruit 200 (originally 352) participants from between 10 and 20 treatment centres, who will be randomised to the SPHERe intervention or best practice usual care on a 1.04:1 (cluster size= 5) and 1.10:1 (cluster size= 10) basis using a computer-generated randomisation sequence, performed by minimisation, based on centre, PH group, and World Health Organisation (WHO) functional class.

**Best practice usual care** will consist of an online individual practitioner appointment, with general advice on safe and effective physical activity.

The eight-week **SPHERe intervention** includes: 1) An hour long online individual assessment and exercise familiarisation session; 2) once weekly live online remotely supervised group home exercise programme 3) twice weekly guided home exercise bike and functional fitness programme; 4) weekly group online psychosocial and motivational support and education session (for 6 weeks). Practitioners will be provided with a comprehensive trial manual and will be fully supported by the trial team.

Outcomes will be assessed at baseline, four months (post randomisation) and 12 months. The primary outcome will be incremental shuttle walk (ISWT) distance at four months. The ISWT is an externally paced assessment of maximal exercise capacity which is sensitive to treatment effect, predicts mortality, and has no ceiling effect in PH [37]. Secondary outcomes will include health related quality of life (HR-QoL), clinical worsening, and a health economic analysis.

**Figure 1**: Trial Flow Diagram

**Screen/recruit**

**Baseline assessment**

**12-month outcomes assessment**

**4-month outcomes assessment**

**Control**

**intervention** (n=95)

**8-week SPHERe**

**intervention** (n=105)

**Randomise** (n=200)

  (1.10:1 ratio favouring intervention)*

**Inclusion**

1. Adults PH groups 1-5

2. Clinically stable

3. WHO class II-IV

4. English speaking

5. Travelling distance of centre

6. Able to make suitable travel arrangements to attend clinic

7. Access to IT infrastructure

8. Ability to consent

**Exclusion**

1. Exercise contra-indicated

2. Complications/co-morbidities preclude attendance

3. Significant mental health issue

4. Previous randomisation in present trial

5. Pregnant at time of recruitment

1. Individual assessment/familiarisation

2. Online remotely supervised home exercise sessions

3. Guided home exercise plan

4. Psychosocial/motivational support and education

1. Physical activity advice session

*Allocation ratio (for intervention vs control), in the sample size is based on 1.04:1 (cluster size =5) and 1.10:1 (cluster size =10). The above is on based on the ratio 1.10:1 (cluster size =10).

## Aims and Objectives

The aim of this trial is to assess the clinical and cost-effectiveness of remotely supervised, home-based pulmonary hypertension exercise rehabilitation (SPHERe) compared to best-practice usual care for people with pulmonary hypertension.

### Objectives

The objective of this trial is to run a definitive multi-centre RCT testing the clinical and cost-effectiveness of SPHERe vs. best-practice usual care, including:

1. A pre-pilot to confirm feasibility, refine intervention delivery and manualised practitioner training, and prepare trial set-up at selected centres;
2. An internal pilot, with formative process evaluation, at a sample of out-patient centres to test recruitment and trial procedures;
3. A main trial with embedded process evaluation.

## Outcome Measures

### Efficacy

**Primary Outcome:**

Exercise capacity as determined by distance walked in the incremental shuttle walk test (ISWT) at four months. ISWT will be performed as per European Respiratory Society (ERS)/American Thoracic Society (ATS) guidelines [19]. The externally paced ISWT is a simple assessment of maximal exercise capacity and, in PH, is sensitive to treatment effect, predicts mortality, and has no ceiling effect [37].

**Secondary Outcomes:**

1. Disease specific health-related quality of life (HR-QoL): Cambridge Pulmonary Hypertension Outcome Review (CAMPHOR) [38]. This is widely used as a clinical and research tool in PH, displaying good construct validity and reproducibility. It consists of a 25-item symptoms scale (scored 0–25), a 15-item functioning scale (scored 0–30) and a 25-item QoL scale (scored 0–25). For all scales, a low score indicates a better status [38].
2. Health utility: EQ-5D-5L [39]. A validated, generic HR-QoL measure consisting of five dimensions, each with five levels of response. Each combination of answers can be converted into a health utility score. It has good test-retest reliability, is simple to use, and gives a single preference based index value for health status that can be used for cost-effectiveness analysis.
3. Emotional well-being: Hospital Anxiety and Depression Scale (HADS) [40]. A 14-item screening questionnaire from which an anxiety and depression subscale can be derived. Sub-score values of 8 and above identify increased symptoms of anxiety and/or depression. Not used extensively in PH, but included as a well validated measure in clinical populations.
4. Generalised self-efficacy scale: a 10-item psychometric scale that is designed to assess optimistic self-beliefs to cope with a variety of difficult demands in life that are key targets of the behavioural component of the SPHERe intervention.
5. Fatigue: Fatigue Severity Scale (FSS) [41]. A nine-item questionnaire validated for evaluating disabling fatigue and previously used in PH [42]. Each item is rated on a seven-point scale, from strongly disagree to strongly agree. A total score is derived from all nine questions; a higher score indicates a greater impact of fatigue on everyday activities.
6. World Health Organisation (WHO) functional class: a modified New York Heart Association functional classification system adopted by WHO and used ubiquitously in PH. Participants are graded on their ability to perform physical tasks, and classified as (I) no limitation, (II) mild limitation, (III) marked limitation, (IV) unable to perform any activity [43]. This will be assessed by a research practitioner at each trial assessment.
7. Medication use: indication, class, drug name, dose and frequency of all regular medication will be recorded. Participants will be asked to bring their repeat prescription to outcome assessment appointments.
8. Time to clinical worsening: defined as one of; PH related death; listing for/completed lung transplant; hospitalisation for PH; clinical worsening leading to initiation of new PH treatment; decreased WHO functional class and ≥15% decrease in ISWT distance [44]
9. Health and social care resource use: participant self-report and NHS records. The primary health-economic analysis will concentrate on direct intervention and healthcare/personal social services costs, while wider impact (societal) costs will be included within the sensitivity analyses. Participants will complete resource use questionnaires at four and 12 month follow-up points, to collect resource use data associated with the interventions under examination. Participants may use a resource use diary as an aide memoire to help record their resource use between baseline and follow-up. At the end of the follow-up period a copy of the participant’s medical record will be requested from their GP. This will provide information on GP consultations and include copies of any hospital discharge letters allowing us to accurately cost in-patient care costs. Where appropriate, data will be triangulated from GP records, participant self-report, and data held in participating hospitals to achieve a robust estimate of health service activity.
10. All cause hospital admissions from GP records (see point 9 above).
11. Adverse events (see section 4.0 below).
12. All-cause mortality. Participants will be flagged with NHS digital to ensure notification of any deaths and cause of death both during the current trial and for longer term follow-up.

**Follow-up:** The primary outcome is an objective measure of exercise capacity. This means participants will need to attend the treatment centre for assessment at baseline, four months and 12 months. Patient reported outcomes will be collected at follow-up assessments. If any participants are unable to attend, a postal questionnaire will be used to collect patient reported outcomes. In the case of non-response, two key secondary outcomes (CAMPHOR and EQ-5D-5L) will be collected by phone. Fluency in written English is not an inclusion criterion for this trial. For those fluent in spoken, but not written English, CAMPHOR and EQ-5D-5L will be collected verbally at each follow-up. The EQ-5D-5L is well validated for verbal administration.

**Long-term follow-up:** Consent will be sought from participants (recruited in England and Wales) to keep their personal data and have access to their NHS data following the end of the trial. This will allow longer term postal follow up (for four years) to assess quality of life and to monitor deaths using NHS Digital data.

### Safety

SPHERe will be delivered online as remotely supervised home exercise sessions by experienced practitioners. All live exercise sessions will be delivered by exercise practitioners based at the Central Trial Hub- UHCW NHS Trust (Atrium Health). Live group exercise classes will allow real time supervision and instruction. At each live exercise session one practitioner will lead the exercise class and a designated ‘co-pilot’ will be immediately available to deal with any safety concerns or emergencies that arise. Each session will have a maximum of 8 attendees to ensure safety can be monitored. A safety protocol is in place for this at UHCW (see below).

Short instructional exercise video clips will be available for participants to view online through BEAMfeelgood website for the guided home exercise bike and functional fitness programme. Intervention practitioners will be specialist exercise physiologists or physiotherapists, experienced in assessment, prescription and delivery of exercise in high risk clinical populations. Training in the standardised delivery of SPHERe will be provided. WCTU and UHCW have extensive experience of training people to deliver complex interventions for chronic disorders. This, and experience of quality control of practitioner training, will ensure work is delivered to a high standard.

SPHERe outcomes assessments will be delivered in cardiopulmonary rehabilitation units with access to emergency equipment and qualified staff. Condition specific monitoring of exercise responses, as per cardio-pulmonary rehabilitation guidelines, will reduce and manage risk [13, 45, 46].

**See Section 4.0 for AE and SAE information.**

The online home-based exercise rehabilitation sessions with psychosocial and motivational support (intervention), and the single online session of one-to-one advice on safe and effective lifestyle physical activity (control) are not current practice across the NHS for the management of severe cardiopulmonary disease (i.e CHF and COPD). Consequently, as the interventions generally do not reflect current standard practice, participants may be exposed to additional exercise related risk over and above standard care currently received. In keeping with WCTU SOPs, a risk assessment and monitoring plan will be implemented, focusing on ensuring safe exercise assessment and prescription in PH. Primarily this will involve undertaking appropriate pre-exercise screening (as per existing guidelines [13, 46]), and application of existing exercise guidelines for CHF and severe COPD. Portable finger pulse oximeters will be provided for those identified during outcomes assessments as likely to desaturate during exercise with instructions on how to use these provided during the assessment appointment. Suitable home exercise equipment (exercise bikes) will be provided and participants will be advised where possible to have someone else at home with them when exercising.

### Management of COVID-19 Transmission Risk

During the COVID-19 pandemic, sites should follow their local Trust guidelines with regards to infection prevention and control measures including:

- Hand hygiene.
- Assessors wearing an appropriate level of PPE.
- Participants wearing face coverings.
- Appropriate cleaning of facilities including assessment rooms and equipment.
- Maintaining social distancing.

Sites should consider how best to manage patients attending for assessment appointments to reduce infection risk:

- Appointments at rehabilitation facilities should be one-to-one to minimise exposure.
- Minimise appointment length and on-site waiting time. Participants should arrive and go directly to their appointment where possible, thus minimising contact with health care staff.
- Restricting visitors.

The primary outcome for SPHERe is the incremental shuttle walk test (ISWT). It is important that this is completed under direct supervision in a rehabilitation facility. Participants will attend their nearest rehabilitation facility for an outcome assessment at three time-points (baseline, four months and 12 months).

During the telephone screening call, if initially eligible, sites should inform participants of the procedures for outcome assessment appointments, what to expect when attending and to allay any concerns they might have.

Dependent upon the COVID-19 pandemic status and Trust guidelines sites should consider minimising the time that participants spend on site. Sites can send the baseline questionnaire to participants to complete prior to them attending their initial assessment. Verbal consent for completion of the questionnaire will be obtained (and documented in source) if this does occur.

Once eligibility has been checked and written consent given, the participants will be asked to provide their completed questionnaire to the practitioner. If a patient is not eligible to take part or does not consent to the trial, they should take the baseline questionnaire away with them to be discarded. Participants can also be given the follow-up questionnaire at their follow-up appointment to take home, complete and post to Warwick Clinical Trials Unit.

**Participant transport to outcome assessment appointments**

Participant travel expenses to outcomes assessments will be reimbursed. During the COVID-19 pandemic participants are advised to use public transport at their own risk. Ideally participants should drive themselves, or be driven by a relative or friend, to appointments. Alternatively, they can be provided with a taxi. Sites should use taxi companies with clear infection control policies as per their local trust guidelines. Things to consider include:

- Black cab with partition between driver and occupants
- Cleaning of seats and handles before each customer
- Driver and occupant to wear face covering
- Driver to wait at facility for participant to finish appointment.

## Eligibility Criteria

People are eligible to be included in the trial if they meet the following criteria:

### Inclusion Criteria

1. Adults (18+) with confirmed PH (groups 1 to 5) as detailed in ESC/ERS guidelines [17].
2. Clinically stable: Groups 1, 4, & 5 - stable on optimal PH specific drug therapy (for those in whom it is appropriate) for at least 1 month, or evidence that these drugs cannot be tolerated. Groups 2 & 3 - stable on drug therapy for underlying cardiac or pulmonary disease for at least one month. Clinical stability will be confirmed by the lead practitioner at each site, in consultation with the responsible clinician, and determined as: presenting with, reproducible, manageable symptoms, not requiring any treatment other than routine follow-up care, and no PH related hospital admission in the last four weeks.
3. World Health Organisation (WHO) functional class II, III or IV. The modified New York Heart Association functional classification system adopted by WHO is used ubiquitously in PH. People are graded on ability to perform physical tasks, and classified as (I) no limitation, (II) mild limitation, (III) marked limitation, (IV) unable to perform any activity [43]. This will be determined by the lead practitioner further to contact with the patient.

*People in functional class IV will not be excluded as symptoms and functional ability can vary considerably over time. Instead, those most severely affected will be excluded, on the basis that they are too unwell to attend a SPHERe centre, or to undertake exercise training.*

1. Fluent in spoken English to allow engagement with intervention and physical outcome measures.
2. Live within reasonable travelling distance (as defined by the participant) of a SPHERe exercise rehabilitation centre (for outcome assessments only).
3. Able to make suitable travel arrangements to attend clinic (for outcome assessments only).
4. Access to appropriate IT infrastructure (computer, laptop, tablet, smart phone, email and internet connection).
5. Ability to provide informed consent.

For adults lacking capacity to consent, e.g. people with PH secondary to Down's syndrome but who are able to participate in the SPHERe intervention, we will seek advice from a personal consultee on whether they would wish to be included in our research trial. This applies to sites in England only- adults lacking capacity will not be recruited in Scotland.

### Exclusion Criteria

1. Absolute contra-indications to exercise as per international clinical guidelines [46, 47].
2. PH related complications, or comorbidities severe enough to prevent attendance at a SPHERe centre, or participation in exercise rehabilitation.
3. Any mental health issue that will prevent engagement with trial procedures.
4. Previous randomisation in the present trial.
5. Pregnant at time of recruitment.

## Participant Identification/Screening

**Clinical pathway:** the patient pathway from referral to diagnosis of PH is well defined in ESC/ERS guidelines [17], and is adopted by all SPHERe trial hospitals. The largest pool of potential participants are those not referred to specialist centres, but treated locally for underlying cardiac and pulmonary disease; i.e. predominantly groups 2 & 3 PH.

**Participant identification:** Participants will be identified and screened by the clinical care team , or a clinically trained member of the SPHERe team (for PIC sites), via multiple, co-ordinated screening strategies (see Figure 2):

1. *Local secondary care disease registers:* Groups 1 and 4 PH are typically well recorded on disease registers (regardless of referral history to a specialist centre).
2. *Hospital discharge data:* Diagnosis specific coded discharge data (Performance and Informatics teamor equivalent) identifies PH admissions.

Discharge data from all hospitals will be regularly screened, using via Trust reporting systems, to identify admissions of all diagnostic groups of PH. All Trusts use the generic NHS coding system. Clinical care teams at each hospital, or a clinically trained member of the SPHERe team (for PIC sites), will screen these data and identify potential participants. This strategy is currently employed clinically at all trial hospitals to identify people suitable for cardiopulmonary rehabilitation, meaning robust, effective systems are already in place.

1. *Specialist nurse/medical clinics:* People with less prevalent PH aetiology, not captured through other routes, are likely to attend the following clinics: Rheumatology, haematology, chronic heart failure, general cardiology, respiratory, adult congenital heart disease, nephrology, haemodialysis. At individual Trusts, research nurses will be allocated to support recruitment from these outpatient clinics.
2. *Liaise with all specialist PH centres*: To identify patients that live local to SPHERe centres who can be approached.
3. *Referrals to cardiopulmonary rehabilitation*: Staff running clinical services will be involved in SPHERe. They will identify and approach people referred for rehab, who have diagnosed PH.
4. *Echocardiogram records:* If necessary, clinical echocardiogram records will be searched for patients with a peak tricuspid regurgitation >3.4 m/s on transthoracic echocardiogram who have not already been approached. Treating clinicians will be contacted to ascertain if a subsequent diagnosis of PH was made and, if appropriate, ask them to contact the person about the trial. Because of the large number of such investigations performed, the low prevalence of PH, and the likelihood that such patients will already have been seen in a relevant clinic, this will be very labour intensive for a low yield. Nevertheless, if recruitment is slow, it is an additional strategy.
5. *GP or self-referral:* Most potential participants will be under the care of secondary/tertiary services; however, recruitment opportunities will be maximised by:
6. Displaying recruitment posters in clinical settings

Preliminary searches of GP record data indicate that the number of recorded diagnoses of PH are too few to make formal screening of GP records worthwhile. Nevertheless the trial will be promoted to patients by placing posters in GP surgeries and hospital/rehabilitation clinics, and interested patients will then call the SPHERe WCTU trial team.

1. The trial will be promoted though local media, social media, relevant charities and patient support groups and on the trial website. This may include circulation of posters. People living with PH will be able to self-refer.

As individual people may be identified from multiple sources within each hospital, screening logs will record who has been approached to reduce risk of multiple approaches.

Screening logs will be populated at each hospital, to provide accurate information on patient eligibility, and reasons for non-participation, to inform future NHS service design.

**Participant Approach at Participating Sites**

1. Clinical care team, or a member of the SPHERe team (for PIC sites), identify potential participants (as per recruitment strategy above). Where screening activity will be done by someone not involved in the direct care team, letters of access will be in place and approval will be sought from the Confidentiality Advisory Committee for section 251 support. The SPHERe team member will be clinically trained and qualified to confirm pulmonary hypertension diagnosis and initial eligibility.
2. Clinical care team, or a member of the SPHERe team (for PIC sites), screen recent electronic record to identify any obvious contra-indications to exercise.
3. If the potential participant may be eligible, confirmation of PH diagnosis will be sought from the relevant clinician.
4. Potential participant contacted in person (at physical or virtual clinic) or by invitation letter. An expression of interest form and PIL will be sent alongside the invitation letter.

**Telephone Screening**

1. Patients will return the expression of interest form to WCTU prior to consent.
2. WCTU will enter the contact details (collected on the expression of interest form) into the online database for participants who have expressed an interest in taking part.
3. WCTU will then send the screening numbers of any expression of interest forms received to the research teams at each site for telephone screening calls to be made.
4. If the patient does not respond within 2 weeks, the site should follow up with a phone call. If they did not receive the invitation letter, expression of interest form and PIL, a replacement will be sent. If the patient is interested in taking part in the trial, they can provide verbal expression of interest over the phone. The expression of interest form can then be completed on the patient’s behalf by the member of the research team making the call and scanned to sphere@warwick.ac.uk.
5. Research teams at each site will confirm patient eligibility during a telephone screening call and complete a telephone screening form.
6. If the patient is eligible, they will be invited to a baseline assessment appointment where the eligibility form will be completed, consent will be taken, the ISWT assessment will be performed and the participant will complete the baseline questionnaire.

### Participant Identification Centres (PIC)

PIC sites will also be used to identify potential research participants. PIC sites will identify potential participants, screen for initial eligibility as described above and send invitation letters, PIL and PIC expression of interest forms to potential participants. An experienced, clinical member of the SPHERe team (with appropriate letters of access) will support screening and sending invitation letters for PICs who lack capacity. This person will have access to hospital notes (electronic and paper) and patient personal data (including address and contact details). We will seek approval from the Confidentiality Advisory Committee for section 251 support before this pathway is implemented.

Telephone screening and face to face baseline and outcome assessments will be performed by staff at the nearest participating SPHERe NHS trust with capacity or the Central Trial Hub. Individual assessments and all other aspects of the trial will be delivered from the Central Trial Hub. Staff at the nearest participating NHS hospital trust with capacity will obtain contact details for interested participants, identified through PIC sites, by accessing the relevant contact details section of the WCTU online database.

**Figure 2:** Participant identification and approach flow-chart

Secondary care disease registers

Secondary care discharge data

Secondary care out-patient clinics

PH specialist centres registers

Echocardio-gram records

Cardiopul-monary rehab referrals

Posters (clinical settings)

Self referral

Letter sent by local clinical teams/member of SPHERe team (PIC sites only)

Clinical teams give information in person or send letter

Letter sent by specialist centre teams

Patients contact the trial team directly

BASELINE ASSESSMENT CLINIC (Face-to-face appointment)

Expression of interest

Expression of interest

## 2.7 Consent

**Informed consent:** Potential participants will be invited to a baseline assessment appointment where eligibility will be confirmed and consent will be taken in person by an appropriately trained member of the clinical or research team.

**Adults lacking capacity (England and Wales only):** MRC guidelines will be followed; <https://mrc.ukri.org/documents/pdf/medical-research-involving-adults-who-cannot-consent/>. The trial will be discussed with the potential participant's personal consultee from whom advice will be continually sought during the trial. Any relevant issues will be discussed with the consultee prior to the trial and clear accessible (to authorised personnel) records kept. The consultee will be kept fully informed during the trial and will be invited to attend all appointments to provide support to the participant. All trial information will be provided to the consultee who will be able to share and discuss it with the potential participant at an appropriate level. Clinical and research staff can be involved in these discussions at the discretion of the consultee. This applies to sites in England only- adults lacking capacity will not be recruited in Scotland.

**GP notification:** The participant’s GPs will be informed by letter that they are taking part in the trial

**Timing of consent:** At the baseline assessment appointment, written informed consent will be obtained by a suitably trained member of the research team at each site, as per the delegation log, after allowing sufficient time for the potential participant to consider their decision and ask questions about the trial. Sufficient time for some potential participants may result in a decision to take part in the trial immediately after receiving all the relevant information in clinic. Alternatively, if potential participants would like to leave the clinic with the information and decide later, they will be free to do so, and will be contacted by phone at least 24 hours later.

**Responsibility:** Local PIs will retain overall responsibility for informed consent at their site and will ensure that any person delegated responsibility to participate in the informed consent process is duly authorised, trained, qualified and competent.

When confirming consent for those unable to read English, a second person will be present to confirm correct explanation, i.e., family member or translator according to WCTU SOP 7. During the COVID-19 pandemic this is dependent on local practices at site.

**New information:** Any new information that arises during the trial that may affect participants’ willingness to take part will be reviewed by the TSC; if necessary this will be communicated to all participants. A revised consent form will be completed if necessary.

Incidental findings relating to participants' medical conditions or general health, will be discussed with the managing consultant, and communicated to the participant as required.

**Decline/withdrawal:** Participants (or personal consultee- England only) will have the option to withdraw before treatment starts (i.e. between baseline assessment and the beginning of the intervention/ control), if for any reason they change their mind. This will be recorded on a withdrawal form. The right of a potential participant to refuse participation without giving reasons will be respected and recorded on the screening log. A reason will be documented if participant is willing to offer one. The participant will remain free to withdraw at any time without giving reasons and without prejudice to any further treatment, and will be provided with a contact point where he/she may obtain further information about the trial.

### Consent for Qualitative Participant Interviews

At the beginning of the trial, participants will be asked for their consent to be contacted at a later stage about an interview with a researcher. If they are selected for an interview, a member of the WCTU trial team will contact the participant to discuss the interview study and answer any questions they may have. If the participant remains happy to proceed, a date will be arranged for the interview to take place. Consent will be taken before the interview is conducted by telephone or video call.

### Consent for Photographs and Video Clips

At the beginning of the trial, participants will be asked if they consent to have photographs or short video clips taken during the live exercise and motivational support sessions for use at conference presentations or for trial publicity. If they consent to this, selected participants/groups will be approached by a study practitioner and an appropriate time and date will be arranged for photos/recordings to be taken.

### Consent for Qualitative Practitioner Interviews

The SPHERe trial intervention will be delivered online from the Central Trial Hub – UHCW NHS Trust. There will be around 5 practitioners involved in delivery of the trial (control and intervention). Practitioners at other participating NHS Trusts will perform telephone screening calls, randomisation, consent and all outcome assessments. At the end of their time on the trial, and with their consent, we will interview all practitioners involved in the study. If more practitioners are involved in delivery, we will also interview them. Interviews will be conducted by a qualitative research fellow from Warwick CTU.

## 2.8 Randomisation

### 2.8.1 Randomisation

At the initial assessment appointment, full eligibility will be assessed and informed consent will be obtained. The participant will complete the baseline questionnaire (if not already completed) and will perform the ISWT test. Written consent for entry into the trial must be obtained and the baseline assessment performed prior to randomisation. The Baseline, Medications and Additional Details forms are to be completed and inputted by site into the database (these can be directly inputted). Following consent, participants will be asked to complete a baseline questionnaire. Completed questionnaires should be inputted directly into the database or a copy sent to WCTU*.

Randomisation can then take place on the online web-based platform: <https://ctu.warwick.ac.uk/sphere>. Staff members assigned for randomisation duties (on the delegation log) will be given their own username to log in. Randomisation will be undertaken using a computer-generated randomisation sequence, performed by minimisation, based on centre, PH group, and World Health Organisation (WHO) functional class. Allocation concealment will be maintained by using WCTU centralised randomisation service; with minimisation by centre, PH group (1-5), and WHO functional class [two categories: 1) Class II; 2) Class III or IV]. Where PH has multiple underlying aetiologies, the predominant diagnosis will be confirmed by the managing clinician.

Once randomised, the participant will be informed of their treatment allocation. Those randomised to Usual Care (control) will be given or sent the British Lung Foundation Keep Active Exercise Booklet, a Healthcare Use Diary (to complete prospectively) and an optional Appointment Record Sheet. The Central Trial Hub (UHCW) will contact the patient to organise their 1:1 online advice session.

Participants randomised to the intervention arm will be given a copy of the SPHERe Intervention Participant Workbook, Healthcare Use Diary, optional Appointment Record Sheet and be asked to sign an Exercise Bike Loan Agreement. The Central Trial Hub (UHCW) will contact the participant to organise their 1:1 online individual assessment, group exercise and psychosocial sessions.

Sites should ensure that they book the four and 12 month follow-up assessment appointments at the appropriate time giving participants appropriate notice. WCTU will send follow-up assessment appointment reminders to help sites with this. Please see section 2.8.3 for details on follow-up assessments.

## 2.8.2 Post-randomisation Withdrawals, Discontinuation of Treatment, and Lost to Follow Up

**Withdrawal:** Participants may decline to continue involvement in the trial at any time, without prejudice. This will not affect the standard of care they receive. They will be advised to discuss their ongoing treatment with their clinician. For participants withdrawing from the trial, data obtained prior to the point of withdrawal, will be retained for the final analysis unless explicitly withdrawn at the participant’s request. For participants who withdraw, the withdrawal form will be completed. Participants may be withdrawn from the trial, at any time, at the discretion of the investigator and/or TSC.

**Discontinuation of treatment:** Participants in the intervention arm can ask to discontinue the trial intervention. These participants are not classed as withdrawals and will continue to be followed-up for further data collection unless they specifically ask to withdraw from follow-up and/or routine data collection. Wherever possible, we will obtain a reason for discontinuation or withdrawal on trial CRFs, although the right of a participant who does not want to give reasons will be respected.

**Lost to follow up:** Those participants who cannot be contacted directly or via Next of Kin (NOK) for their 4 and/or 12 month assessments will remain in the trial unless they have specifically withdrawn. Their data will be obtained from their hospital and GP records where possible.

### 4 and 12 Month Follow-up Assessments (+/- four weeks)

These assessments should be done within +/- four weeks of the expected date (4 or 12 months post-randomisation).

At the follow-up clinic assessments, participants will perform the ISWT and complete the relevant questionnaires. Please note- participants should be given the Healthcare Use Diary at Randomisation to record healthcare use and report this at their follow-up assessment (4m questionnaire). At four months, participants can be given a new healthcare use diary (if needed) to record healthcare use between 4 and 12 months (collected in their 12m questionnaire). Completed questionnaires are to be inputted directly into the database or a copy sent to WCTU*.

The 4 and 12 month forms will be completed and site staff are to input this on the database (these can be directly inputted).

Sites should call the patient the following working day after their 4 or 12 month appointment to check for any adverse events. Please see Section 4 (Adverse Event Management) for further guidance.

If a participant does not attend their 4 or 12 month assessment they should be contacted again to re-organise this. If the participant cannot attend their face-to-face clinic assessment, then core outcomes (such as WHO functional class, adverse events and medication changes) should be collected over the telephone and the participant should be sent a questionnaire in the post (to complete and return to WCTU). Sites should add a comment to the relevant follow-up form on the database.

For each participant, their contact details will be recorded (e.g. address, telephone numbers, mobile telephone numbers, email addresses and contact details of their NOK) to prevent loss to follow up. This identifiable information will be held securely on a password protected database, accessible only to authorised personnel. The site should make three attempts (telephone, email and/or text) to contact the participant to book their 4/12 month assessments. If no success, the NOK should then be contacted to check participant status. If sites cannot get in touch with the participant or NOK then the participant will be lost to follow-up. Where possible, data will be obtained for these participants from their hospital and GP records. For participants lost to follow-up, the site is to add comment to relevant follow-up form on the database.

* Please **do not** send the last page (change of participant details) of the questionnaire to WCTU as this contains identifiable personal data.

## Trial Treatments/Intervention

### 2.9.1 Trial Treatment(s)/Intervention

**Format:** To ensure generalisability to the NHS, the underpinning framework of SPHERe is based on UK cardio-pulmonary rehabilitation guidelines and service delivery models [13, 45, 48], and enhanced with PH specific recommendations [13, 16, 17]. In light of the COVID-19 pandemic, all interventions will be delivered and supervised remotely online. This fits with existing policy drivers advocating development of remotely supervised programmes.

*Cardio-pulmonary rehabilitation:* Service design in the UK is heterogeneous, as is the case for SPHERe centres. Some centres provide separate cardiac and pulmonary rehabilitation programmes, whereas other centres combine these programmes. To maintain consistency of the intervention package, participants, regardless of PH aetiology, will be included, in a pulmonary rehabilitation programme framework wherever possible. Henceforth, this will be referred to as ‘cardio-pulmonary rehabilitation’.

*Programme design:* To maximise accessibility and resource, whilst ensuring that the benefits of group interaction are retained, SPHERe will be delivered as an online, remotely supervised programme. Participants randomised to the SPHERe intervention will join online sessions, exclusively with other SPHERe participants.

Exercise training will be facilitated by a trained SPHERe practitioner using:

1. Participant workbook with details of exercise programme, instruction on safe and effective exercise, and a logbook to record completed exercise. These materials will be provided to participants.
2. Short video clips of exercises being performed graded by ability, available online.
3. Live exercise sessions led by a SPHERe practitioner to allow participants to complete live exercise sessions with other participants and receive real time instruction and feedback.
4. Weekly one-to-one discussion with SPHERe practitioner to troubleshoot and adjust exercise prescription.
5. Loan of a home exercise bike for the duration of the intervention.
6. Loan of a finger pulse/O_2_ saturation device (pulse oximeter), where indicated, for the duration of the intervention.

The SPHERe intervention has **four components**:

**Component 1. Individual assessment and exercise familiarisation**

*Individual assessment:* This assessment is a one-to-one online appointment with a SPHERe ‘practitioner’ (specialist cardio-pulmonary clinical exercise physiologist or physiotherapist), independent of the research team. Participants will undergo an online ‘assessment’. This will include a thorough assessment of medical history, medication, exercise/physical activity history, and discussion of participant goals. This information will be documented on an Individual Assessment form.

*Familiarisation:* Exercise guidance, specific to the underlying PH aetiology, will be delivered on an individual basis during the individual assessment and will be reinforced throughout the exercise programme. This guidance will enable participants to build confidence, whilst SPHERe practitioners refine and optimise the exercise prescription. Practitioners will begin to introduce the principles of psychosocial and motivational support (see below – component 3) during this session. Exercise will initially be undertaken at a very low level whilst practitioners assess the participants response to exercise. This individual assessment/exercise prescription/familiarisation session will last one hour and be delivered online, one to one.

*Exercise prescription:* The SPHERe practitioner will prescribe a tailored, individualised exercise programme [13, 45, 46] within pre-specified parameters, as detailed in the practitioner manual. Clinical information, data from the individual assessment, and patient centred goal setting will be used to devise a safe and effective exercise prescription. As no formal exercise assessment will be undertaken from which exercise could be prescribed, practitioners will prescribe exercise based on participant report of activities of daily living and physical activity. Whilst not using data from the ISWT research outcome assessment to prescribe exercise, practitioners will have access to these reports to check for any exercise induced complications i.e. desaturation.

Participants will have a weekly one-to-one discussion with a SPHERe practitioner throughout the 8 week intervention to troubleshoot and adjust exercise prescription. During these appointments practitioners should ask participants to provide feedback on the exercise sessions they have attended/completed and record on the participant exercise log. They should also record the exercise prescription.

**Component 2. Online remotely Supervised group home-based exercise sessions**

Participants will undertake home based online group exercise sessions once per week. Sessions will be pre-determined and booked by the SPHERe team at the Central Trial Hub (UHCW). These sessions will focus on functional fitness training with a quantifiable and progressive dose of, multi-modal, aerobic, muscular strength and endurance, and ‘functional fitness’ exercise. Adequate warm-up and cool-down will be incorporated into sessions. Intensity will be monitored using rate of perceived exertion, dyspnoea scale and pulse oximetry (O_2_ saturation) (where indicated) [49]. These exercise sessions will be undertaken online exclusively for SPHERe participants delivered from a single central venue – UHCW NHS Trust by the SPHERe intervention practitioners (specialist exercise physiologists or physiotherapists).

If deemed necessary during the baseline assessment, some participants will also be loaned a finger pulse oximeter for the duration of the exercise programme.

The SPHERe exercise programme is optimised to be appropriate for a broad spectrum of patients including frailer, deconditioned, low-mobility, exercise-naive participants. It will target physical goals identified as important by patients: *“less breathless, less fatigue, do more, walk further, climb stairs, fitter, more independence”.* Equally, it will address their psychosocial priorities including: *“confidence to exercise, anxiety, motivation, frustration, social interaction, fun, engaging, something different”.*

These goals are unlikely to be satisfied by conventional cardiovascular exercise alone, so will be combined with ‘functional fitness’ training (described below). The programme will be highly adaptable to allow personalisation to lower or higher ability participants, whilst ensuring safety and efficacy.

Cardiovascular exercise can be effective in improving cardiorespiratory fitness, muscular strength and endurance. However, it can lack physiological and biomechanical specificity to activities of daily living. Standard cardiovascular exercise is performed almost exclusively in the longitudinal plane (forwards/ backwards). In addition, adherence to this form of exercise is commonly poor [50, 51]. Much of this is thought to relate to the repetitive nature of this mode of exercise, the lack of perceived application to activities of daily living, and the limited opportunity for constructive social contact. Engagement with, and adherence to, exercise is enhanced by social, fun activities [52].

To address both these issues, SPHERe combines conventional home-based aerobic exercise with ‘functional fitness training’. This uses multi-plane motion (rather than just longitudinal), to target not only cardiorespiratory fitness, but also essential pre-requisites of active, independent living; e.g. agility, co-ordination, proprioception, balance and functional strength [50]. In addition to a static home exercise bike, SPHERe will make use of readily available items that can be found in the home.

Central to SPHERe is the expertise and experience of the specialist cardio-pulmonary exercise physiologists and physiotherapist who will ensure holistic, safe and effective exercise training. This conforms to existing recommendations of specialist exercise supervision for this population [13, 16, 17].

**Component 3: Guided home exercise plan**

To complement the online exercise sessions, all participants will be provided with a home exercise plan to follow twice weekly including a functional fitness training programme and an exercise bike programme. Participants will have access to the videos showing these exercises being performed. The SPHERe Intervention Participant Workbook (see below) will include a log to record time spent exercising.

**Emergency procedure:** Participants will undertake live exercise and support sessions in discrete groups of up to 8 people. In advance of each session, the practitioner will have access to contact details for each participant, or consultee (England only) where applicable. During the sessions, the practitioner will be able to see each participant individually on a large screen. In the event of an emergency, the practitioner will alert the designated ‘co-pilot’ for the session who will be able to communicate directly with the participant in question (via the live call or telephone) outside of the group, and alert the emergency services if required.

**Component 4. Psychosocial and motivational support; and education:**

Once per week, participants will receive a group (same as their exercise group) online 60 minute psychosocial and motivational support session delivered by a SPHERe practitioner.

*Psychosocial and motivational support:* The aim is to improve short and long-term adherence to exercise, thus maximise benefit. As such, SPHERe will draw on social cognitive approaches to behaviour change [53], including scrutiny of multiple interactions between environment, personal factors and behaviours. Based on the COM-B framework, three basic aspects of peoples’ lives will be addressed: capability (increasing confidence through supervised practice), opportunity (identifying internal and external opportunities), and motivation (education, self-reflection, goal setting) [54]. There will be a focus on increasing participants’ awareness of their priorities, through an investigation of the pros and cons of changing a specific behaviour (self-management e.g. fear avoidance of exercise), and assisting them to develop a specific plan of changing behaviour (planning, goal setting). The SPHERe practitioners will be trained to use open questions and motivational interviewing to assess participants’ current beliefs and encourage behaviour change.

The live exercise and support sessions for the SPHERe trial will be delivered online from the Central Trial Hub (UHCW) via BEAMfeelgood. There will be approximately 5 SPHERe practitioners (Clinical Exercise Physiologists/Physiotherapists) who will deliver these live exercise and support sessions as described above. Additional SPHERe practitioners may be identified during the course of the trial.

**Best practice usual care/control intervention:** The control arm, will be an intervention that could be described as ‘best usual care’, is in the form of an individual online practitioner appointment with general advice on safe and effective physical activity in PH. This 30-minute online appointment will allow the practitioner to discuss individualised ways in which the participant can undertake physical activity at home. They will not be provided with a structured exercise plan, rather comprehensive freely available British Lung Foundation Keep Active Exercise and PR booklet detailing ways in which low level physical activity can be safely and effectively incorporated into their everyday lives. No specific psychological techniques will be used to support the provision of this information. This allows the usual care group to be offered best current practice, whilst retaining the aim of the trial; comparing a group who receive comprehensive PH exercise rehabilitation with psychosocial and motivational support, with a group who do not.

**SPHERe intervention participant workbook:**

This will be a comprehensive resource detailing, in a patient-friendly fashion, all information relating to the trial. It will be developed in the pre-pilot feasibility phase. In the form of a workbook, this resource will be introduced to the participant at the individual assessment and exercise familiarisation session. The workbook will include: 1) general information about the trial; 2) background information about PH; 3) schedule for the participant’s exercise programme, education and psychosocial and motivational sessions; 4) exercise log; 5) general advice on safe and effective lifestyle physical activity; 6) useful links and contacts; 7) IT advice to assist with accessing online SPHERe content and live sessions. Content will be developed with lay partners. It can also be provided electronically.

**Technological considerations:**

Participants will be advised of the minimum IT requirements for participation in the trial. Where required, the trial team will advise and instruct participants on the use of computers/devices before the individual assessment which is the first video call participants are expected to attend. For those who do not have the appropriate equipment, a loan tablet computer will be provided where possible to enable them to participate in the trial.

### Compliance with Intervention/Contamination

**Compliance:** Attendance at assessments and completion of intervention (individual assessment, online home exercise sessions, guided home exercise plan, and psychosocial/motivational sessions) and control sessions will be recorded as one measure of compliance. The impact of compliance on outcomes will be assessed using a CACE (compliers average causal effect) analysis.

For the intervention group, partial compliance will be defined as completion of the initial assessment/familiarisation and at least half of the, expected group online home exercise sessions, guided home exercise, and psychosocial/motivational sessions. Full compliance will be considered as attending at least 75% of online home exercise sessions, guided home exercise and psychosocial/motivational sessions. The psychosocial/motivational sessions, in themselves, are designed not only to help improve QoL, but equally to improve compliance and adherence.

**Fidelity:** The individual assessment will all be delivered 1:1 online. The psychosocial/motivational sessions will be delivered in small groups and be recorded and scored against criteria. All sessions will be recorded to reduce the risk of those delivering the intervention behaving differently when being recorded. From these sessions, a purposively selected subset (10%) of recordings will be analysed, covering all centres and across relevant intervention sessions. This will enable assessment of fidelity, and an understanding of which areas generated discussion, and what issues were discussed.

Fidelity of online exercise session dose will be monitored with total exercise duration and intensity compared to the exercise prescription. Guided home exercise will be monitored via exercise logs. The effectiveness of complex interventions can be influenced by the skill of those delivering the intervention, so in addition to fidelity, criteria for competence will be developed from the training manuals and assessed with a checklist. The control group individual practitioner appointment will also be recorded and scored against criteria.

## Sites

Sites will be set up using (but not restricted to) the following models:

1. Central Trial Hub (University Hospitals Coventry and Warwickshire): site will identify potential participants, screen for eligibility (as described in section 2.5), and send out invitation letters, Patient Information Leaflets (PIL) and Expression of Interest (EOI) forms. They will perform telephone screening calls, randomisation, consent and all outcome assessments. They will deliver all components of the active and control interventions i.e., 1:1 assessments and familiarisation sessions, and weekly catchups with participants. All live group exercise and behavioural support sessions will also be delivered from this site.
2. Participating Site: site will identify potential participants, screen for eligibility, send out invitation letters, PILs and EOI forms, and perform telephone screening calls, randomisation, consent and all outcome assessments. All other aspects of the trial will be delivered from the Central Trial Hub. These sites may also perform outcomes assessments for PIC sites.
3. PIC Site: site (or a clinically trained member of the SPHERe team) will identify potential participants, screen for eligibility and send out invitation letters, PILs and PIC EOI forms. Telephone screening and face to face assessments will be performed by staff at the nearest participating SPHERe NHS Trust with capacity, or the Central Trial Hub (University Hospitals Coventry and Warwickshire). This is dependent upon participants living within a reasonable travelling distance of the participating NHS Trust/Central Trial Hub. The staff at the NHS hospital trust performing telephone screening calls will obtain contact details for interested participants, identified through PIC sites.

## 2.11 Allocation Concealment

Participants’ treatment allocation is revealed at randomisation which should take place at the end of the baseline assessment. Treatment allocation should be concealed from the practitioners conducting the follow up assessments at four and 12 month time points. The follow up practitioners therefore need to be different staff members to those performing the baseline assessment. To maintain this allocation concealment, sites will divide staff members into two groups: 1) Those who conduct baseline assessments and at the Central Trial Hub deliver intervention and/or control 2) Those who conduct follow up assessments at four and 12 month time-points. Those in the former group will be those that are permitted to receive notification at the point of randomisation revealing allocation.

**NB for site staff:** Completed GP letters, S/AE forms, randomisation notification emails and signed exercise bike loan agreements have the potential to unblind allocation, so should be a kept in a concealed Investigator Site File. This site file should not be accessed by those performing follow up assessments.

At the four and 12 month assessments, participants will be asked to not reveal their allocation to the assessing practitioner. If allocation is revealed, the practitioner will record this on the follow-up form.

## 2.12 Site Staff Training

**Intervention practitioners:** Practitioners delivering the SPHERe intervention will be Clinical Exercise Physiologists or Physiotherapists with appropriate professional registration, relevant Continued Professional Development (CPD), and Good Clinical Practice (GCP) training.

The live exercise and support sessions will be delivered online from the Central Trial Hub – UHCW NHS Trust. This will also include assessment of ongoing exercise prescription and the individual assessments for both arms. There will be approximately 5 SPHERe practitioners (Clinical Exercise Physiologist/Physiotherapists) who will deliver these sessions as described (including the additional control/intervention elements for participants identified through UHCW NHS Trust and other participating sites where required). Additional SPHERe practitioners at UHCW may be identified during the course of the study. Practitioners at other participating NHS Trusts will perform telephone screening calls, randomisation, consent and all outcome assessments.

**SPHERe training:** All intervention practitioners will undergo one day of SPHERe training. This training will ensure an appropriate level of clinical knowledge and skills for exercise rehabilitation in PH. Training will be delivered by a health psychologist, to upskill practitioners on the psychosocial components of the intervention. Training and subsequently the trial, will be supported by a comprehensive practitioner intervention manual. Access to expertise and support will be maintained and monitored throughout the duration of the trial. Full training will be provided by the SPHERe research fellow and health psychologist for new staff, as needed.

**SPHERe practitioner manual:** This detailed manual will guide practitioners through each component of the intervention, graphically and with written instruction. It will also include general information about the trial, key components of GCP and contact details of the trial team. The content will reflect information delivered during the training for SPHERe intervention practitioners.

**Exercise intervention:** To enhance practitioners’ knowledge of exercise assessment and prescription in PH, ensuring intervention efficacy and safety, the manual will provide an overview of key evidence and exercise guidance. To provide a level of standardisation, parameters within which the exercise intervention should be delivered and progressed, will be detailed.

**Psychosocial and motivational intervention:** The manual will give a detailed description of each psychosocial topic, with hints and tips of questions to ask, and the aims of each session. The content will map onto the intervention participant manual (see section 2.9.1), allowing the practitioner to tailor the discussion.

## 2.13 Concomitant Medication

A full medication list will be recorded for each participant at trial entry and at 4 and 12 month time points on the Medications form.

## 2.14 End of Trial

The trial will end when all participants have completed their 12-month follow-up. As part of the process evaluation n=20 controls and n=20 intervention will be interviewed **after** their 4-month follow-up. Pilot participants will be interviewed before this timepoint.

The trial will be stopped prematurely if:

- Mandated by the Ethics Committee
- Recommended by the DMC
- Funding for the trial ceases.

The Research Ethics Committee will be notified in writing within 90 days when the trial has been concluded or within 15 days if terminated early.

# METHods and assessments

## Schedule of Data Collection

Table 1. Data collected at trial time points

| **Visit** | Phone/Clinic | 1 | 2 | 3 |
| --- | --- | --- | --- | --- |
| **Time-point** | Pre-consent | Baseline | 4 months  (+/- four weeks) | 12 months  (+/- four weeks) |
| Eligibility checks (on phone/in person) | **X** |  |  |  |
| Written/verbal information | **X** |  |  |  |
| Written informed consent* |  | **X** |  |  |
| Case report form |  | **X** | **X** | **X** |
| Incremental shuttle walk test* |  | **X** | **X** | **X** |
| CAMPHOR |  | **X** | **X** | **X** |
| EQ-5D |  | **X** | **X** | **X** |
| HADS |  | **X** | **X** | **X** |
| Generalised self-efficacy scale |  | **X** | **X** | **X** |
| Fatigue severity scale |  | **X** | **X** | **X** |
| WHO functional class |  | **X** | **X** | **X** |
| Time to clinical worsening |  |  | **X** | **X** |
| Medication use* |  | **X** | **X** | **X** |
| Health/social care resource use |  |  | **X** | **X** |
| All-cause mortality |  |  | **X** | **X** |
| Adverse events* |  | **X** | **X** | **X** |
| Semi-structured interviews** |  |  | **X** |  |
| Demographic data |  | **X** |  |  |

* information collected in person. All other outcome data will be collected remotely.

** interviews for pilot participants will be held after the 4 month follow-up timepoint.

## 3.2 Longer Term Follow-up Assessments (England and Wales only)

Consent will be sought from participants (recruited in England and Wales) for WCTU to keep their personal data and have access to their NHS data beyond the end of the funded trial. This will allow longer term postal follow-up to assess quality of life and to monitor deaths using NHS registry data.

## 3.3 Process Evaluation

*Semi-structured interviews with participants:* Interviews will be conducted by a qualitative Research Fellow from WCTU, in person or on the phone/over MS Teams as appropriate. Intervention and control participants will be interviewed to investigate their experiences, contextualise quantitative findings, and explore factors that helped or hindered participation (which could include COVID-19), thus informing interpretation and wider implementation. Interviews will take place after the 4-month follow-up outcome data collection, so that the interview itself does not introduce bias to the collection and reporting of the 4-month outcomes. During the internal pilot interviews will be conducted sooner than this following completion of the trial intervention. A purposive sample of approximately n=20 intervention and n=20 control participants will be interviewed to ensure a diverse range of perspectives are included. Participants from the pilot phase will be interviewed before the 12-month follow-up. The interviews will use a topic guide that will include participant response to the intervention (or control), what was difficult, what worked well, specific obstacles and enablers, what components were used/dropped/never used, and for the intervention arm, views on the individual 1:1 sessions, the remotely supervised exercise, the guided home exercise content, and the psychosocial, motivational, and education online group. Interviews will last about one hour, be digitally recorded, and piloted with up to five people from the internal pilot.

*Practitioner interviews****:*** At the end of the trial, a purposive sample of up to n=20 practitioners will be interviewed about their experiences of delivering the interventions/best usual care, what worked well, what helped, and what was challenging. These interviews will last up to one hour, be digitally recorded, and piloted with up to five practitioners.

# adverse event management

## Definitions

### Adverse Events (AE)

An Adverse Event (AE) is defined as any untoward medical occurrence involving a participant, which does not necessarily have a causal relationship with the intervention or trial.

**Expected AEs,** related to the exercise outcome assessments or the exercise intervention (supervised or SPHERe home programme), include ‘normal’ levels (for the individual) of:

- Breathlessness
- light headedness/dizziness
- muscle stiffness/soreness
- tiredness/fatigue
- exertional chest pain
- O_2_ desaturation.

Recording procedures will be the same for both trial groups. For the **intervention group,** **expected** AEs will be recorded in the participants’ notes (not on an AE form), for clinical purposes only. For the **usual care group** these will be recorded on the participants’ exercise outcome assessment notes (not on an AE form) for clinical purposes only.

**Unexpected AEs** related to the exercise outcome assessments or the exercise intervention for both the intervention and usual care groups, will be recorded on the appropriate CRF and returned routinely to WCTU.

AEs in the **intervention group** will be determined through patient report at each weekly online catch-up session and via a phone call to the participant the day following their appointment/outcome assessments. AEs in the **usual care group** will be determined via a phone call to the participant the day following their appointment/outcome assessments. This call is not routine and will be made by the SPHERe outcomes assessors.

### Serious Adverse Events (SAEs)

A substantial number of serious adverse events (SAE) are expected in this population. Over the four-month follow-up period, many people will be admitted to hospital, possibly on multiple occasions, and some deaths are expected. Hospital admissions and deaths are important outcomes for this trial. Admissions data will be collected from self-report, hospital and GP records, and deaths will be collected from hospital and GP records. These data will be presented to the TSC and DMC.

For SPHERe, an SAE will be an untoward medical occurrence that fulfils one or more of the following criteria:

- Results in death
- Is immediately life-threatening
- Requires hospitalisation or prolongation of existing hospitalisation
- Results in persistent or significant disability or incapacity
- Is a congenital anomaly or birth defect.
- Requires medical intervention to prevent one of the above, or is otherwise considered medically significant by the investigator (e.g. participant safety is jeopardised).

SAEs that may be expected as part of the interventions are pre-defined below and will be recorded on the Adverse Event/Non Reportable SAE form for routine return to WCTU SPHERe trial team.

The following SAEs are **expected** with PH and are therefore **not reportable** for this trial, but they will be **recorded** on the Adverse Event/Non Reportable SAE form:

- Hospitalisation for cardio/respiratory condition (including COVID-19) or related complication.
- PH disease progression: worsening exercise tolerance, atrial arrhythmias or decreasing oxygen saturations; up titration of pulmonary vasodilators; or development of heart failure or signs of fluid overload (including pleural/pericardial effusions, ascites).
- PH disease related death.
- Treatment, which was elective or pre-planned, for a pre-existing condition, not associated with any deterioration in condition.
- General care, not associated with any deterioration in condition.

**Reportable SAEs - intervention group:** SAEs directly related to exercise sessions or outcomes assessments are possible. In the intervention group, any event that occurs from the baseline assessment to the four-month follow-up, or within 24 hours of the four and 12 month outcome assessments, will be recorded and reviewed to determine if it is directly attributable to the intervention/assessment (i.e. a causal relationship to the intervention is at least possible), and investigated in line with Warwick SOPs. These will be captured by participant report at weekly catch-up sessions. Any event that occurs after the four-month follow-up assessment, or outside 24 hours of the four and 12 month outcome assessments, will not be reported for this trial.

If a participant has not attended two consecutive online intervention appointments, their status will be checked on local electronic clinical records by the local SPHERe clinical rehabilitation team (with participant consent). If their status is unclear, the SPHERe clinical rehabilitation team will attempt to make contact on the phone at least three times. If they remain uncontactable, the participant’s next of kin will be contacted. SAEs related to the four and 12 month outcomes assessments will be determined via a phone call to the participant, the following working day after their appointment.

**Reportable SAEs - usual care group**: any event occurring within 24 hours of the assessment appointments (baseline, four months and 12 months) will be recorded and reviewed to determine if it is directly attributable to the trial assessments; this will be determined via a phone call to the participant, the following working day after their appointment. Any SAEs occurring between the baseline phone call and the four month follow-up appointment will be determined by participant self-report at the four month outcome assessment.

All participants experiencing SAEs during the period up to the four-month follow-up assessment, or during the 24 hour periods after the four and 12 month outcome assessments, will be followed-up until resolution of the event. Any event that occurs after the four-month follow-up assessment, or outside 24 hours of the four and 12 month outcome assessments, will not be reported for this trial.

**Recording Adverse Events and Reporting Serious Adverse Events**

**Recording and reporting period**

**Intervention group:** All AEs and SAEs that occur during or within 24 hours of a SPHERe live online session or an on-demand exercise session (supervised or unsupervised) should be recorded; AEs will be recorded on the Adverse Event/Non Reportable SAE Form; Reportable SAEs will be recorded on the SAE Form; SAEs expected with PH will be recorded on the Adverse Event/Non Reportable SAE Form.

**Usual care group:** The usual care group will be asked about anything that might constitute a Serious Adverse Event at the time of their four month follow-up. It will not be possible to collect a comparison dataset for the usual care group within this period without contaminating the control intervention. This is a pragmatic study and the participants will not be contacted during the intervention period, unlike the intervention group. It is important not to contact the usual care group more than is necessary so as not to introduce bias. We anticipate a low risk of adverse events arising from best practice usual care i.e. an NHS website and a single session of advice.

## Reporting Related and Unexpected SAEs

All reportable SAEs will be reported on the SAE form, scanned and sent to WCTU QA team ([WCTUQA@warwick.ac.uk](mailto:WCTUQA@warwick.ac.uk)), within 24 hours of the investigator being made aware. The trial manager will liaise with the local PI to compile all the necessary information. WCTU is responsible for reporting any related and unexpected SAEs to the Sponsor and REC within required timelines. All SAEs will be recorded for inclusion in the annual reports to the REC. The CI, in consultation with the trial medical team, will review causality.

The causality of SAEs (i.e. relationship to trial intervention) will be assessed by the investigator(s) using the SAE form (Table 2).

Table 2. SAE causal relationship

| **Relationship**  **to trial medication** | **Description** |
| --- | --- |
| Unrelated | There is no evidence of any causal relationship |
| Unlikely to be related | There is little evidence to suggest there is a causal relationship (e.g. the event did not occur within a reasonable time after administration of the trial intervention). There is another reasonable explanation for the event (e.g. the patient’s clinical condition, other concomitant treatment). |
| Possible relationship | There is some evidence to suggest a causal relationship (e.g. because the event occurs within a reasonable time after administration of the trial intervention). However, the influence of other factors may have contributed to the event (e.g. the patient’s clinical condition, other concomitant treatments). |
| Probable relationship | There is evidence to suggest a causal relationship and the influence of other factors is unlikely. |
| Definitely related | There is clear evidence to suggest a causal relationship and other possible contributing factors can be ruled out. |

To establish causality, the following information should be collected for each SAE:

- full details in medical terms and case description
- event duration (start and end dates, if applicable)
- action taken
- outcome
- seriousness criteria
- causality (i.e. relatedness to intervention), in the opinion of the PI
- whether the event would be considered expected or unexpected.

SAEs that are deemed to be unexpected and possibly, probably or definitely related to the trial interventions or outcomes assessments, will be notified to the Research Ethics Committee (REC) and sponsor within 15 days. All such events will be reported to the TMG at their next meeting. All SAEs that occur between the date of randomisation and the end of the fourmonth follow-up, and within 24 hours following the 4 month and 12 month assessment, will be recorded on the relevant form

Any change of condition or other follow-up information should be communicated to the Sponsor as soon as it is available, ideally within 24 hours of the information becoming available. Events will be followed until resolution or a final outcome has been reached. A member of the PI’s trial team will be instructed to closely monitor each participant who experiences a SAE, until the outcome of the SAE has been determined.

**Annual reporting:** All related and unexpected SAEs will be recorded for inclusion in annual reports to the Research Ethics Committee.

## Responsibilities

Principal Investigator:

1. Checking for AEs when participants attend for treatment/follow-up.
2. Using clinical judgement in assigning seriousness, causality and expectedness.
3. Ensuring that all reportable SAEs are recorded and reported to Warwick within 24 hours of becoming aware of the event and providing further follow-up information as soon as available.
4. Ensuring that AEs and non-reportable SAEs are recorded and reported to Warwick in line with the requirements of the protocol.

Chief Investigator/delegate or independent clinical reviewer:

1. Clinical oversight of the safety of patients participating in the trial, including an ongoing review of the risk / benefit.
2. Using clinical judgement in assigning seriousness and causality of reportable SAEs.
3. Immediate review of all related and unexpected SAEs.
4. Review specific SAEs in accordance with the trial risk assessment and protocol as detailed in the Trial Monitoring Plan.
5. Production and submission of annual reports to the relevant REC.

Sponsor (oversight), duties delegated to WCTU:

1. Oversight of safety reporting process, review of cumulative safety data.
2. Assess the expectedness of reportable SAEs.
3. Reporting safety information to the CI, delegate or independent clinical reviewer for the ongoing assessment of the risk/benefit according to the Trial Monitoring Plan.
4. Reporting safety information to the independent oversight committees identified for the trial (DMC and TSC) according to the Trial Monitoring Plan.
5. Expedited reporting of related and unexpected SAEs to the REC within required timelines.
6. Notifying Investigators of related and unexpected SAEs that occur within the trial.

Trial Steering Committee:

In accordance with the Trial Terms of Reference and/or charter for the TSC, periodically reviewing safety data and liaising with the DMC regarding safety issues.

Data Monitoring Committee:

In accordance with the Trial Terms of Reference and/or charter for the DMC, periodically reviewing unblinded overall safety data to determine patterns and trends of events, or to identify safety issues, which would not be apparent on an individual case basis.

## Notification of Deaths

All deaths will be reported to the Sponsor irrespective of whether the death is related to disease progression, trial intervention, or an unrelated event.

## Reporting Urgent Safety Measures

If any urgent safety measures are taken, the CI/Sponsor shall immediately and, in any event, no later than 3 days from the date the measures are taken, give written notice to the relevant REC of the measures taken and the circumstances giving rise to those measures.

# Data management

Personal data collected during the trial will be handled and stored in accordance with the General Data Protection Regulation.

Personal identifying information will be sent to and stored both in paper form and electronically at WCTU. Participant and NOK details will be stored and accessed by staff at WCTU and UHCW via the online database to confirm initial eligibility; contact participants during the study; allow delivery of intervention and control procedure and to contact for qualitative interviews. Handling of personal data will be clearly documented in the participant information sheet and consent obtained.

SPHERe practitioners at the Central Hub (UHCW) will also keep paper records of participant contact details and medical health information for those randomised to the intervention. This is required for study delivery, to ensure participants are exercising at the appropriate level and to be used if a medical emergency occurs. These paper records will be stored securely in locked filing cabinets only accessible to study staff. These records will not be passed on to WCTU.

Disclosure of confidential information will only be considered if there is an issue which may jeopardise the safety of the participant or another person, according to WCTU SOPs (WCTU SOP 15 part 1) and the UK regulatory framework. There is no reason to expect this situation to occur in this trial more than any other.

## Data Collection and Management

The CRFs will be developed by the trial manager in consultation with the CI, statistician, health economist and other relevant members of the trial team to collect all required trial data. A suitably trained member of the research team at each site will complete the CRFs and enter the data onto the secure online trial database hosted by WCTU as outlined in the data management plan and in accordance with the WCTU SOPs. Paper questionnaires (Baseline, 4m and 12m) will be either be inputted at site or a copy returned to the WCTU.

Various methods will be used to chase missing data/unreturned questionnaires including post, phone, text and email (section 2.9.2), the procedures for managing this will be outlined in the data management plan and appropriate consent will be sought to contact participants. Data will still be collected for participants who discontinue or deviate from the intervention protocol, unless they withdraw their consent (section 2.9.2).

## Database

The database will be developed by the Programming Team at WCTU and all specifications (i.e. database variables, validation checks, screens) will be agreed between the programmer and appropriate trial staff including the trial statistician.

## Online Platform

An external online video platform (BEAMfeelgood) will be used for the SPHERe trial interventions. This platform will enable live streaming of intervention sessions and the hosting of on-demand, pre-recorded content. A Data Protection Impact Assessment has been completed and approved by UHCW NHS Trust in order to identify and minimise associated risks. The online platform is GDPR compliant and Organisation for Review of Care and Health Apps (ORCHA) accredited. Any data that is stored, including the participant’s email address, will be encrypted in accordance with NHS Digital guidance and storage will be NHS cloud compliant, conforming to ISO 9001/27001/27017/27018 standards and the G-Cloud (UK Government) standard.

Private groups will be created on the online platform and administrative access to these given only to UHCW/UoW approved staff. Admin users will authorise participant’s access to the private groups and participants will be asked for their consent to share utilisation metrics with the group admin. Participants may choose to use a nickname on the online platform to remain anonymous to other members.

This online video platform will collect and store data on participants attendance at classes, the amount of time participants have spent watching on demand videos and answers to any post exercise session online poll questions. This information will be stored against the participants name and email address on google data studio until the end of the study. The SPHERe team (WCTU and UHCW) will be given access to this data as required to monitor attendance, safety and for analysis of compliance.

## One-to-one Consultations/Weekly Catch-ups Platform

All one-to-one consultations and weekly catch-ups between the SPHERe practitioner and a trial participant will take place on an online video platform (MS Teams or similar) supported and approved by UHCW Trust. This will include the best practice usual care advice consultation in the control group and the individual assessment component of the SPHERe intervention.

## Data Storage

All essential documentation and trial records will be stored at WCTU in conformance with the applicable regulatory requirements and access to stored information (paper and electronic) will be restricted to authorised personnel. All data will be stored in a designated storage facility within the WCTU. Electronic data will be stored on password protected university computers.

## Data Access and Quality Assurance

Confidentiality will be strictly maintained and names or addresses of participants will not be disclosed to anyone other than the staff involved in running the trial. All electronic participant-identifiable information will be held on a secure, password-protected database accessible only to essential personnel. Paper forms with participant-identifiable information will be held in secure, locked filing cabinets within a restricted area of WCTU. Participants will be identified by a participant number only. Direct access to source data/documents will be available for trial-related monitoring or audit by UHCW or WCTU for internal audit or regulatory authorities. The PI must arrange for retention of trial records on site in accordance with GCP and local Trust’s policies.

Direct access to source data/documents will be required for trial-related monitoring. For quality assurance, the data and results will be statistically checked. A full data management plan will be produced by the trial manager and statistician to outline the data monitoring checks required.

## Data Shared with Third Parties

Requests for data sharing will be managed in accordance with University of Warwick/WCTU policy on data sharing. The datasets generated during and/or analysed during the current trial are/will be available upon request after publication of the main trial results. The publication of a trial protocol, trial results and trial data will be in line with the NIHR standard terms and will follow WCTU SOP 22: Publication & Dissemination.

## Archiving

Trial documentation and data will be archived for at least ten years after completion of the trial. Trial documentation and data held by recruiting NHS sites will be stored in line with their local trust policy.

# Statistical analysis

## Power and Sample Size

The primary outcome will be **distance walked measured using the ISWT at four months post-randomisation.** As there are no directly applicable ISWT data with which to calculate a sample size, or previously defined worthwhile effect sizes on ISWT for people with PH, to inform a sample size calculation, so 6MWT data have been used to inform the sample size estimation. The 6MWT distance, unlike the alternative approach of using a standardised mean difference, has the advantage that it is meaningful to our participants and grounded in clinical reality.

The baseline pooled 6MWT distance in current studies of exercise rehabilitation for PH is 414 meters (SD 91) [18]. Whilst a useful starting point, these data indicate a comparatively fit group of people with PH (younger, group 1 PH). Typically, people with PH, seen in the cardio-pulmonary rehabilitation service at UHCW, walk around 300m in the 6MWT. Conventionally, the minimally clinically important difference for PH studies is 30m on 6MWT or a standardised mean difference (SMD) of 0.33 [19]. Our patient partners suggest that a larger difference is needed to make this treatment worthwhile; it requires a substantial commitment from people debilitated with potentially life-limiting PH to attend the treatment sessions. Therefore, sample size is predicated on showing a mean difference of 45m in 6MWT distance. This equates to a standardised mean difference of 0.5; conventionally a moderate effect size.

Further support for choosing this effect size comes from the related area of COPD research where patients indicate that the smallest difference in walk distance they might perceive as worthwhile is 54m (31-71) on 6MWT [55]. Using the convention that an effect size of around half of the minimally important within person change can be taken as a moderate between group effect, suggests that 27m might be a relevant between group difference in 6MWT distance for our trial. A difference of 45m in 6MWT should be a worthwhile benefit to patients with PH.

It is unusual to use a secondary outcome to inform a sample size. However, it is preferable to using a primary outcome with substantial concerns regarding its measurement properties in the population of interest (i.e. 6MWT) [37]. However, as the data are not available to make a robust estimate based on ISWT, this approach is preferred, grounded in clinical reality, and strongly supported by our patient group/partners, rather than simply using a statistical convention based on standardised mean difference.

To achieve 90% power at 5% significance level, to show a difference in 6MWT distance of 45m, with a standard deviation of 90, data from 170 people are needed. Experience across multiple studies has been that the effects of clustering by group or practitioner are trivial. Nevertheless, allowance has been made for clustering effects by site in the intervention arm, using Moerbeek’s method [56] and an unbalanced randomisation. This allows calculation of the most efficient sample size for a trial with clustering in just one trial arm, and generates an unbalanced randomisation.

The unequal allocation (1.15:1 for intervention vs control) was determined based on the following assumptions: a mean cluster size of 12 at follow-up, an ICC of 0.03 and same group variance. The ICC is an overall estimation of the site and practitioner effect, leading to an estimated design effect of 1.33, although a negligible practitioner effect in this trial is anticipated. Accordingly, the group sample sizes were calculated separately, given the power of 90% and a significance level of 5%. A minimum 80% retention rate is expected at four months based on clinical experience of working with this patient group, and exercise rehabilitation complex intervention trial experience in similar population. Therefore, 246 participants (132 in intervention) will be recruited to allow for 20% loss to follow-up (whilst striving to keep this below 10%).

The primary aim is to show an overall effect size for all groups without considering participant mix. Based on published data for prevalence of PAH and CTEPH, however, most participants will have PH groups 2 or 3; i.e. secondary to cardiac or pulmonary disease. These are also the groups where there is the most pressing need for data to inform clinical management. Existing data support the use of exercise rehabilitation to improve QoL in cardiac and pulmonary disease without PH, i.e. the underlying conditions of groups 2 & 3 PH. Sufficient data will be collected to assess outcome in a pooled group of people with group 2 or 3 PH as a secondary analysis. Approximately 70% of the total sample size will be people with group 2 or 3 PH. To ensure power of 90% power, for this sub-group, the latter sample size of 246 will inflated to around **352 participants*.** This will be the total sample for the trial which will ensure sufficient power for the main analysis as well as the sub-group analysis. There is some uncertainty about the final sample size because of the need to include 246 people with types 2/3 in our overall population and an ambition to include a minimum of 20 people each from sub-groups 1, 4, & 5.

Representation of different sub-groups will be monitored during recruitment and, if necessary, steps taken to ensure each sub-group is represented appropriately.

*Sample size revised to 200 participants- see Section 6.1.1 for details.

### Revised Sample Size

To revise the sample size, we used observed parameters from 79 randomisations and 43 primary outcome follow-ups:

1. Number of patients in PH groups 2/3 = 35/79 (44%)
2. Intervention group size between 5 and 10
3. Observed ICC = 0.03
4. Allocation ratio = 1.04:1 (cluster size =5) and 1.10:1 (cluster size =10)
5. Effect size = 0.5
6. Lost to follow-up =2 4%
7. Correlation coefficient = 0.8.

To show our target difference, with this level of correlation, we need to recruit **85-90 participants with type 2/3 PH** (depending on the cluster size). This is the number of participants we would need to show a benefit in the key group of people with type 2/3 PH (specified in the original brief). However, the proportion of people with type 2/3 PH is smaller than anticipated at the time of study design. This may reflect the move to online intervention delivery during the COVID-19 pandemic, meaning fewer older people with type 2/3 PH are able/willing to access the intervention (issues with access/competence in use of technology). Currently, 44% of our recruited participants have type 2/3 PH compared to our pre-study estimate of 70%. Thus, we are aiming for an overall target of **around 200 participants** but with an intention to stop recruitment when we have 85-90 participants with type 2/3 PH.  The final overall recruitment may be substantially less than 200.

This change has been fully reviewed and approved by the TSC, DMC, Sponsor and Funder (NIHR).

## Statistical Analysis of Efficacy and Harms

### Statistics and Data Analysis

Unless otherwise stated, further details in relation to the planned analyses will be detailed in a statistical analysis plan (SAP), which will be agreed with the DMC. All data will be analysed and reported in accordance with the CONSORT statement. All primary analyses are planned to be on an intention to treat basis with secondary per protocol analysis.

### Planned Recruitment Rate

During a six month internal pilot, from four NHS Trusts, 60 (25-30 per arm) participants will be enrolled. Running seamlessly into the main trial, by the end of the pilot, the aim is to be recruiting 23 participants per month in total from all Trusts combined. This will continue for the remainder of recruitment.

### Statistical Analysis Plan

Treatment effects will be presented, with appropriate 95% confidence intervals, for both the unadjusted and adjusted analyses. Tests will be two-sided and considered to provide evidence for a significant difference if p-values are less than 0.05 (5% significance level). All analyses will be conducted as intention to treat unless otherwise specified.

### 6.2.3.1 Summary of Baseline Data and Flow of Participants

Baseline data will be summarised to check comparability between treatment arms, and screening data will be checked to highlight any characteristic differences between those individuals in the trial, those ineligible, and those eligible but withholding consent. A CONSORT chart illustrating participant flow throughout the trial will be produced. Standard statistical summaries will be presented for the primary outcome measure (ISWT) and all secondary outcome measures.

### 6.2.3.2 Primary Outcome Analysis

The main analyses will be for overall treatment effect regardless of PH diagnostic group. Data will be summarised and reported in accordance with CONSORT guidelines for RCTs, using intention-to-treat analyses [57]. Hierarchical linear regression models will be used to estimate the treatment effects (95% confidence intervals), adjusted for important patient-level covariates and centre effect. These will be defined in the final analysis plan. Estimation of, and adjustment for practitioner effects will be included. If there is negligible practitioner and centre effect, then the usual linear regression will be used for the analysis. Categorical data will be assessed in a similar way, using logistic regression models. The main analyses will all be intention to treat. Any control participants referred to cardio-pulmonary rehabilitation as part of their routine clinical care will be analysed according to their original randomisation.

We will assess the impact of compliance on outcomes using a CACE (Compliers average causal effect) analysis. For the intervention group, partial compliance will be defined as completion of the initial online assessment and familiarisation, and at least half of the supervised home exercise, and psychosocial/motivational sessions. Full compliance will be considered as attending at least 75% of the supervised home exercise, and psychosocial/motivational sessions. In addition we will aim to present probabilities for achieving the desired effect size in each of the groups using the magnitude based inference approach[58].

In a planned secondary analysis, the pooled effects for groups 2 & 3 will be presented (see below). Main outcomes will also be presented by diagnostic group (minimum 20 people contributing data) to inform decision makers and guidance developers interested in specific groups. To maximise data value, data from published trials (identified in an updated systematic review) assessing the same outcomes in RCTs of home-based interventions for specific PH group, will be included.

## Subgroup Analyses

Pre-specified sub-group analyses will examine the interaction of treatment assignment with the groupings of PH. Analysis will be conducted using formal tests of interaction. This trial is not powered to identify interactions. Thus, whilst pre-specified, these analyse should be considered as no more than exploratory. We will, however, present the effect size for pooled groups 2&3 as a separate analysis.

## Subject Population

The primary analysis and any secondary analyses will be applied to an all-randomised population on an intention-to-treat basis. That is, any subject randomised into the trial, regardless of whether they received trial intervention and regardless of protocol deviations, unless specified above.

## 6.5 Procedure(s) to Account for Missing or Spurious Data

Whilst every effort will be made to ensure compliance and data collection, it is inevitable that some data will be missing and likely that cross-overs will occur (i.e. exercise sessions not attended or participant requests for treatment). Careful monitoring of missingness and crossovers will be conducted. If judged appropriate, Multiple Imputation (MI) will be used to account for missing data, with all necessary assumptions reported. If large numbers of treatment cross-overs are observed, Complier-Average Causal Effect (CACE) models will be used. Similar to Per Protocol (PP) methods, CACE models evaluate the average effect of the intervention in participants who comply with their allocated treatment. This preserves randomisation groups and eliminates introducing any potential confounders introduced by PP analysis.

Some data may not be available due to voluntary withdrawal of participants, lack of completion of individual data items or general loss to follow-up. Where possible the reasons for data ‘missingness’ will be ascertained and reported. The nature and pattern of the missingness will be carefully considered, including whether data can be treated as missing completely at random. If judged appropriate, missing data will be imputed using the multiple imputation facilities available in statistical analysis software.

If imputation is undertaken, the resulting imputed datasets will be analysed, together with appropriate sensitivity analyses. Any imputation methods used for scores and other derived variable will be carefully considered and justified. Reasons for ineligibility, non-compliance, withdrawal or other protocol violations will be stated, and any patterns summarised. More formal analysis, for example using logistic regression with ‘protocol violation’ as a response, may also be appropriate and aid interpretation.

## Qualitative Data Analysis

The semi-structured interviews with up to 20 intervention group, 20 control and 20 practitioners’ will be digitally recorded, subject to the permission of each participant/practitioner, pseudo-anonymised, and transcribed verbatim. Data will be analysed using the Framework method [59], broadly as follows:

- Data familiarisation: reading of complete interview transcripts, listening to original recordings and use of field notes;
- Identifying a thematic framework: key issues, concepts and themes are identified and an index of codes developed;
- Indexing: whereby the index generated through identification of the thematic framework is applied to all data;
- Charting: a summary of each passage of text is transferred into a chart to allow more overall and abstract consideration of index codes across the data set and by each individual;
- Mapping and interpretation: understanding the meaning of key themes, dimensions and broad overall picture of the data and identifying and understanding the typical associations between themes and dimensions.

The charting process provides an opportunity to code data from numerous perspectives. The computer package NVivo 12 will be used to organise the analysis.

The findings of the qualitative work will be reported as a separate chapter in the final report but will also be incorporated in the discussion to bring together a synthesis of all the results, thus helping to explore and explain the overall impact ‘value’ of the interventions. Quantitative and qualitative data will be integrated using a mixed methods matrix’ where quantitative responses can be compared to interview data and recorded on a matrix [60]. This is particularly useful to reveal gaps between quantitative and qualitative insights.

From the intervention delivery recordings (initial practitioner assessment, and exercise familiarisation session, the weekly 1:1 troubleshooting session, the remotely supervised exercise, and the psychosocial/motivational group sessions) and control (1:1 session) recordings, a purposively selected subset (10%) of recordings will be analysed, with a checklist to assess fidelity and using the qualitative approach detailed above to help understand which areas generated discussion and what issues were discussed. Intervention fidelity will be assessed using the tenets highlighted by Mars et al. [60].

## 6.7 Health Economic Evaluation

A prospective economic evaluation, informed by the NICE Reference Case, will be described within a Health Economics Analysis plan (HEAP), to be set prior to any analysis.

The primary perspective will include NHS and Personal Social Services (PSS) costs. However, patient direct and indirect costs will also be included in a secondary broader societal perspective. Resource use collection will be tested in the internal pilot phase. Primary care and referral events will be captured both from health records and from participants, using a triangulation and adjudication approach to promote robust estimates of resource use. Participants will also report PSS and personal direct and indirect costs. Personal Social Services Research Unit (PSSRU) and national hospital reference costs will be used as principal unit cost sources. Patient level costs will be estimated by summing resources, costed using unit costs. Intervention costing will reflect the structure within which care is being given and will, by necessity, balance precision with practicality.

EQ-5D-5L responses will be used to generate quality-adjusted life years (QALYs) using the UK value set recommended by the EuroQol group [61]. These health state values will be used to estimate QALYs at the patient level, over one year, using the trapezoidal rule. Within its position statement, NICE supports continued use of the EQ-5D-5L descriptive system to collect QoL data within prospective clinical studies. Should NICE consider the SPHERe intervention as part of its future guidelines, trial-based EQ-5D-5L values will be mapped to 3L, if required. The EQ-5D-5L will be used as the overall HR-QoL outcome due to specific concerns about the sensitivity to change of other measures such as the SF-36, in this population. The EQ-5D-5L is likely to be more responsive to change than the 3L, and hence is preferred as a clinical outcome. Significant adverse events will be captured summatively in the QoL estimation.

Bivariate regression of costs and QALYs (with bootstrapping of models) will generate incremental cost per QALY estimates and credible intervals, cost-effectiveness acceptability curves, and value-of-information analysis. With regard to normality, invoking the central limit theorem avoids the problems that non-Gaussian link functions generate for the analysis. However, if distributions are very unusual, cost and QALYs will be conflated in a net benefit analysis evaluated at different thresholds of willingness to pay, reducing the analysis to a univariate regression problem.

Mechanisms of missingness of data will be explored following best practice, and (as appropriate) imputation sets will be used to avoid the potential bias of complete case analysis. The imputation model will use fully conditional (MCMC) methods (multiple imputation by chained equations). Predictive mean matching, drawing from the five nearest neighbours (knn=5), will be used to enhance the plausibility and robustness of imputed values. Each draw will be analysed independently using bivariate regression and the estimates obtained will be pooled to generate mean and variance estimates of costs and QALYs using Rubin’s rule – managing within and between variances for imputed samples. To minimise the information loss of finite imputation sampling, 20 draws will be taken. The distribution of imputed and observed values will be compared visually and statistically to establish the consequences of estimation.

The time horizon for costs and outcomes will be 12 months for the within trial analysis. If incremental costs and benefits are not convergent within the trial duration, a long-term decision analytical model will be developed, partially informed by longer-term mortality follow-up.

# Trial organisation and oversight

## Sponsor and Governance Arrangements

UHCW NHS Trust will sponsor the trial. The day-to-day running of the trial will be managed by WCTU according to Warwick SOPs, with UHCW SOPs used for contracting.

## Ethical Approval

All ethical approvals will be sought using the Integrated Research Application System. The trial will be conducted in accordance with relevant regulations and guidelines. Before enrolling people into the trial, each trial site must ensure that the local conduct of the trial has the agreement of the relevant NHS Trust Research & Development (R&D) department. Sites will not be permitted to enrol people into the trial until written confirmation of R&D agreement is received by the co-ordinating team. Substantial protocol amendments (e.g. changes to eligibility criteria, outcomes, analyses) will be communicated by the trial team to relevant parties i.e. investigators, RECs, participants, NHS Trusts and trial registries.

Annual reports will be submitted to the REC within 30 days of the anniversary date on which the favourable opinion was given, and annually until the trial is declared ended. The REC and sponsor will be notified of the end of the trial (whether the trial ends at the planned time or prematurely). The CI will submit a final report to the required authorities with the results, including any publications, within one year ending the trial.

## Trial Registration

The trial is registered with the International Standard Randomised Controlled Trial Number (ISRCTN) Register: ISRCTN 10608766.

## Notification of Serious Breaches to GCP and/or Trial Protocol

A “serious breach” is a breach which is likely to effect, to a significant degree:

- 1. the safety or physical or mental integrity of the subjects of the trial;
  2. the scientific value of the trial.

If a serious breach occurs, the sponsor will be notified immediately of any case where the above definition applies during the trial conduct phase.

## Indemnity

NHS indemnity covers NHS staff, medical academic staff with honorary contracts, and those conducting the trial. NHS bodies carry this risk themselves or spread it through the Clinical Negligence Scheme for Trusts, which provides unlimited cover for this risk. The University of Warwick provides indemnity for any harm caused to participants by the design of the research protocol.

## Administration

The trial management team will be based at WCTU, University of Warwick.

## Trial Management Group (TMG)

The Trial Management Group, consisting of the project staff and co-investigators involved in the day-to-day running of the trial, will meet regularly throughout the project. Significant issues arising from management meetings will be referred to the TSC or Investigators, as appropriate.

## Trial Steering Committee (TSC)

The trial will be guided by a group of respected and experienced personnel and trialists as well as at least one ‘lay’ representative. The TSC will have an independent Chairperson. Face to face meetings will be held at regular intervals determined by need but not less than once a year. Routine business is conducted by email, post or teleconferencing.

The Steering Committee, in the development of this protocol and throughout the trial will take responsibility for:

- - Major decisions such as a need to change the protocol for any reason
  - Monitoring and supervising the progress of the trial
  - Reviewing relevant information from other sources
  - Considering recommendations from the DMC
  - Informing and advising on all aspects of the trial.

The membership of the TSC will be approved and appointed by the NIHR. The full remit and responsibilities of the TSC will be documented in the Committee Charter which will be signed by all members.

## Data Monitoring Committee (DMC)

The DMC will consist of independent experts with relevant clinical research, and statistical experience. The DMC meeting frequency will be guided by the DMC chair, but will be suggested to be three months into the recruitment phase and regularly thereafter, as directed by the DMC chair. Confidential reports containing recruitment, protocol compliance, safety data and interim assessments of outcomes will be reviewed by the DMC. The DMC will advise the TSC as to whether there is evidence or reason why the trial should be amended or terminated. The membership of the DMC will be approved and appointed by the NIHR.

DMC meetings may also be attended by the CI and Trial Manager (for non-confidential parts of the meeting) and the trial statistician. The full remit and responsibilities of the DMC will be documented in the Committee Charter which will be signed by all members.

## Essential Documentation

A Trial Master File will be set up according to WCTU SOP 11 and held securely at the coordinating centre. The coordinating centre will provide Investigator Site Files to all recruiting centres involved in the trial.

## Financial Support

The trial has been funded by a grant from the NIHR Health Technology Assessment (HTA) programme further to a commissioned call: HTA: 17/129/02.

# Monitoring, AUDIT AND INSPECTION

The trial will be monitored by the Research and Development Department at UHCW (as representatives of the lead Sponsor) and by the Quality Assurance team at WCTU (as representatives of the trial coordinating centre and academic lead) to ensure that the trial is being conducted as per protocol, adhering to Research Governance and GCP. The approach to, and extent of, monitoring will be specified in a trial monitoring plan determined by the risk assessment undertaken prior to the start of the trial.

A Trial Monitoring Plan will be developed and agreed by the Trial Management Group (TMG) based on the trial risk assessment, including on site monitoring if applicable. Processes to be considered in the monitoring plan will include participant enrolment, consent, eligibility, and allocation to trial groups; adherence to trial interventions and policies to protect participants, including reporting of harm and completeness, accuracy, and timeliness of data collection. The plan will be available from the trial coordination centre and will also be lodged with the Sponsor. Whilst the monitors work in the same institution as the CI and trial team (WCTU), they will act independently in this role.

Sites persistently late in reporting SAEs, receipt of multiple late/poorly completed CRFs, or evidence from CRFs that the trial protocols and procedures are not being adhered to (as assessed by the CI or the TMG) may be considered triggers for on-site monitoring visits. The Sponsor will ensure investigator(s) and/or institutions will permit trial-related monitoring, audits and REC review, providing direct access to source data/documents as required. Monitoring will be performed by exploring the trial dataset or performing central monitoring procedures and/or site visits, as defined in the trial monitoring plan. Recruitment sites are obliged to assist the sponsor in monitoring the trial. These may include hosting site visits, providing information for remote monitoring, or putting procedures in place to monitor the trial internally.

# Patient and Public InvolvEment (PPI)

Much of the SPHERe intervention was developed during the application process; the intervention was co-produced/developed/refined between January to June 2018, with patient and public involvement at every stage. Intervention components were modelled on existing clinical practice at NHS exercise rehabilitation centres, for people with severe COPD/CHF/ACHD/CHD (with co-existing PH). A three-stage process was followed as per MRC guidance: 1) systematic literature review; 2) expert opinion, stakeholder engagement and consensus meetings; 3) intervention piloting, acceptability and refinement.

Lay co-applicants were fully integrated into the development of this trial, taking an active role in refining intervention components and reviewing the application. They will sit on the trial management group (TMG), initially meeting monthly and subsequently quarterly, and will have a pivotal role in steering the conduct of the trial. They reviewed the ethics application to ensure that trial documentation e.g. participant information leaflet, was user appropriate. They will be given the opportunity to engage in trial publicity and the dissemination of findings through appropriate channels i.e. social media, lay conferences, public engagement events, service provider events, newsletter articles. A role description and terms of reference for lay co-applicants has been produced in collaboration with our lay partners and the UHCW Patient and Public Research Advisory Group (PRAG). This will ensure that both parties understand the nature and extent of the collaboration, and their expectations of each other.

Lay co-apps and partners will be supported by the CI, trial coordination team, and through the peer support of lay partners on existing clinical trials. Comprehensive training and support will be provided by UHCW NHS Trust R&D department with regular lay seminars, group training and social events through the PRAG, with governance from PALS. All activity will be appropriately reimbursed at INVOLVE rates, for which there is adequate provision in the grant application. Lay partners will also benefit from training and support from UNTRAP (Universities/User Teaching and Research Action Partnership), an active organisation through which local communities engage in research and teaching in health and social care, at the University of Warwick.

# Dissemination and publication

Results of the trial will be prepared by the research team and lay partners and submitted to the Funder as a final report. Findings will be submitted to peer-reviewed journals and disseminated to the medical and exercise rehabilitation communities. Papers will be published in open-access journals describing the development of the SPHERe intervention, the trial protocol, and results and data, in accordance with recommended guidance for transparent reporting, the Consolidated Standards of Reporting Trials (CONSORT) guidelines ([www.consort-statement.org)](http://www.consort-statement.org)), the NIHR standard terms, and WCTU SOP 22: Publication & Dissemination. Abstracts will be submitted to national and international conferences e.g. British Thoracic Society, British Cardiology Society, European Respiratory Society, American College of Cardiology.

The SPHERe intervention will be fully manualised and available for public access once the trial has been completed. If appropriate, a practitioner training programme will be developed to support the implementation of SPHERe.

Lay co-apps and partners will assist with dissemination of trial results to participants and the wider public. A lay summary will be produced for participants and the hospitals/centres involved. Results will be publicised via the trial website and social media e.g. Twitter. WCTU, with the lead clinical centre (UHCW) and lay partners, will jointly lead on strategies for knowledge dissemination and engagement within the NHS and wider public. All organisations will work together to ensure that clinically important findings are disseminated as widely as possible and, by working collaboratively, facilitate the adoption of such outcomes within the NHS to enhance patient care. Towards the end of the trial, a joint investigator and participant event will be hosted to release and promote key trial findings. Commercial outputs are not expected from this publicly funded trial, but intervention materials will be copyrighted as per institutional practice.

Work will be undertaken with national governing bodies (BACPR, BTS), charities (PHA-UK, BHF, BLF) and service audit providers (National Audit of Cardiac Rehabilitation [NACR], National Asthma and COPD Audit Programme [NACAP], NHS Digital PH audit), to promote the inclusion of people with PH in cardio-pulmonary rehabilitation programmes.

HRA guidance on information for participants at the end of a trial will be followed:

[*https://www.hra.nhs.uk/about-us/consultations/closed-consultations/guidance-participant-information-end-study-consultation/*](https://www.hra.nhs.uk/about-us/consultations/closed-consultations/guidance-participant-information-end-study-consultation/)*.*

# References

1. Kiely, D.G., et al., *Pulmonary hypertension: diagnosis and management.* BMJ, 2013. **346**: p. f2028.

2. Howard, L.S., *Prognostic factors in pulmonary arterial hypertension: assessing the course of the disease.* Eur Respir Rev, 2011. **20**(122): p. 236-42.

3. Babu, A.S., R. Arena, and N.R. Morris, *Evidence on Exercise Training in Pulmonary Hypertension.* Adv Exp Med Biol, 2017. **1000**: p. 153-172.

4. Arena, R., et al., *Exercise Training in Group 2 Pulmonary Hypertension: Which Intensity and What Modality.* Prog Cardiovasc Dis, 2016. **59**(1): p. 87-94.

5. Tran, D.L., et al., *Pathophysiology of exercise intolerance in pulmonary arterial hypertension.* Respirology, 2018. **23**(2): p. 148-159.

6. Guazzi, M. and R. Naeije, *Pulmonary Hypertension in Heart Failure: Pathophysiology, Pathobiology, and Emerging Clinical Perspectives.* J Am Coll Cardiol, 2017. **69**(13): p. 1718-1734.

7. Simonneau, G., et al., *Updated clinical classification of pulmonary hypertension.* J Am Coll Cardiol, 2013. **62**(25 Suppl): p. D34-41.

8. Vizza, C.D., et al., *Sildenafil dosed concomitantly with bosentan for adult pulmonary arterial hypertension in a randomized controlled trial.* BMC Cardiovasc Disord, 2017. **17**(1): p. 239.

9. Kim, N.H., *Group 4 Pulmonary Hypertension: Chronic Thromboembolic Pulmonary Hypertension: Epidemiology, Pathophysiology, and Treatment.* Cardiol Clin, 2016. **34**(3): p. 435-41.

10. Thenappan, T., et al., *Pulmonary arterial hypertension: pathogenesis and clinical management.* BMJ, 2018. **360**: p. j5492.

11. Fein, D.G., A.N. Zaidi, and R. Sulica, *Pulmonary Hypertension Due to Common Respiratory Conditions: Classification, Evaluation and Management Strategies.* J Clin Med, 2016. **5**(9).

12. NICE, *Quality standard (QS9) Heart failure in adults. Programme of cardiac Rehabilitation.* [*https://www.nice.org.uk/guidance/qs9/chapter/Quality-statement-6-Programme-of-cardiac-rehabilitation*](https://www.nice.org.uk/guidance/qs9/chapter/Quality-statement-6-Programme-of-cardiac-rehabilitation). 2016.

13. Bolton, C.E., et al., *British Thoracic Society guideline on pulmonary rehabilitation in adults.* Thorax, 2013. **68 Suppl 2**: p. ii1-30.

14. McCarthy, B., et al., *Pulmonary rehabilitation for chronic obstructive pulmonary disease.* Cochrane Database Syst Rev, 2015(2): p. CD003793.

15. Anderson, L. and R.S. Taylor, *Cardiac rehabilitation for people with heart disease: an overview of Cochrane systematic reviews.* Cochrane Database Syst Rev, 2014(12): p. CD011273.

16. Alison, J.A., et al., *Australian and New Zealand Pulmonary Rehabilitation Guidelines.* Respirology, 2017. **22**(4): p. 800-819.

17. Humbert, M., et al., *2022 ESC/ERS Guidelines for the diagnosis and treatment of pulmonary hypertension: Developed by the task force for the diagnosis and treatment of pulmonary hypertension of the European Society of Cardiology (ESC) and the European Respiratory Society (ERS). Endorsed by the International Society for Heart and Lung Transplantation (ISHLT) and the European Reference Network on rare respiratory diseases (ERN-LUNG).* Eur Heart J, 2022. **ehac237**.

18. Morris, N.R., F.D. Kermeen, and A.E. Holland, *Exercise-based rehabilitation programmes for pulmonary hypertension.* Cochrane Database Syst Rev, 2017. **1**: p. CD011285.

19. Holland, A.E., et al., *An official European Respiratory Society/American Thoracic Society technical standard: field walking tests in chronic respiratory disease.* Eur Respir J, 2014. **44**(6): p. 1428-46.

20. Gonzalez-Saiz, L., et al., *Benefits of skeletal-muscle exercise training in pulmonary arterial hypertension: The WHOLEi+12 trial.* Int J Cardiol, 2017. **231**: p. 277-283.

21. Morris, N.R., et al., *Study protocol for a randomised controlled trial of exercise training in pulmonary hypertension (ExTra_PH).* BMC Pulm Med, 2018. **18**(1): p. 40.

22. Chia, K.S., et al., *Randomised controlled trial examining the effect of an outpatient exercise training programme on haemodynamics and cardiac MR parameters of right ventricular function in patients with pulmonary arterial hypertension: the ExPAH study protocol.* BMJ Open, 2017. **7**(2): p. e014037.

23. Grunig, E., et al., *Safety and efficacy of exercise training in various forms of pulmonary hypertension.* Eur Respir J, 2012. **40**(1): p. 84-92.

24. Mereles, D., et al., *Exercise and respiratory training improve exercise capacity and quality of life in patients with severe chronic pulmonary hypertension.* Circulation, 2006. **114**(14): p. 1482-9.

25. McGregor, G., et al., *Exercise rehabilitation programmes for pulmonary hypertension – a systematic review of intervention components and reporting quality.* BMJ Open Sports and Exercise Medicine, 2018. **(in press)**.

26. Ehlken, N., et al., *Exercise training improves peak oxygen consumption and haemodynamics in patients with severe pulmonary arterial hypertension and inoperable chronic thrombo-embolic pulmonary hypertension: a prospective, randomized, controlled trial.* Eur Heart J, 2016. **37**(1): p. 35-44.

27. Nici, L., et al., *American Thoracic Society/European Respiratory Society statement on pulmonary rehabilitation.* Am J Respir Crit Care Med, 2006. **173**(12): p. 1390-413.

28. Harzheim, D., et al., *Anxiety and depression disorders in patients with pulmonary arterial hypertension and chronic thromboembolic pulmonary hypertension.* Respir Res, 2013. **14**: p. 104.

29. Pfeuffer, E., et al., *Anxiety, Depression, and Health-Related QOL in Patients Diagnosed with PAH or CTEPH.* Lung, 2017. **195**(6): p. 759-768.

30. Greaves, C.J., et al., *Optimising self-care support for people with heart failure and their caregivers: development of the Rehabilitation Enablement in Chronic Heart Failure (REACH-HF) intervention using intervention mapping.* Pilot and Feasibility Studies, 2016. **2**(1): p. 37.

31. Dalal, H.M., et al., *The effects and costs of home-based rehabilitation for heart failure with reduced ejection fraction: The REACH-HF multicentre randomized controlled trial.* European Journal of Preventive Cardiology, 2018. **26**(3): p. 262-272.

32. Devi, R., J. Powell, and S. Singh, *A web-based program improves physical activity outcomes in a primary care angina population: randomized controlled trial.* J Med Internet Res, 2014. **16**(9): p. e186.

33. Houchen-Wolloff, L., et al., *Web-based cardiac &lt;strong&gt;RE&lt;/strong&gt;habilitatio&lt;strong&gt;N&lt;/strong&gt; alternative for those declining or dropping out of conventional rehabilitation: results of the WREN feasibility randomised controlled trial.* Open Heart, 2018. **5**(2): p. e000860.

34. Holland, A.E., et al., *Home-based rehabilitation for COPD using minimal resources: a randomised, controlled equivalence trial.* Thorax, 2017. **72**(1): p. 57.

35. Horton, E.J., et al., *Comparison of a structured home-based rehabilitation programme with conventional supervised pulmonary rehabilitation: a randomised non-inferiority trial.* Thorax, 2018. **73**(1): p. 29.

36. Moher, D., K.F. Schulz, and D.G. Altman, *The CONSORT statement: revised recommendations for improving the quality of reports of parallel-group randomised trials.* Lancet, 2001. **357**(9263): p. 1191-4.

37. Billings, C.G., et al., *Incremental shuttle walk test distance and autonomic dysfunction predict survival in pulmonary arterial hypertension.* J Heart Lung Transplant, 2017. **36**(8): p. 871-879.

38. McKenna, S.P., et al., *The Cambridge Pulmonary Hypertension Outcome Review (CAMPHOR): a measure of health-related quality of life and quality of life for patients with pulmonary hypertension.* Qual Life Res, 2006. **15**(1): p. 103-15.

39. Herdman, M., et al., *Development and preliminary testing of the new five-level version of EQ-5D (EQ-5D-5L).* Qual Life Res, 2011. **20**(10): p. 1727-36.

40. Somaini, G., et al., *Prevalence of Anxiety and Depression in Pulmonary Hypertension and Changes during Therapy.* Respiration, 2016. **91**(5): p. 359-66.

41. Krupp, L.B., et al., *The fatigue severity scale. Application to patients with multiple sclerosis and systemic lupus erythematosus.* Arch Neurol, 1989. **46**(10): p. 1121-3.

42. Yorke, J., et al., *Symptom severity and its effect on health-related quality of life over time in patients with pulmonary hypertension: a multisite longitudinal cohort study.* BMJ Open Respir Res, 2018. **5**(1): p. e000263.

43. McLaughlin, V.V. and M.D. McGoon, *Pulmonary arterial hypertension.* Circulation, 2006. **114**(13): p. 1417-31.

44. Frost, A.E., et al., *Evaluation of the predictive value of a clinical worsening definition using 2-year outcomes in patients with pulmonary arterial hypertension: a REVEAL Registry analysis.* Chest, 2013. **144**(5): p. 1521-1529.

45. ACPICR, *Standards for physical activity and exercise in the cardiac population 2015.* Available at: <http://acpicr.com>.

46. ACSM, *Guidelines for exercise testing and prescription*. 10th ed. 2017, Riverwoods, IL: Lippincott Williams & Wilkins.

47. Whaley, M., et al., *American College of Sports Medicine’s guide to clinical exercise testing and prescription.* Vol. 9. 2013, Philadelphia: Lippincott Williams & Wilkins; 2013.

48. Buckley, J.P., et al., *BACPR scientific statement: British standards and core components for cardiovascular disease prevention and rehabilitation.* Heart, 2013. **99**(15): p. 1069-71.

49. Borg, G., *Borg's Perceived Exertion and Pain Scales*. 1998, Champaign, IL: Human Kinetics.

50. Forman, D.E., et al., *Prioritizing Functional Capacity as a Principal End Point for Therapies Oriented to Older Adults With Cardiovascular Disease: A Scientific Statement for Healthcare Professionals From the American Heart Association.* Circulation, 2017. **135**(16): p. e894-e918.

51. van der Wardt, V., et al., *Adherence support strategies for exercise interventions in people with mild cognitive impairment and dementia: A systematic review.* Prev Med Rep, 2017. **7**: p. 38-45.

52. Falck, R.S., et al., *How much will older adults exercise? A feasibility study of aerobic training combined with resistance training.* Pilot Feasibility Stud, 2017. **3**: p. 2.

53. Bandura, A., *Self-efficacy: toward a unifying theory of behavioral change.* Psychol Rev, 1977. **84**(2): p. 191-215.

54. Michie, S., M.M. van Stralen, and R. West, *The behaviour change wheel: a new method for characterising and designing behaviour change interventions.* Implement Sci, 2011. **6**: p. 42.

55. Redelmeier, D.A., et al., *Interpreting small differences in functional status: the Six Minute Walk test in chronic lung disease patients.* Am J Respir Crit Care Med, 1997. **155**(4): p. 1278-82.

56. Moerbeek, M. and W.K. Wong, *Sample size formulae for trials comparing group and individual treatments in a multilevel model.* Stat Med, 2008. **27**(15): p. 2850-64.

57. Schulz, K.F., D.G. Altman, and D. Moher, *CONSORT 2010 statement: updated guidelines for reporting parallel group randomised trials.* Bmj, 2010. **340**: p. c332.

58. Mengersen, K.L., et al., *Bayesian Estimation of Small Effects in Exercise and Sports Science.* PLoS One, 2016. **11**(4): p. e0147311.

59. Gale, N.K., et al., *Using the framework method for the analysis of qualitative data in multi-disciplinary health research.* BMC Med Res Methodol, 2013. **13**: p. 117.

60. Mars, T., et al., *Fidelity in complex behaviour change interventions: a standardised approach to evaluate intervention integrity.* BMJ Open, 2013. **3**(11): p. e003555.

61. Devlin N, S.K., Feng Y, Mulhern B, van Hout B. , *Valuing Health-Related Quality of Life: An EQ-5D-5L Value Set for England.* , O.o.H.E.R.P. 16/01., Editor. 2016.
