# Supplementary material for: Protocol update for a multi-centre randomised controlled trial of exercise rehabilitation for people with pulmonary hypertension: the SPHERe trial
Source: Trials. 2024 Jul 20;25:495. doi: 10.1186/s13063-024-08341-0 (PMC11264996; doi:10.1186/s13063-024-08341-0)
Supplement: Supplementary file 1 — Supplementary Material 1. [file 13063_2024_8341_MOESM1_ESM.zip › SPHERe Statistical and Health Economics Analysis Plan_v1.1_ESM.docx]

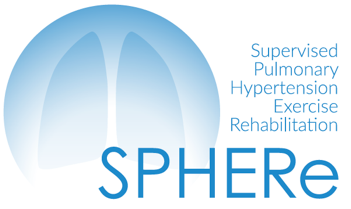


**Statistical and Health Economics Analysis Plan (SHEAP)**

Supervised Pulmonary Hypertension Exercise REhabilitation (SPHERe): a multi-centre randomised controlled trial

**Version 1.1**

**Date: 1 May 2024**

Protocol version: 8.0, 01 November 2022

IRAS No: 261218

REC and Approval date: West Midlands- Coventry & Warwickshire REC (13 Jul 2019)

REC ref: 19/WM/0155

ISRCTN: 10608766

| **Version** | **Date** | **Edited by** | **Comments/Justification** | **Timing in relation to unblinded interim monitoring** | **Timing in relation to unblinding of Trial Statisticians** |
| --- | --- | --- | --- | --- | --- |
| 0.1 | 16/03/2022 | MR | First draft. | Prior | Prior |
| 0.2 | 08/09/2022 | MR | Second draft | Prior | Prior |
| 0.3 | 16/11/2022 | MR | Third draft | Prior | Prior |
| 0.4 | 12/07/2023 | MR | Fourth draft | Prior | Prior |
| 0.5 | 31/08/2023 | MR | Fifth draft | Prior | Prior |
| 1.0 | 01/11/2023 | MR | Sixth draft | Prior | Prior |

SHEAP amendments

| Version | Date | Summary of changes | Location |
| --- | --- | --- | --- |
| 1.1 | 01/05/2024 | A further sensitivity analysis has been added with the imputation analysis under additional analyses section | Page 20 (section 4.6) |
| 1.1 | 01/05/2024 | Include a table for average age for gender at baseline | Page 38 (section 10) |

APPROVAL

| Lead Trial Statistician | Name: Prof Ranjit Lall, Warwick Clinical Trials Unit | |
| --- | --- | --- |
|  | Signature: 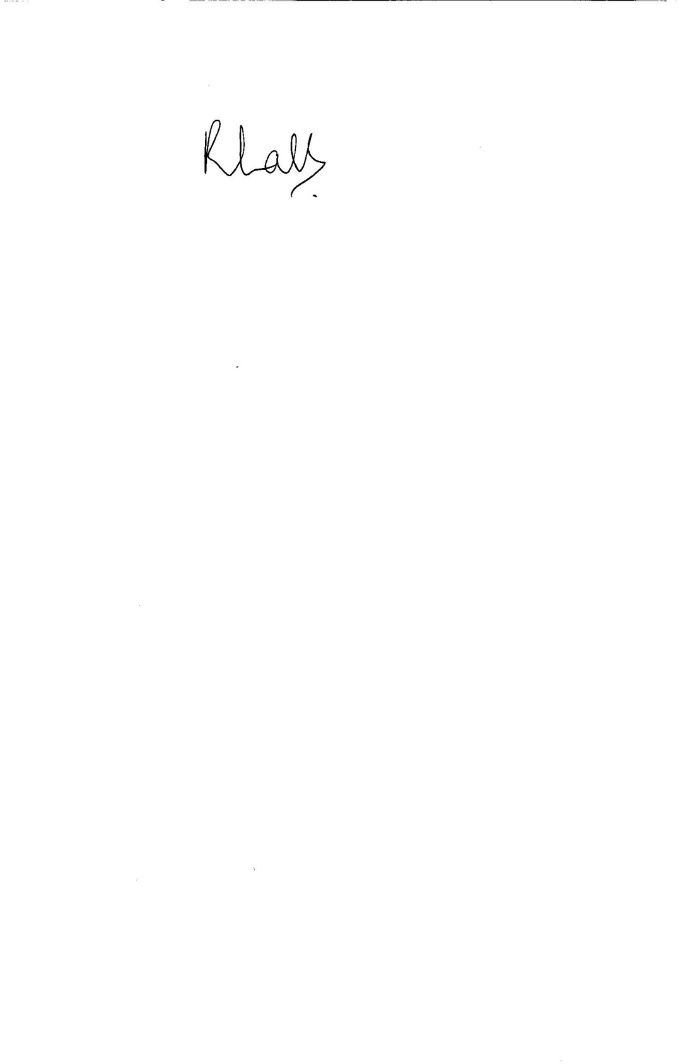 | Date:4.12.2023 |
| Chief Investigator | Name: Dr Gordon McGregor, UHCW | |
|  | 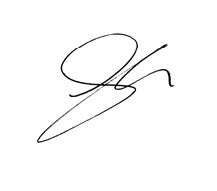Signature: | Date: 4.12.2023 |
| Senior Statistician Data Monitoring Committee | Name: Professor Stephen Walters, University of Sheffield | |
|  | Signature:  | Date: 09/11/2023 |
| Senior Statistician Trial Steering Committee | Name: Dr Ivonne Solis-Trapala, Keele Clinical Trials Unit | |
|  | Signature: 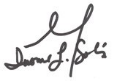 | Date:04/12/2023 |

#

Table of Contents

[Abbreviations 6](#_Toc171608478)

[List of authors and reviewers 7](#_Toc171608479)

[Roles and responsibilities 8](#_Toc171608480)

[Trial Statisticians 8](#_Toc171608481)

[Health Economists 8](#_Toc171608482)

[Data Monitoring Committee (DMC) 8](#_Toc171608483)

[SECTION 1: Summary of SPHERe 8](#_Toc171608484)

[1.1 Introduction 8](#_Toc171608485)

[1.2 Background and rationale 9](#_Toc171608486)

[1.3 Trial design 9](#_Toc171608487)

[1.4 Objectives of the trial 10](#_Toc171608488)

[1.5 Target population 10](#_Toc171608489)

[1.6 Outcomes of the trial 11](#_Toc171608490)

[SECTION 2: MONITORING OF THE TRIAL 12](#_Toc171608491)

[2.1 Operational (logistical) and trial management monitoring of patients 12](#_Toc171608492)

[2.1.1 Screening data 12](#_Toc171608493)

[2.1.2 Recruitment 12](#_Toc171608494)

[2.1.3 Withdrawal/death 12](#_Toc171608495)

[2.1.4 Protocol violations and deviations 13](#_Toc171608496)

[2.1.5 Safety data 13](#_Toc171608497)

[2.2 Statistical monitoring 13](#_Toc171608498)

[2.2.1 Randomisation 13](#_Toc171608499)

[2.2.2 Adherence to the intervention 13](#_Toc171608500)

[2.2.3 Blinding 13](#_Toc171608501)

[2.2.4 Sample size 14](#_Toc171608502)

[2.2.5 Hypothesis framework 15](#_Toc171608503)

[SECTION 3: CLINICAL OUTCOMES AND ANALYSIS DATASETS 15](#_Toc171608504)

[3.1 Outcome variables (primary and secondary) 15](#_Toc171608505)

[3.2 Type of populations 17](#_Toc171608506)

[3.3 Statistical interim analyses and stopping guidance. 17](#_Toc171608507)

[SECTION 4: MAIN STATISTICAL ANALYSIS AND THE ESTIMAND FRAMEWORK 17](#_Toc171608508)

[4.1 General considerations 17](#_Toc171608509)

[4.2 Confidence intervals and p values 18](#_Toc171608510)

[4.3 Primary outcome and the Estimand framework 18](#_Toc171608511)

[4.4 Analysis of the primary outcome 19](#_Toc171608512)

[4.5 Sensitivity analyses for the primary outcome 19](#_Toc171608513)

[4.6 Additional analyses 20](#_Toc171608514)

[4.6.1 Imputation analysis 20](#_Toc171608515)

[4.6.2 Bayesian analysis 20](#_Toc171608516)

[SECTION 5: Other data and analyses 20](#_Toc171608517)

[5.1 Baseline Data 20](#_Toc171608518)

[5.2 Secondary outcome data 21](#_Toc171608519)

[SECTION 6: Other analyses 21](#_Toc171608520)

[6.1 pre-specified subgroup analyses 21](#_Toc171608521)

[6.2 Harms 22](#_Toc171608522)

[6.3 Statistical software 22](#_Toc171608523)

[SECTION 7: Health Economics Analysis Plan (HEAP) 22](#_Toc171608524)

[Purpose of health economics analysis plan 22](#_Toc171608525)

[7.1 General principles for the primary health economic analysis 22](#_Toc171608526)

[7.1.1 Type of economic evaluation 22](#_Toc171608527)

[7.1.2 Perspective 22](#_Toc171608528)

[7.1.3 Time Horizon 23](#_Toc171608529)

[7.1.4 Discounting 23](#_Toc171608530)

[7.1.5 Clustered data structure 23](#_Toc171608531)

[7.1.6 Intention to treat 23](#_Toc171608532)

[7.2 Missing data 23](#_Toc171608533)

[7.2.1 Multiple Imputation 23](#_Toc171608534)

[7.3 Outcomes 24](#_Toc171608535)

[7.3.1 Primary health economic outcome 24](#_Toc171608536)

[7.4 Resource use and costing 24](#_Toc171608537)

[7.4.1 Direct intervention resource use and costs 25](#_Toc171608538)

[7.4.2 Healthcare and social care resource use 26](#_Toc171608539)

[7.4.3 Training costs 27](#_Toc171608540)

[7.4.4 Wider costs 28](#_Toc171608541)

[7.5 Data integrity 28](#_Toc171608542)

[7.6 Statistical analysis 28](#_Toc171608543)

[7.6.1 Descriptive analysis 28](#_Toc171608544)

[7.6.2 Addressing missing data with multiple imputation 28](#_Toc171608545)

[7.6.3 Cost-effectiveness analysis 29](#_Toc171608546)

[7.6.4 Value of information analysis 30](#_Toc171608547)

[7.6.5 Decision modelling 30](#_Toc171608548)

[SECTION 8: Reference (Statistical Analysis Plan) 31](#_Toc171608549)

[SECTION 9: Reference (Health Economics Analysis Plan) 32](#_Toc171608550)

[SECTION 10: Template Tables (Statistical Analysis Plan) 34](#_Toc171608551)

[SECTION 11: Template Tables (Health Economics Analysis Plan) 56](#_Toc171608552)

[Table 1: Completeness of data by follow-up visit 56](#_Toc171608553)

[Table 2: Health Status, resource use and cost (complete cases) 56](#_Toc171608554)

[Table 3: Cost-effectiveness results 58](#_Toc171608555)

# Abbreviations

| **Abbreviation** | **Explanation** |
| --- | --- |
| AE | Adverse events |
| ATS | American Thoracic Society |
| CACE | Compliers average causal effect |
| CAMPHOR | Cambridge Pulmonary Hypertension Outcome Review |
| CI | Confidence interval |
| CONSORT | Consolidated *S*tandards *o*f *R*eporting *T*rials |
| CTEPH | Chronic thromboembolic pulmonary hypertension |
| DMC | Data Monitoring Committee |
| ERS | European Respiratory Society |
| FSS | Fatigue Severity Scale |
| HADS | Hospital Anxiety and Depression Scale |
| ICC | Intra cluster coefficient |
| IQR | Interquartile range |
| ISRCTN | International Standard Randomised Controlled Trial Number |
| ISWT | Incremental shuttle walk test |
| ITT | Intention to treat |
| MI | Multiple Imputation |
| NIHR | National institute for health and Care research |
| PIC | Participants Identification Centres |
| PIL | Patient Information Leaflets |
| QoL | Quality of life |
| RCT | Randomised controlled trial |
| PAH | Pulmonary arterial hypertension |
| PH | Pulmonary hypertension |
| SPHERe | Supervised Pulmonary Hypertension Exercise Rehabilitation |
| SAP | Statistical analysis plan |
| SAE | Serious adverse event |
| SD | Standard deviation |
| TSC | Trial Steering Committee |
| UHCW | University Hospitals Coventry & Warwickshire |
| WCTU | Warwick Clinical Trials Unit |
| WHO | World Health Organisation |
| 6MWT | Six-minute walk test |

# List of authors and reviewers

*Authors for statistical analysis plan*

Mariam Ratna, Trial Statistician, Warwick Clinical Trials Unit (WCTU), University of Warwick

Katie Booth, Trial Statistician, Warwick Clinical Trials Unit (WCTU), University of Warwick

Chen Ji, Trial Statistician, Warwick Clinical Trials Unit (WCTU), University of Warwick

*Authors for health economics analysis plan*

James Mason, Health Economists, Warwick Clinical Trials Unit (WCTU), University of Warwick

Rebecca Kandiyali, Health Economists, Warwick Clinical Trials Unit (WCTU), University of Warwick

*Reviewers*

Professor Ranjit Lall, Lead Trial Statistician, Warwick Clinical Trials Unit (WCTU), University of Warwick

Dr Gordon McGregor, Chief Investigator (CI), Department of Cardio-pulmonary rehabilitation, University Hospitals Coventry & Warwickshire

Professor Stephen Walters, DMC Chair and Statistician, University of Sheffield

Dr Ivonne Solis-Trapala, TSC Chair and Statistician, Keele Clinical Trials Unit

Members of TMG

# Roles and responsibilities

## Trial Statisticians

Professor Ranjit Lall, Dr Chen Ji, Katie Booth, Dr Mariam Ratna

Role: To develop the statistical analysis plan and conduct the final analysis.

## Health Economists

Professor James Mason, Dr Rebecca Kandiyali

Role: To develop the health economics analysis plan and conduct the final analysis.

## Data Monitoring Committee (DMC)

Professor Stephen Walters (Chair), University of Sheffield

Dr Joanna Pepke-Zaba, Royal Papworth Hospital NHS Foundation Trust

Professor Garry Tew, York St John University

Role: To review and approve the statistical analysis plan ahead of final analysis.

# SECTION 1: Summary of SPHERe

## 1.1 Introduction

This document details the proposed presentation and analysis for the main paper(s) reporting results from the multi-centre randomised controlled trial SPHERe (ISRCTN 10608766) to investigate a programme of supervised exercise rehabilitation, with psychosocial and motivation support, on walking distance, disease-specific and health-related quality of life (HRQoL), in people with Pulmonary Hypertension (PH).

The results reported in the funder report and main paper(s) will follow the strategy set out here. Any subsequent analysis of a more exploratory nature will not be bound by this strategy and will be detailed in a separate statistical analysis plan (SAP). Suggestions for subsequent analyses by oversight committees, journal editors or referees, will be considered carefully in line with the principles of this SAP.

Any deviations from the final, approved SAP will be described and justified in the final report to the funder. The statistical analysis will be carried out by appropriately qualified and experienced medical statisticians, who will ensure the integrity of the data during processing.

## 1.2 Background and rationale

Pulmonary hypertension (PH) is a debilitating long-term condition characterised by severe exercise intolerance [1]. Five distinct sub-groups were identified by the guidance from the World Symposium on PH [2]:

1. Group 1 - Pulmonary arterial hypertension (PAH)
2. Group 2 - PH due to left heart disease
3. Group 3 - PH due to lung diseases or hypoxia, or both
4. Group 4 - Chronic thromboembolic pulmonary hypertension (CTEPH)
5. Group 5 - PH with unclear multifactorial mechanisms.

Drug treatment and pulmonary endarterectomy may help people with PAH [3] and CTEPH [4], respectively, but benefit is often limited. For people with PH secondary to cardiac or pulmonary disease (groups 2 & 3), there are no specific treatments of proven benefit [5, 6]. In-patient exercise rehabilitation may have a short-term benefit on exercise capacity in selected people with PAH or CTEPH. However, it is not known if these benefits extend to PH groups 2, 3, & 5, or if exercise rehabilitation delivered in an NHS out-patient/home-based setting is effective or cost effective, or if there are any long-term health benefits or harms. Current exercise rehabilitation interventions for PH do not explicitly target modifiable psychosocial factors such as depression, anxiety and/or fear of exercise.

To address these evidence gaps, a remotely supervised, home based exercise rehabilitation, with psychosocial and motivational support intervention will be tested.

Please refer to latest version of the protocol: presently version 8.0, 01 November 2022.

## 1.3 Trial design

SPHERe is a multi-centre, randomised controlled trial (RCT) testing the clinical and cost-effectiveness of the SPHERe intervention vs best-practice usual care in people with pulmonary hypertension from up to 20 NHS exercise centres (principally in the East and West Midlands). The trial design includes:

1. An intervention development phase, to refine recruitment process, confirm feasibility and safety of intervention delivery, manualise practitioner training, and prepare study set-up.
2. An internal pilot phase at multiple sites (5 centres), to test trial recruitment and study procedures.
3. A multicentre RCT with an embedded process evaluation at up to 20 NHS rehabilitation centres in England, Scotland, and Wales.

## 1.4 Objectives of the trial

#### 1.4.1 Primary objective

To determine whether the remotely supervised, home-based pulmonary hypertension exercise (SPHERe) rehabilitation intervention can improve exercise capacity, measured using the incremental shuttle walk test (ISWT), at **four months** compared to best-practice usual care for people with pulmonary hypertension.

#### 1.4.2 Secondary objectives

To determine if the SPHERe intervention compared to best-practice usual care in people with pulmonary hypertension impacts on the following outcomes over 12 months:

1. Incremental shuttle walk test (ISWT).
2. Disease-specific HR-QoL: Cambridge Pulmonary Hypertension Outcome Review (CAMPHOR)
3. Health utility: EQ-5D-5L
4. Emotional well-being: Hospital anxiety and Depression Scale (HADS)
5. Self-efficacy: Generalised Self-efficacy Scale (GSES)
6. Fatigue: Fatigue Severity Scale (FSS)
7. WHO Functional Class
8. Medication Use
9. Time to Clinical Worsening
10. Health and Social Care Resource Use
11. Hospital Admissions
12. Adverse Events
13. All-cause Mortality

## 1.5 Target population

People that meet the following criteria are the desired population for the trial.

**Inclusion criteria**

1. Aged ≥18 years with confirmed PH (groups 1 to 5)
2. Clinically stable: Groups 1, 4, & 5 - stable on optimal PH specific drug therapy (for those in whom it is appropriate) for at least 1 month, or evidence that these drugs cannot be tolerated. Groups 2 & 3 - stable on drug therapy for underlying cardiac or pulmonary disease for at least one month. Clinical stability will be confirmed by the lead practitioner at each site, in consultation with the responsible clinician, and determined as: presenting with, reproducible, manageable symptoms, not requiring any treatment other than routine follow-up care, and no PH related hospital admission in the last four weeks.
3. WHO functional class II, III or IV
4. Fluent in spoken English to allow engagement with intervention and physical outcome measures
5. Live within reasonable travelling distance (as defined by the participant) of a SPHERe exercise rehabilitation centre (for outcome assessments only)
6. Able to make suitable travel arrangements to attend clinic (for outcome assessments only)
7. Access to appropriate IT infrastructure (computer, laptop, tablet, smart phone, email and internet connection)
8. Ability to provide informed consent.

**Exclusion criteria**

1. Absolute contra-indications to exercise as per international clinical guidelines
2. PH related complications, or comorbidities severe enough to prevent attendance at a SPHERe centre, or participation in exercise rehabilitation
3. Any mental health issue that will prevent engagement with study procedures
4. Previous randomisation in the present trial
5. Pregnant at time of recruitment.

## 1.6 Outcomes of the trial

**Primary outcome**

Exercise capacity as determined by distance walked in the Incremental shuttle walk test (ISWT) at four months post-randomisation [7]

**Secondary outcomes (4 and 12 months)**

Disease specific health-related quality of life (HR-QoL): Cambridge Pulmonary Hypertension Outcome Review (CAMPHOR) [8]

Emotional well-being: Hospital Anxiety and Depression Scale (HADS) [9]

Self-efficacy: Generalised self-efficacy scale (GSES) [10]

Fatigue: Fatigue Severity Scale (FSS) [11, 12]

World Health Organisation (WHO) functional class [13]

Medication use

Time to clinical worsening [14]

All-cause mortality

Safety Outcomes

All cause hospital admissions from GP records

**Health Economics Outcomes:**

Health utility: EQ-5D-5L [15]

Health and social care resource use

# SECTION 2: MONITORING OF THE TRIAL

Monitoring of the trial is a continuous process, from the start to the end of the study. At the end of the trial two aspects related to monitoring will be examined:

1. Operational (logistical) and Process Management monitoring;

2. Statistical monitoring (assessment of bias – as stated in the protocol).

## 2.1 Operational (logistical) and trial management monitoring of patients

### 2.1.1 Screening data

Participants are identified and screened by the clinical care team via multiple, co-ordinated screening strategies. PIC (Participants Identification Centres) sites are used to identify potential research participants. The CONSORT (Consolidated Standards of Reporting Trials) flowchart will illustrate people who were eligible for the trial and were subsequently randomised as well as those who were not eligible and did not participate (FIGURE 1 on Section 10).

### 2.1.2 Recruitment

The CONSORT diagram (FIGURE 1 on Section 10) will illustrate participant flow throughout the trial and will describe the following: number of participants randomised, allocated to each intervention, delivered and not delivered intervention, lost to follow-up, and included in ITT analysis population at different time points.

### 2.1.3 Withdrawal/death

The number and percentage of participants withdrawn from intervention and/or from follow-up before 12 months post-randomisation will be reported for each randomised group (see Table 6, Table 9, Table 10 on Section 10). Level and reasons of withdrawal or loss to follow-up will be reported (see Table 7 and Table 8 on Section 10). Any follow-up that is after 4-months up to 8-months will still be considered as 4-months. Therefore, if follow-up occurs after 4-months (from 4- to 8-months) this will still be taking as a valid assessment for the 4-month ISWT score. And if the ISWT score obtained after 8 months then it can be used in multiple imputation for the primary outcome (12 months). Duration of time in follow-up to the ISWT will also be adjusted for in the statistical analysis.

### 2.1.4 Protocol violations and deviations

Protocol violations will be tabulated as in Table 11 on Section 10.

### 2.1.5 Safety data

The AEs and SAEs will be reported by treatment arm in Table 20 and Table 21 on Section 10. The list of all TNOs (Trials Numbers) for AEs and SAEs will also be reported by treatment arm in Table 22 and 23 on Section 10.

## 2.2 Statistical monitoring

### 2.2.1 Randomisation

The participants will be randomised to the SPHERe intervention or best practice usual care using a computer-generated randomisation sequence, performed by minimisation, based on centre, PH group, and World Health Organisation (WHO) functional class.

Table 2 on Section 10 illustrates the randomisation balance for the strata (WHO Class, PH Group and Centre/site) and by treatment.

### 2.2.2 Adherence to the intervention

For the intervention group, partial adherence will be defined as completion of the initial assessment/familiarisation and at least half of the prescribed group online home exercise sessions, guided home exercise, and psychosocial/motivational sessions. Full adherence will be considered as attending at least 6 out of 8 (75%) exercise sessions and at least 4 out of 6 (67%) psychosocial/motivational support sessions. The psychosocial/motivational sessions, in themselves, are designed not only to help support daily activities, but equally to encourage adherence.

### 2.2.3 Blinding

To maintain blinding, all follow-up data will be collected by staff not directly involved in intervention delivery who are blind to treatment allocation. Participants will be asked not to tell the assessing practitioner their group allocation. The quality of blinding will be assessed by asking the assessor which treatment they thought each participant had received (see Table 24 on Section 10).

### 2.2.4 Sample size

The primary outcome is **distance walked measured using the ISWT at four months post-randomisation.** As there are no directly applicable ISWT data with which to calculate a sample size, or previously defined worthwhile effect sizes on ISWT for people with PH, to inform a sample size calculation, 6MWT data have been used to inform the sample size estimation. The 6MWT distance, unlike the alternative approach of using a standardised mean difference, has the advantage that it is meaningful to our participants and grounded in clinical reality.

Our original sample of size 246 participants for PH groups 2 and 3 (352 for all PH groups) is required to show a difference in 6MWT distance of 45m, with a standard deviation of 90 between the treatment arms at 5% level of significance and 90% power. Allowing for a clustering effect in the intervention arm, we assume that an average group size will consist of twelve participants and an intra cluster coefficient (ICC) of 0.03. This equates to 114 participants in the control arm and 132 participants in the intervention arm (control:intervention = 1:1.15), using computations recommended by Moerbeek [16]

#### 2.2.4.1 Revised Sample size

During the lifetime of the trial, we revised our sample size using observed parameters from 79 randomisations and 43 participants with complete primary outcome data. We additionally included the observed correlation coefficient between baseline and follow up ISWT:

1. Number of patients in PH groups 2/3 = 35/79 (44%)
2. Intervention group size between 5 and 10
3. Observed ICC = 0.03
4. Allocation ratio = 1.04:1 (cluster size =5) and 1.10:1 (cluster size =10)
5. Effect size = 0.5
6. Lost to follow-up =24%
7. Correlation coefficient = 0.8.

To show our target difference, with this level of correlation, we need to recruit **85-90 participants with type 2/3 PH** (depending on the cluster size). This is the number of participants we would need to show a benefit in the key group of people with type 2/3 PH (specified in the original NIHR HTA brief). However, the proportion of people with type 2/3 PH is smaller than anticipated at the time of study design. This may reflect the move to online intervention delivery during the COVID-19 pandemic, meaning fewer older people with type 2/3 PH are able/willing to access the intervention (issues with access/competence in use of technology). Around 44% of our recruited participants have type 2/3 PH compared to our pre-study estimate of 70%. Thus, we are aiming for an overall target of **around 200 participants** but with an intention to stop recruitment when we have 85-90 participants with type 2/3 PH.  The final overall recruitment may be substantially less than 200.

This change in sample size was fully reviewed and approved by the TSC, DMC, Sponsor and Funder (NIHR HTA) in November 2022.

### 2.2.5 Hypothesis framework

We hypothesise that the SPHERe intervention will improve clinical, and patient reported, outcomes when compared to best practice usual care. For each of the primary and secondary outcomes, the null hypothesis will be that there is no true difference in treatment effect between the intervention arms.

# SECTION 3: CLINICAL OUTCOMES AND ANALYSIS DATASETS

## 3.1 Outcome variables (primary and secondary)

| **Outcomes** | **Time point** | **Scoring** |
| --- | --- | --- |
| **Primary outcome** | | |
| Exercise capacity as determined by distance walked in the Incremental shuttle walk test (ISWT) | 2 | Exercise capacity as determined by distance walked measured in meters using the Incremental Shuttle walk test (ISWT) at four months post-randomisation. ISWT will be performed as per European Respiratory Society (ERS)/American Thoracic Society (ATS) guidelines [12]. The externally paced ISWT is a simple assessment of maximal exercise capacity and, in PH, is sensitive to treatment effect, predicts mortality, and has no ceiling effect [7]. |
| **Secondary outcomes** | | |
| Incremental shuttle walk test (ISWT) | 1, 3 | See above |
| Disease specific health-related quality of life (HR-QoL) | 1,2,3 | Cambridge Pulmonary Hypertension Outcome Review (CAMPHOR) [13]. This is widely used as a clinical and research tool in PH, displaying good construct validity and reproducibility. It has three sub-scores:  Symptoms score: It consists of a 25-item symptoms scale (scored 0–25; “yes/true” scores 1 and “no/not true” scores 0), Activity score: a 15-item functioning scale (scored 0–30; all items have three possible responses which score 0 to 2) and  Quality of life score: a 25-item QoL scale (scored 0–25; “yes/true” scores 1 and “no/not true” scores 0). For all scales, a low score indicates a better status [8]. |
| Health utility | 1,2,3 | EQ-5D-5L [15]. A validated, generic HR-QoL measure consisting of five dimensions, each with five levels of response. Each combination of answers can be converted into a health utility score. It has good test-retest reliability, is simple to use, and gives a single preference-based index value for health status that can be used for cost-effectiveness analysis. The EQ-5D score ranges from <0-1 where a higher score reflects better quality of life.  This SAP will only include the health utility data of survivor participants, but the enclosed HEAP will incorporate all data in the analysis, including deceased participants (utility score = 0). |
| Emotional well-being | 1,2,3 | Hospital Anxiety and Depression Scale (HADS) [9]. A 14-item screening questionnaire from which an anxiety and depression subscale can be derived. Sub-score values of 8 and above identify increased symptoms of anxiety and/or depression. Not used extensively in PH but included as a well validated measure in clinical populations. The scores are simply summated to give an anxiety and depression score both ranging from 0-21 where a higher score reflects more severe symptoms of anxiety and depression. |
| Self-efficacy | 1,2,3 | The Generalised self-efficacy scale (GSES) is 10-item psychometric scale designed to assess optimistic self-beliefs to cope with a variety of difficult demands in life that are key targets of the behavioural component of the SPHERe intervention. The total score is calculated by finding the sum of all items. The total score ranges between 10 and 40, with a higher score indicating more self-efficacy [10]. |
| Fatigue | 1,2,3 | Fatigue Severity Scale (FSS) [11]. A nine-item questionnaire validated for evaluating disabling fatigue and previously used in PH [12]. Each item is rated on a seven-point scale, from strongly disagree to strongly agree. A total score is derived from all nine questions; a higher score indicates a greater impact of fatigue on everyday activities. |
| World Health Organisation (WHO) functional class | 1,2,3 | A modified New York Heart Association functional classification system adopted by WHO and used ubiquitously in PH. Participants are graded on their ability to perform physical tasks, and classified as (I) no limitation, (II) mild limitation, (III) marked limitation, (IV) unable to perform any activity [13]. This will be assessed by a research practitioner at each trial assessment. |
| Medication use | 1,2,3 | Class, drug, dose and frequency of all regular medication will be recorded. Participants will be asked to bring their repeat prescription to outcome assessment appointments. |
| Time to clinical worsening | 2,3 | Defined as one of; PH related death; listing for/completed lung transplant; hospitalisation for PH; clinical worsening leading to initiation of new PH treatment; decreased WHO functional class and ≥15% decrease in ISWT distance [14] |
| Health and social care resource use | 2,3 | Participant self-report and NHS records. The primary health-economic analysis will concentrate on direct intervention and healthcare/personal social services costs, while wider impact (societal) costs will be included within the sensitivity analyses. Participants will complete resource use questionnaires at four- and 12-month follow-up points, to collect resource use data associated with the interventions under examination. Participants may use a resource use diary as an aide memoire to help record their resource use between baseline and follow-up. We are additionally resourced to collect data from GP practices directly. This will involve members of the trial team going into individual practices and completing a duplicate CRF (relevant fields only) for the patient. The duplicate CRF would essentially be a copy of section 9 questions 1-5; these fields cover: inpatient care, outpatient care, A&E, other visits and admissions and community care. |
| All cause hospital admissions from GP records | 2,3 | See above. |
| All-cause mortality | 2,3 | Participants will be flagged with NHS digital to ensure notification of any deaths and cause of death both during the current trial and for longer term follow-up. |
| **Safety Reporting** |  |  |
| Adverse Events and Serious Adverse Events | Throughout the trial |  |

1 = Baseline; 2 = 4 month after randomisation; 3 = 12 month after randomisation

## 3.2 Type of populations

The primary analysis and any secondary analyses will be applied to an all-randomised population on an ITT basis. That is, any patient randomised into the trial, regardless of whether they received trial intervention and regardless of protocol deviations, unless specified above.

**Observed dataset**

This will comprise of all the data observed with missing values.

**Imputed dataset**

The missing data information will be indicated by footnotes for table and figures. If the amount of missing data differs between the treatment arms, potential reasons will be explored. See section 4.6.1.

## 3.3 Statistical interim analyses and stopping guidance.

There are no formal interim analyses for this study.

# SECTION 4: MAIN STATISTICAL ANALYSIS AND THE ESTIMAND FRAMEWORK

## 4.1 General considerations

In general, continuous baseline and outcome data will be summarised with descriptive statistics, including n, mean, standard deviation, median, interquartile range and n of missing data. Categorical baseline and outcome data will be summarised with frequency counts and percentages. In addition, graphical presentation will be made for several variables that are specified individually. In addition, outcome data will be compared by treatment arms. Odds ratio (OR) with 95% confidence interval (CI) will be reported for categorical outcomes and mean difference with 95% CI will be reported for continuous outcomes, unless stated otherwise.

## 4.2 Confidence intervals and p values

Treatment effects will be presented, with appropriate 95% confidence intervals, for both the unadjusted and adjusted analyses. Tests will be two-sided and considered to provide evidence for a significant difference if p-values are less than 0.05 (5% significance level). All analyses will be conducted as intention to treat unless otherwise specified.

## 4.3 Primary outcome and the Estimand framework

In line with the ICH E9 (R1) addendum on Estimand and sensitivity analyses in clinical trials, Table 1 presents the Estimand framework in relation to the primary outcome of the trial.

| Table 1: Estimand framework for the primary outcome of SPHERe | |
| --- | --- |
| **Estimand attribute** | **Description** |
| *Objective* | To determine whether the remotely supervised, home-based pulmonary hypertension exercise (SPHERe) rehabilitation intervention can improve walking distance and disease-specific health-related QoL at four months post-randomisation compared to best-practice usual care for people with pulmonary hypertension. |
| *Treatment conditions* | The improvement in walking distance between those randomised to the SPHERe intervention compared to usual care for people with pulmonary hypertension. |
| *Population* | Adults (aged ≥18 years) participants with confirmed pulmonary hypertension (groups 1 to 5). |
| *Variable (primary outcome)* | Exercise capacity as determined by distance walked measured using the Incremental Shuttle walk test (ISWT) at four months post-randomisation. |
| *Summary measure* | Difference in average distance walked measured using the Incremental Shuttle walk test (ISWT) at four months post-randomisation between the treatment groups. |
| *Handling Intercurrent events* | Post-randomisation events which may affect the interpretation or occurrence of the primary outcome include:  ICE 1: Discontinuation of the allocated treatment (i.e., withdrawal from treatment).  ICE 2: Non- adherence to allocated treatment (as defined in section 2.2.2). |
| *Strategies for handling intercurrent events* | ICE 1:  *Treatment policy-* analysis as observed.  ICE 2:  *Principal stratum strategy*- assessing the effect of the intervention, having adjusted for the non- adherence (using CACE analysis). |

## 4.4 Analysis of the primary outcome

Primary outcome (ISWT) data will be summarised with descriptive statistics (n, mean, standard deviation, median, interquartile range, and n of missing data).

The primary analyses will use a multilevel mixed effects linear regression (heteroscedastic) model to estimate the treatment effects (95% confidence intervals (CI)), adjusted for stratification variables, important patient-level covariates and practitioner effect. The reason for using heteroscedastic model is the variance is different between the arms as the control arm is non-clustered and the intervention arm is clustered [17]. In the trial, the control arm receives individual therapy whereas the intervention arm receives group therapies. The groups will be treated here as random effects. If there are negligible random effects, then a standard linear regression model will be used for the analysis. The covariates include:

- WHO functional class (stratification variable)
- PH group (stratification variable)
- Age
- Gender
- Centre (stratification variable)
- Duration of time in follow-up to the ISWT

Both adjusted and unadjusted estimates will be presented.

## 4.5 Sensitivity analyses for the primary outcome

4.5.1 A sensitivity analysis for the primary outcome will be conducted for the ICE2 (non- adherence), stated in the estimand framework (Table 1).

**Sensitivity analysis for ICE2 (non- adherence):**

The effect of non-adherence will be assessed using a complier average causal effect (CACE) analysis approach for the primary outcome. Under some assumptions, a structural mean model with the inclusion of an instrumental variable was fitted to estimate the treatment effect among those who complied the study [18]. Analysis will be adjusted for the covariates specified in the primary analysis. The CACE analysis will also be replicated using different definitions of adherence (as stated in section 2.2.2).

## 4.6 Additional analyses

### 4.6.1 Imputation analysis

The primary analysis will be replicated to analyse the imputed datasets if imputation is deemed appropriate. The imputation analysis will be carried out using multiple imputation and the MICE (multiple imputation by chained equations) procedure in STATA. MICE operate under the assumption that given the variables used in the imputation procedure, the missing data are Missing at Random (MAR), which means that the probability that a value is missing depends only on observed values and not on unobserved values. The list of variables included in the adjusted model mentioned earlier will be included in the imputation model.

The imputation analyses for the primary outcome will be conducted for dealing with those missing values such as:

1) Predicting the missing primary outcome (ISWT) data at 4-month FU using the strong predictors (secondary outcomes at 4-month FU). Strong predictors will be identified by developing a prediction model using the available primary outcome data as the dependent variable. Then the multiple imputation will be carried out for imputing the further missing primary outcome data that cannot be predicted by the prediction model (i.e. missing data in both primary outcome and any strong predictors).

2) Imputing all missing ISWT data at 4-month FU using the baseline ISWT data and other baseline demographics and secondary outcomes. The imputation analysis will be carried out using multiple imputation and the MICE (multiple imputation by chained equations) procedure in STATA.

### 4.6.2 Bayesian analysis

We will present probabilities (non-informative prior and informative priors) for achieving the desired effect size in each of the groups using the magnitude-based inference approach [19]. The determination of the priors is under further discussion with our clinical colleagues and review of the literature.

# SECTION 5: Other data and analyses

## 5.1 Baseline Data

Baseline data will be summarised to check comparability between treatment arms, and screening data will be checked to highlight any characteristic differences between those individuals in the trial, those ineligible, and those eligible but withholding consent. The number and percentage will be presented for categorical variables. The mean and standard deviation or the median and the interquartile range (IQR) will be presented for continuous variables, or the range if appropriate. There will be no tests of statistical significance performed nor confidence intervals calculated for differences between randomised groups on any baseline variable.

## 5.2 Secondary outcome data

The secondary outcomes will be assessed using ITT approach. First, the secondary outcomes will be summarised with descriptive statistics (count, mean, standard deviation, median, and interquartile range for numerical variables, and frequency and percentage for categorical variable). For continuous outcomes, a linear regression (heteroscedastic) model will be used to estimate the treatment effects (95% confidence intervals (CI)), adjusted for stratification variables, important patient-level covariates and practitioner effect. The normality assumptions of the variables will be checked. The groups will be treated here as random/practitioner effects. If there are negligible group and centre effect, then standard linear regression will be used for the analysis. Categorical data will be assessed in a similar way, using logistic regression models and odds ratio with 95% CI will be reported. For the time to clinical worsening event, Kaplan-Meier curve will be used, and the hazard ratio will be estimated with 95% CI for each treatment. The proportional hazards assumption will also be tested before applying Kaplan-Meier.

# SECTION 6: Other analyses

## 6.1 pre-specified subgroup analyses

This trial is not powered to identify interactions. Exploratory sub-group analyses will examine the effects for pre-specified sub-groups:

1. PH group (2 & 3 vs 1, 4, &5). Analysis will be conducted using formal tests of interaction. We will, however, present the effect size for pooled groups 2 & 3 as a separate analysis. The effect size and 95% CI for each five PH groups will also be presented separately, without drawing any inference, for the benefit of future systematic reviewers. Main outcomes will be presented by treatment arm to inform decision makers and guide developers interested in specific groups.
2. WHO functional class (Class II vs Class III&IV). We will do a formal test for the interaction between treatment assignment and sub-group and present the results by sub-group for the benefit of future systematic reviewers.

The subgroup analysis will be conducted using ITT approach. It will involve modelling the primary outcome as explained in Section 4.2 adding an interaction term into the model for treatment and subgroup variable. The 95% confidence intervals for the interaction terms will be reported.

## 6.2 Harms

The frequency and percentage (%) of serious adverse events (SAE) and adverse events (AE) in the trial will be reported by treatment arm. The event type, severity assessment, expectedness and relatedness to intervention will also be summarised by treatment arm.

## 6.3 Statistical software

All analyses will be conducted using Stata/SE version 18 (or later), SAS version 9 (or later), or R version 4 (or later).

# SECTION 7: Health Economics Analysis Plan (HEAP)

# Purpose of health economics analysis plan

The objective of the health economics analysis is to inform decision makers regarding the cost-effectiveness of the ‘Supervised Pulmonary Hypertension Exercise Rehabilitation’ (SPHERe) intervention compared to usual care. This entails a systematic analysis of both the costs and consequences of both treatment options. The purpose of the health economics analysis plan (HEAP) is to outline a framework of methods that will be used to analyse the health economic components of the trial ensuring the integrity of the cost-effectiveness analysis.

## General principles for the primary health economic analysis

Given the UK focus of the SPHERe trial, we will adopt principles that best meet the requirements of UK decision makers. The methods of economic evaluation will therefore be informed by the National Institute for Health and Care Excellence (NICE) guide to the methods of technology appraisal [9].

### Type of economic evaluation

As recommended, the primary health economic analysis will be a cost-utility analysis with incremental quality adjusted life years (QALYs) as the health economic outcome [9]. Following NICE guidance, the EQ-5D-5L will be used for the construction of QALYs (see section 2.1.1) [9].

### Perspective

A healthcare and personal social services (PSS) will be adopted as recommended by NICE [9]. We will however consider wider societal costs within a sensitivity analysis.

### Time Horizon

The primary health economic analysis will run concurrently the effectiveness analysis. Outcomes will be collected at baseline, four months post-randomisation, and 12 months post-randomisation. The time horizon will therefore be the 12-month period post-randomisation. Should outcomes not have converged after 12 months, we will consider the development of a decision analytic model to extrapolate the cost-effectiveness results over a lifetime horizon (see section 5.5).

### Discounting

Given the trial-based analysis has a time-horizon of 12 months, costs and QALYs will not be discounted. Should longer-term decision modelling be conducted, we will use the 3.5% annual discount rate as recommended by NICE to discount future costs and QALYs [9].

### Clustered data structure

Participants are being randomised at the individual level. However, there may be cluster level impacts relating to the centres that each patient is recruited from. We will therefore explore the degree to which clustering occurs within the data using the intra-cluster correlation coefficient (ICC) and then choose appropriate methods (e.g. multi-level modelling) accordingly.

### Intention to treat

The health economic analysis will adopt the principle of ‘intention to treat’ [10]. This means that the health economic analysis will analyse individuals according to the trial arms to which they were randomised.

## Missing data

Missing data is a common occurrence within randomised clinical trials and needs to be addressed within the health economic analysis [11]. Missing data will be explored, and if non-trivial (5% or more in either costs or QALYs) [12], the base case analysis will use multiple imputation [13] as the preferred method for estimating results in the presence of missing data.

### 7.2.1 Multiple Imputation

Simple methods to address missing data such as ‘mean imputation’ and ‘last observation carried forward’ have been criticised for inadequately characterising uncertainty and introducing bias [14]. Consequently, multiple imputation is the preferred method for imputing missing data. MI uses the observed data and samples from the predictive distribution to create multiple datasets [15]. Under the assumption of missing at random, this provides unbiased estimates; this allows uncertainty surrounding estimates to be maintained whilst allowing full use of the available data..

## 7.3 Outcomes

### 7.3.1 Primary health economic outcome

As recommended by NICE, incremental quality adjusted life years (QALYs) will be used as the primary outcome for the health economic analysis [9].

#### 7.3.1.1 Estimating QALYs

QALYs combine both mortality and morbidity into a single measure that can be compared across contexts within the healthcare service. To calculate QALYs it is necessary to combine a preference-based measure of quality of life with time. In this study, we are using the EQ-5D-5L [16] at three time points (baseline, 4 months, 12 months). The EQ-5D-5L is a preference-based measure of health-related quality of life and is recommended by NICE for use in economic evaluation [9]. The measure contains five dimensions of health, each containing five levels. There exist value-sets [17], [18] that allow the calculation of *utility* scores for any given set of responses to the EQ-5D-5L. A utility score is a score on an index scale of zero to one, where zero equals death, and one equals full health (negative (states worse than death) are possible). These utility values can be combined with time to derive QALYs. Although a UK specific EQ-5D-5L value set exists [17], it has been the subject of controversy [19]. Currently the most recent NICE manual [20] instead recommends the use of the Hernández Alava et al. et al [18] mapping function. This however may change in the interim period between the period of writing and the date of analysis. The choice of value set will therefore be made closer to the date of analysis and will be chosen in accordance with NICE guidelines at the point of analysis.

QALYs for each patient will be calculated by using the EQ-5D-5L utility values at baseline, 4 months and 12 months. QALYs will be calculated by linearly interpolating for the three time points and calculating the area under the curve using the trapezium rule [21]. QALYs will be calculated for each patient in the trial.

## 7.4 Resource use and costing

To calculate costs for use in cost-utility analysis it is necessary to capture information on resources used for both the control and intervention arm. Costs within this trial have the following components:

- Direct intervention costs (e.g. delivery of exercise programme)
- Direct healthcare and PSS costs (e.g. outpatient appointment)
- Training costs (e.g. clinician training)
- Other societal costs (e.g. absence from work)

NICE’s guide to methods of technology appraisal recommend costing from an NHS and personal social services (PSS) perspective [9]. The primary analysis will only consider the first three items; broader societal costs will be included within sensitivity analysis. To calculate costs, it is first necessary to capture resource use, and then apply unit costs. The price year for the analysis will be informed by the latest available base year for common costing resources at time of analysis.

### 7.4.1 Direct intervention resource use and costs

The SPHERe intervention can be split into four components: i) guided home exercise plan; ii) individual assessment and familiarisation; iii) supervised exercise programme; and iv) psychological coaching. The control arm however receives one 30-minute practitioner appointment in conjunction with a ‘British Lung Foundation’, ‘Keep Active’ booklet. The intervention components and associated resource use are summarised within Table 1 below. This table shows what the components are, how they will be collected and where unit cost sources may be sourced from.

Table 1: Direct intervention resource use and cost sources

| ***Intervention arm*** | | | |
| --- | --- | --- | --- |
| **Resource type** | **Resource use** | **How collected** | **Unit costs source** |
|  |  |  |  |
| Individual online assessment and familiarisation | 1 hour one to one appointment with practitioner. | Trial team | PSSRU unit costs |
| Supervised online group exercise session | One group exercise session per week | Trial team | PSSRU unit costs |
| Guided home exercise fitness programme | Manualised home exercise plan | Trial team | Invoice for exercise plan production |
| Group psychological and motivational support | Weekly group sessions | Trial Team | PSSRU unit costs |
| Coaching calls | Weekly calls between practitioner and patient | Trial team | PSSRU unit costs |
| Equipment: Bike rental, delivery, set-up and collection. | Time spent delivering and setting up exercise bikes, and collection. Bike rental costs. | Trial team | PSSRU unit costs, invoices for bike rental/delivery |
| Equipment: remote facilitation - Zoom/Beam and website hosting | Subscription and website hosting costs | Trial Team | Invoice for subscriptions and hosting |
| Equipment: pulse oximeter | Loan of pulse oximeter | Trial team | NHS supply chain |
| ***Control arm*** | | | |
| Online practitioner appointment | One appointment lasting 30 minutes | Treatment logs | PSSRU unit costs |
| ‘British Lung Foundation ‘Keep Active’ booklet | one ‘British Lung Foundation ‘Keep Active’ booklet | 1 per person | Printing costs |

### 7.4.2 Healthcare and social care resource use

In according with NICE guidance, we capture healthcare and PSS costs for both arms of the trial [9]. This will include, inpatient care, outpatient care, community care, accident and emergency admission, medication, and personal social services. The methods for capturing the resource use and the sources for unit costs are outlined in Table 2 below. Most of these resource use items will be captured with patient-completed case report forms (CRFs) at 4 and 12 months, whilst medication will be captured using a concurrent rolling medication log. We are additionally resourced to collect data from GP practices directly. This would involve members of the trial team going in to individual practices and completing a duplicate CRF (relevant fields only) for the patient. The duplicate CRF would essentially be a copy of section 9 questions 1-5; these fields cover: inpatient care, outpatient care, A&E, other visits and admissions and community care.

Table 2: Ongoing costs for both arms – health care costs and social costs

| **Resource type** | **Resource use** | **How collected** | **Unit cost sources** |
| --- | --- | --- | --- |
| Inpatient care | Specified within CRFs | CRFs at 4m and 12m with aide memoir triangulated with medical records. HRG4+ ‘Code to Group’ [22] used to allocate inpatient care to HRG groups for costing. | NHS Reference Costs and PSSRU |
| Outpatient care | Specified within CRFs | CRFs at 4m and 12m with aide memoir triangulated with medical records | NHS Reference Costs and PSSRU |
| Accident and emergency care | Specified within CRFs | CRFs at 4m and 12m with aide memoir triangulated with medical records | NHS Reference Costs and PSSRU |
| Community care | Specified within CRFs | CRFs at 4m and 12m with aide memoir triangulated with medical records | PSSRU and NHS Reference Costs |
| Medication (limited to)  *· PAH specific medications (pulmonary vasodilators etc)*  *· Oxygen therapy*  *· Iron supplementation for anaemia*  *· Diuretic*  *· Oral anticoagulants*  *· Calcium channel blockers* | Specified within concurrent medication form | Concurrent medication form | Prescription cost analysis [23] and/or BNF costs |
| Personal social services | Specified within CRFs | CRFs at 4m and 12m with aide memoir | PSSRU unit costs |

### 7.4.3 Training costs

To deliver the intervention successfully, it is necessary to train clinicians to ensure that both the supervised exercise program and the psychological intervention are delivered as intended. This requires time for both the trainers and the trainee. These training sessions will be recorded by the trial team.

Table 3: Training resource use

| **Resource Type** | **Resource use** | **How collected** | **Unit cost sources** |
| --- | --- | --- | --- |
| Supervised exercise training programme | Half a day of time for both the trainer and the practitioner | Recorded by trial team | PSSRU unit costs. Trainer and practitioner are likely to be Agenda for Change pay band 6. |
| Psychological intervention training | Half a day of time for both the trainer and the practitioner | Recorded by trial team | PSSRU unit costs |

### 7.4.4 Wider costs

Within an addition sensitivity analysis, we will also be collecting information related to days lost from work.

Table 4: Wider costs

| **Resource type** | **Resource use** | **How collected** | **Unit cost sources** |
| --- | --- | --- | --- |
| Absence from work | Specified within CRFs | CRFs at 4m and 12m | ONS salary data. |

## 7.5 Data integrity

On arrival in the trial office, data will be examined to ensure their integrity. Blinded descriptive data will be routinely reported and presented to the data monitoring committee (DMC), this includes proportion of missingness of the health economic variables. Any questionable data (e.g. outliers/high missingness) will be queried and followed up if necessary. The DMC will provide an opportunity to reflect whether the processes being used to collect data need refining or whether current methods are performing as desired. All data will be stored on secure University of Warwick servers in encrypted folders and access will be limited to only those approved to use it. Subsequently at the health economic analysis stage, variables will be range-checked and implausible vales queried.

## 7.6 Statistical analysis

### 7.6.1 Descriptive analysis

Resource use, costs and EQ-5D utility scores will first be presented descriptively to inform parameters for future health economic studies (this includes means and standard deviations). Costs will be calculated for all perspectives outlined previously. Additionally, differences between trial arms will be examined using standard statistical methods.

### 7.6.2 Addressing missing data with multiple imputation

If overall missing data for either costs or QALYs is more than 5% we will use multiple imputation to impute data within the base-case analysis. This data will then be used in the incremental analysis of costs, QALYs and the joint cost-effectiveness analysis. A complete case analysis will be included as a sensitivity analysis. Stata [24] will be used to conduct both the multiple imputation and the analysis of imputed data. The ‘mi impute chained’ command which uses chained equations to generate imputed datasets will be used for each treatment group. Within the imputation regression framework, we will include both costs and EQ-5D-5L at each timepoint as both imputed and predictor variables. We will also include any baseline variables that predict missingness [14] of costs or EQ-5D-5L. We will use predictive mean matching drawing from the 5 nearest ‘neighbours’, this is important for the avoidance of drawing implausible values, e.g. utility values over 1, and ‘negative costs’. The number of iterations will be guided by the fraction of missing information [12]. We will then use the ‘mi estimate’ functionality within Stata to run the analyses (specified in subsequent sections) within each dataset to combine imputation results using Rubin’s combination rule. We will examine the validity of the imputed data by comparing the distribution of the imputations and observed data both visually and statistically.

### 7.6.3 Cost-effectiveness analysis

It is anticipated that we will use bivariate regression analysis in the form of seemingly unrelated regressions (with bootstrapping) for the joint analysis of costs and QALYs. This framework offers several benefits: first of all it accounts for the existence of correlation between costs and outcomes for patients; second it allows the inclusion of covariates within the analysis, this is particularly relevant for the adjustment of baseline utility with respect to QALYs accrued; third it is generally robust to non-normal distributions; fourth, it can account for clustering either by including clusters as a fixed effect or by running linked regressions in a multi-level framework . Non-parametric bootstrapping will be used to examine the level of uncertainty by presenting the bootstrapped results on a cost-effectiveness plane. Should there be distributional or computational concerns then we may consider combining costs and outcomes within a univariate net-benefit regression framework.

#### 7.6.3.1 Characterizing uncertainty for decision makers

Intervention cost-effectiveness will be evaluated according to willingness to pay using the net-monetary benefit framework and bootstrapped regression model findings:

$$\Delta NB=\Delta e\gamma-\Delta c$$

Δ*NB* refers to the incremental net monetary benefit, Δ*e* reflects the incremental outcome of interest, incremental QALYs, whilst Δ*c* refers to the incremental costs. The symbol $\gamma$ refers to the decision maker’s willingness to pay per QALY. Using bootstrapped samples we will calculate the net-monetary benefit across a range of levels of willingness to pay ($\gamma)$. For each $\gamma$ the proportion of iterations where net-benefit is greater than zero can be used estimate the probability that the intervention is more cost-effective at that willingness to pay. Values of $\gamma$ will include £20,000 and £30,000 per QALY as specified by NICE and plotted to derive a CEAC [9].

#### 7.6.3.2 Sensitivity analyses

Planned sensitivity analyses will include:

- Costing from a societal perspective
- Complete case analysis (assuming missing data exceeds 5%).
- Trial randomisation strata as specified within the statistical analysis plan
- Clustering by centre

### 7.6.4 Value of information analysis

We will also conduct a value of information (VoI) analysis to examine the expected value of future research. The VoI analysis will entail the calculation of the expected value of perfect information (EVPI) using data from the cost-effectiveness analysis. EVPI can be conceptualised as the expected gain from eliminating uncertainty within the decision problem, or put another way, the expected loss associated with uncertainty. This is essentially the probability of the decision being wrong multiplied by the average consequence of being wrong [26]. This allows us to calculate the estimated value of ‘perfect knowledge’ which is the maximum value society should be willing to pay for additional evidence to reduce uncertainty around whether the intervention or the control is more cost-effective [27]. Using the trial data, we will calculate the per person EVPI using a willingness to pay threshold of £30,000 per QALY, representing the threshold NICE used in practice (Claxton et al 2015). This will be multiplied by the number of potential beneficiaries of the intervention within the NHS within the technological horizon (years) to estimate population EVPI. Discounting of EVPI will be applied at 3.5% beyond the first year.

### 7.6.5 Decision modelling

The primary trial-based analysis will compare the costs and QALYs of intervention and control accrued during the trial period. There is potential for (differences in) costs and benefits to accrue beyond the trial period. If outcomes have not converged by the 12m timepoint we will consider extrapolating the results over a longer time horizon using a decision analytic model. This would involve combining the trial data with external sources to estimate the long-term cost-effectiveness of the intervention. Any costs and benefits accruing after the first year would be discounted at a rate of 3.5% per year and full probabilistic sensitivity analysis would be conducted in line with the NICE reference case [9]. A decision as to the necessity of building a decision analytic model and its specification will be made following discussion between the health economists and the trial team following preliminary analysis of the data. This will be informed by things such as the conclusivity and direction of within trial results. For example, if the control dominates the intervention and extrapolation would only increase the strength of this results then extrapolating further is unwarranted.

# SECTION 8: Reference (Statistical Analysis Plan)

1. Kiely, D.G., et al., *Pulmonary hypertension: diagnosis and management.* BMJ, 2013. **346**: p. f2028.

2. Simonneau, G., et al., *Updated clinical classification of pulmonary hypertension.* J Am Coll Cardiol, 2013. **62**(25 Suppl): p. D34-41.

3. Vizza, C.D., et al., *Sildenafil dosed concomitantly with bosentan for adult pulmonary arterial hypertension in a randomized controlled trial.* BMC Cardiovasc Disord, 2017. **17**(1): p. 239.

4. Kim, N.H., *Group 4 Pulmonary Hypertension: Chronic Thromboembolic Pulmonary Hypertension: Epidemiology, Pathophysiology, and Treatment.* Cardiol Clin, 2016. **34**(3): p. 435-41.

5. Thenappan, T., et al., *Pulmonary arterial hypertension: pathogenesis and clinical management.* BMJ, 2018. **360**: p. j5492.

6. Fein, D.G., A.N. Zaidi, and R. Sulica, *Pulmonary Hypertension Due to Common Respiratory Conditions: Classification, Evaluation and Management Strategies.* J Clin Med, 2016. **5**(9).

7. Billings, C.G., et al., *Incremental shuttle walk test distance and autonomic dysfunction predict survival in pulmonary arterial hypertension.* J Heart Lung Transplant, 2017. **36**(8): p. 871-879.

8. McKenna, S.P., et al., *The Cambridge Pulmonary Hypertension Outcome Review (CAMPHOR): a measure of health-related quality of life and quality of life for patients with pulmonary hypertension.* Qual Life Res, 2006. **15**(1): p. 103-15.

9. Somaini, G., et al., *Prevalence of Anxiety and Depression in Pulmonary Hypertension and Changes during Therapy.* Respiration, 2016. **91**(5): p. 359-66.

10*.* Schwarzer R, Jerusalem M. Generalized self-efficacy scale. J. Weinman, S. Wright, & M. Johnston*, Measures in health psychology: A user’s portfolio. Causal and control beliefs. 1995;35:37.*

11. Krupp, L.B., et al., *The fatigue severity scale. Application to patients with multiple sclerosis and systemic lupus erythematosus.* Arch Neurol, 1989. **46**(10): p. 1121-3.

12. Yorke, J., et al., *Symptom severity and its effect on health-related quality of life over time in patients with pulmonary hypertension: a multisite longitudinal cohort study.* BMJ Open Respir Res, 2018. **5**(1): p. e000263.

13. McLaughlin, V.V. and M.D. McGoon, *Pulmonary arterial hypertension.* Circulation, 2006. **114**(13): p. 1417-31.

14. Frost, A.E., et al., *Evaluation of the predictive value of a clinical worsening definition using 2-year outcomes in patients with pulmonary arterial hypertension: a REVEAL Registry analysis.* Chest, 2013. **144**(5): p. 1521-1529.

15. Herdman, M., et al., *Development and preliminary testing of the new five-level version of EQ-5D (EQ-5D-5L).* Qual Life Res, 2011. **20**(10): p. 1727-36.

16. Moerbeek, M. and W.K. Wong, *Sample size formulae for trials comparing group and individual treatments in a multilevel model.* Stat Med, 2008. **27**(15): p. 2850-64.

17. Roberts C, Roberts SA. *Design and analysis of clinical trials with clustering effects due to treatment. Clinical trials. 2005 Apr;2(2):152-62.*

18. Connell AM. *Employing complier average causal effect analytic methods to examine effects of randomized encouragement trials. The American journal of drug and alcohol abuse.* 2009 Jan 1;35(4):253-9.

19. Mengersen, K.L., et al., *Bayesian Estimation of Small Effects in Exercise and Sports Science.* PLoS One, 2016. **11**(4): p. e0147311.

# SECTION 9: Reference (Health Economics Analysis Plan)

[1] D. G. Kiely, C. A. Elliot, I. Sabroe, and R. Condliffe, ‘Pulmonary hypertension: diagnosis and management’, *BMJ*, vol. 346, Apr. 2013, doi: 10.1136/bmj.f2028.

[2] D. L. Tran, E. M. T. Lau, D. S. Celermajer, G. M. Davis, and R. Cordina, ‘Pathophysiology of exercise intolerance in pulmonary arterial hypertension’, *Respirology*, vol. 23, no. 2, pp. 148–159, 2018, doi: 10.1111/resp.13141.

[3] N. Galiè *et al.*, ‘2015 ESC/ERS Guidelines for the diagnosis and treatment of pulmonary hypertensionThe Joint Task Force for the Diagnosis and Treatment of Pulmonary Hypertension of the European Society of Cardiology (ESC) and the European Respiratory Society (ERS): Endorsed by: Association for European Paediatric and Congenital Cardiology (AEPC), International Society for Heart and Lung Transplantation (ISHLT)’, *Eur. Heart J.*, vol. 37, no. 1, pp. 67–119, Jan. 2016, doi: 10.1093/eurheartj/ehv317.

[4] A. S. Babu, R. Arena, and N. R. Morris, ‘Evidence on Exercise Training in Pulmonary Hypertension’, in *Exercise for Cardiovascular Disease Prevention and Treatment: From Molecular to Clinical, Part 2*, J. Xiao, Ed. Singapore: Springer, 2017, pp. 153–172.

[5] T. Thenappan, M. L. Ormiston, J. J. Ryan, and S. L. Archer, ‘Pulmonary arterial hypertension: pathogenesis and clinical management’, *BMJ*, vol. 360, Mar. 2018, doi: 10.1136/bmj.j5492.

[6] D. G. Fein, A. N. Zaidi, and R. Sulica, ‘Pulmonary Hypertension Due to Common Respiratory Conditions: Classification, Evaluation and Management Strategies’, *J. Clin. Med.*, vol. 5, no. 9, Aug. 2016, doi: 10.3390/jcm5090075.

[7] G. McGregor *et al.*, ‘Supervised pulmonary hypertension exercise rehabilitation (SPHERe): study protocol for a multi-centre randomised controlled trial’, *BMC Pulm. Med.*, vol. 20, no. 1, p. 143, May 2020, doi: 10.1186/s12890-020-01182-y.

[8] ‘Keep active - exercise and PR | British Lung Foundation’. https://shop.blf.org.uk/products/pulmonary-rehabilitation-and-exercise (accessed Apr. 29, 2020).

[9] NICE, *Guide to the methods of technology appraisal 2013*. NICE, 2013.

[10] S. Ramsey *et al.*, ‘Cost-Effectiveness Analysis Alongside Clinical Trials II - An ISPOR Good Research Practives Task Force Report’, *Value Health*, vol. 18, pp. 161–172, 2015, doi: 10.1016/j.jval.2015.02.001.

[11] M. Gomes, K. Díaz-Ordaz, R. Grieve, and M. G. Kenward, ‘Multiple imputation methods for handling missing data in cost-effectiveness analyses that use data from hierarchical studies: an application to cluster randomized trials.’, *Med. Decis. Mak. Int. J. Soc. Med. Decis. Mak.*, vol. 33, no. 8, pp. 1051–63, Nov. 2013, doi: 10.1177/0272989X13492203.

[12] P. Madley-Dowd, R. Hughes, K. Tilling, and J. Heron, ‘The proportion of missing data should not be used to guide decisions on multiple imputation’, *J. Clin. Epidemiol.*, vol. 110, pp. 63–73, Jun. 2019, doi: 10.1016/j.jclinepi.2019.02.016.

[13] J. L. Schafer, ‘Multiple imputation: a primer’:, *Stat. Methods Med. Res.*, Jul. 2016, doi: 10.1177/096228029900800102.

[14] UCLA IDRE, ‘Multiple Imputation in Stata’. https://stats.idre.ucla.edu/stata/seminars/mi_in_stata_pt1_new/ (accessed May 14, 2020).

[15] J. A. C. Sterne *et al.*, ‘Multiple imputation for missing data in epidemiological and clinical research: potential and pitfalls.’, *BMJ*, vol. 338, no. jun29_1, p. b2393, Jan. 2009, doi: 10.1136/bmj.b2393.

[16] M. Herdman *et al.*, ‘Development and preliminary testing of the new five-level version of EQ-5D (EQ-5D-5L)’, *Qual. Life Res.*, vol. 20, no. 10, pp. 1727–1736, Dec. 2011, doi: 10.1007/s11136-011-9903-x.

[17] N. Devlin, K. Shah, Y. Feng, B. Mulhern, and B. van Hout, ‘Valuing health-related quality of life : An EQ-5D-5L value set for England’, *OHE Res. Pap.*, vol. January, no. January, 2016, [Online]. Available: https://www.ohe.org/publications/valuing-health-related-quality-life-eq-5d-5l-value-set-england.

[18] Hernández Alava, M., Pudney, S. & Wailoo, A. Estimating the Relationship Between EQ-5D-5L and EQ-5D-3L: Results from a UK Population Study. PharmacoEconomics 41, 199–207 (2023). https://doi.org/10.1007/s40273-022-01218- [19] C. Sampson, ‘Bad reasons not to use the EQ-5D-5L’, *The Academic Health Economists’ Blog*, Mar. 23, 2018. https://aheblog.com/2018/03/23/bad-reasons-not-to-use-the-eq-5d-5l/ (accessed Apr. 29, 2020).

[19] NICE position statement on EQ-5D-5L. [Position statement on use of the EQ-5D-5L value set for England (updated October 2019) | Technology appraisal guidance | NICE guidance | Our programmes | What we do | About | NICE](https://www.nice.org.uk/about/what-we-do/our-programmes/nice-guidance/technology-appraisal-guidance/eq-5d-5l) (accessed May 15 2023).

[20] NICE health technology evaluations: the manual. Process and methods [PMG36]Published: 31 January 2022

[21] A. Manca, N. Hawkins, and M. J. Sculpher, ‘Estimating mean QALYs in trial-based cost-effectiveness analysis: the importance of controlling for baseline utility.’, *Health Econ.*, vol. 14, no. 5, pp. 487–96, May 2005, doi: 10.1002/hec.944.

[22] ‘HRG4+ 2020/21 Local Payment Grouper’, *NHS Digital*. https://digital.nhs.uk/services/national-casemix-office/downloads-groupers-and-tools/local-payment-2020-21 (accessed May 14, 2020).

[23] ‘Prescription Cost Analysis’, *NHS Digital*. https://digital.nhs.uk/data-and-information/publications/statistical/prescription-cost-analysis (accessed May 14, 2020).

[24] StataCorp, *Stata statistical software: Release 16*. College Station, TX: StataCorp LLC, 2019.

[25] E. Fenwick, D. A. Marshall, A. R. Levy, and G. Nichol, ‘Using and interpreting cost-effectiveness acceptability curves: an example using data from a trial of management strategies for atrial fibrillation.’, *BMC Health Serv. Res.*, vol. 6, no. 1, p. 52, Jan. 2006, doi: 10.1186/1472-6963-6-52.

[26] E. C. F. Wilson, ‘A Practical Guide to Value of Information Analysis’, *PharmacoEconomics*, vol. 33, no. 2, pp. 105–121, Feb. 2015, doi: 10.1007/s40273-014-0219-x.

[27] H. V. Eeren, S. J. Schawo, R. H. J. Scholte, J. J. V. Busschbach, and L. Hakkaart, ‘Value of Information Analysis Applied to the Economic Evaluation of Interventions Aimed at Reducing Juvenile Delinquency: An Illustration’, *PLOS ONE*, vol. 10, no. 7, p. e0131255, Jul. 2015, doi: 10.1371/journal.pone.0131255.

# SECTION 10: Template Tables (Statistical Analysis Plan)

CONSORT diagram and dummy tables for statistical analysis

**Figure 1: CONSORT diagram for the SPHERe trial**


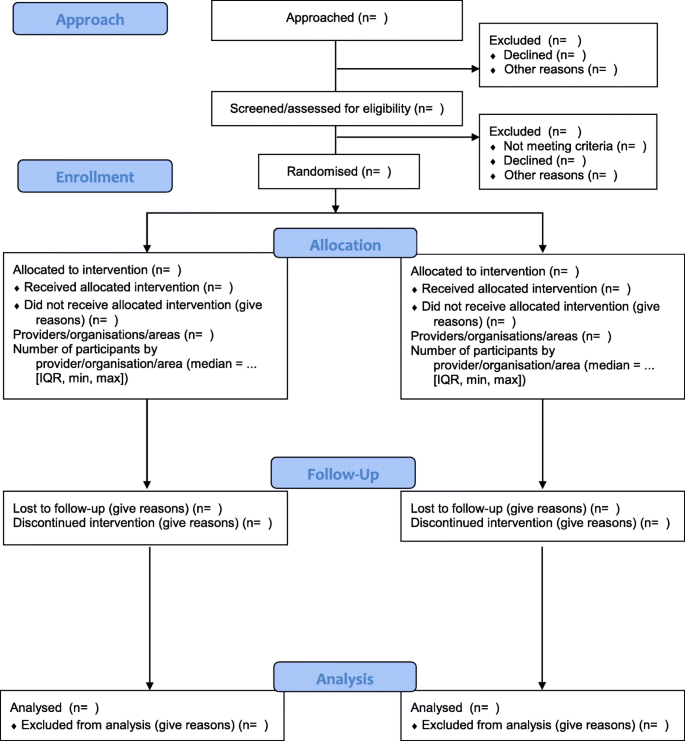


**Table 1: Screening of potential participants summarised by source – start with GP which is our main route of recruitment**

| **Source** | **Population** | **Number people with PH identified by search** | **Total Number Excluded** | **Total number invited** | **Total Number Replied** | **Total Number Consent to Approach** | **Total Number NO Consent to Approach** | **Total Number eligible** | **Total Number Ineligible** | **Total Number consented & randomised** |
| --- | --- | --- | --- | --- | --- | --- | --- | --- | --- | --- |
|  |  |  |  |  |  |  |  |  |  |  |
|  |  |  |  |  |  |  |  |  |  |  |
|  |  |  |  |  |  |  |  |  |  |  |
|  |  |  |  |  |  |  |  |  |  |  |
|  |  |  |  |  |  |  |  |  |  |  |
| **Total:** |  |  |  |  |  |  |  |  |  |  |

**Table 2: Randomised participants by treatment and randomisation strata**

|  | **Usual care** | **SPHERe intervention** | **Total** |
| --- | --- | --- | --- |
| **WHO Class** |  |  |  |
| **WHO Class II** | n (%) | n (%) | n (%) |
| **WHO Class III & IV** | n (%) | n (%) | n (%) |
| **PH Group** |  |  |  |
| **PH Group 1** | n (%) | n (%) | n (%) |
| **PH Group 2**  **PH Group 3**  **PH Group 4**  **PH Group 5** | n (%) | n (%) | n (%) |
|  | n (%) | n (%) | n (%) |
|  | n (%) | n (%) | n (%) |
|  | n (%) | n (%) | n (%) |
| **Centre/site** |  |  |  |
| **Site 1** | n (%) | n (%) | n (%) |
| **Site 2** | n (%) | n (%) | n (%) |
| **Site 3** | n (%) | n (%) | n (%) |
| **…………..** | n (%) | n (%) | n (%) |
| **Total** | N (%) | N (%) | N (%) |

**PARTICIPANT BASELINE AND DEMOGRAPHIC DATA**

**Table 3: Baseline demographics**

|  | **Usual care** | **SPHERe Intervention** | **TOTAL** |
| --- | --- | --- | --- |
| **Gender**  Male  Female  Other  Prefer not to say  Missing |  |  |  |
|  | n (%) | n (%) | n (%) |
|  | n (%) | n (%) | n (%) |
|  | n (%) | n (%) | n (%) |
|  | n (%) | n (%) | n (%) |
|  | n (%) | n (%) | n (%) |
| Age (years) |  |  |  |
| Mean (SD)  Median (IQR)  Range  Missing | xx (xx.x) | xx (xx.x) | xx (xx.x) |
|  | xx.x (xx,xx) | xx.x (xx,xx) | xx.x (xx,xx) |
|  | min, max | min, max | min, max |
|  | n (%) | n (%) | n (%) |
| **Ethnicity** |  |  |  |
| White | n (%) | n (%) | n (%) |
| Black Caribbean | n (%) | n (%) | n (%) |
| Black African | n (%) | n (%) | n (%) |
| Black Other | n (%) | n (%) | n (%) |
| Indian | n (%) | n (%) | n (%) |
| Pakistani | n (%) | n (%) | n (%) |
| Bangladeshi | n (%) | n (%) | n (%) |
| Chinese | n (%) | n (%) | n (%) |
| Prefer not to say | n (%) | n (%) | n (%) |
| Other | n (%) | n (%) | n (%) |
| Missing | n (%) | n (%) | n (%) |
| **In regular work**  Yes  No  Missing |  |  |  |
|  | n (%) | n (%) | n (%) |
|  | n (%) | n (%) | n (%) |
|  | n (%) | n (%) | n (%) |
| **What age left full time education** |  |  |  |
| No formal education | n (%) | n (%) | n (%) |
| Age 12 or less | n (%) | n (%) | n (%) |
| Age 13 to 16 | n (%) | n (%) | n (%) |
| Age 17 to 19  Age 20 or over  Still in education  Other  Missing | n (%) | n (%) | n (%) |
|  | n (%) | n (%) | n (%) |
|  | n (%) | n (%) | n (%) |
|  | n (%) | n (%) | n (%) |
|  | n (%) | n (%) | n (%) |
| **Do you live alone** |  |  |  |
| Yes | n (%) | n (%) | n (%) |
| No | n (%) | n (%) | n (%) |
| Missing | n (%) | n (%) | n (%) |

Note: All percentages are based on randomised patients.

**Table 4: Average age by gender for randomised participants**

| Age (years) by gender | **Usual care** | **SPHERe Intervention** | **TOTAL** |
| --- | --- | --- | --- |
| Male |  |  |  |
| N | n | n | n |
| Mean (SD) | xx (xx.x) | xx (xx.x) | xx (xx.x) |
| Range | min, max | min, max | min, max |
| Missing | n (%) | n (%) | n (%) |
| Female |  |  |  |
| N | n | n | n |
| Mean (SD) | xx (xx.x) | xx (xx.x) | xx (xx.x) |
| Range | min, max | min, max | min, max |
| Missing | n (%) | n (%) | n (%) |

**Table 5: Baseline outcomes for randomised participants**

|  | **Usual care** | **SPHERe Intervention** | **TOTAL** |
| --- | --- | --- | --- |
| **Incremental Shuttle Walk Test (metres)*** |  |  |  |
| N  Mean (SD)  Median (IQR)  Range  Missing | n | n | n |
|  | xx (xx.x) | xx (xx.x) | xx (xx.x) |
|  | xx.x (xx,xx) | xx.x (xx,xx) | xx.x (xx,xx) |
|  | min, max | min, max | min, max |
|  | n (%) | n (%) | n (%) |
| **WHO Functional Class** |  |  |  |
| Class II | n (%) | n (%) | n (%) |
| Class III & IV | n (%) | n (%) | n (%) |
| Missing | n (%) | n (%) | n (%) |
| **Adverse Events since consent**  Yes  No  Missing |  |  |  |
|  | n (%) | n (%) | n (%) |
|  | n (%) | n (%) | n (%) |
|  | n (%) | n (%) | n (%) |
| **Taking PH related medication** |  |  |  |
| Yes | n (%) | n (%) | n (%) |
| No  Missing | n (%) | n (%) | n (%) |
|  | n (%) | n (%) | n (%) |
| **CAMPHOR – Symptoms score**  Mean (SD)  Median (IQR)  Range  Missing |  |  |  |
|  | xx (xx.x) | xx (xx.x) | xx (xx.x) |
|  | xx.x (xx,xx) | xx.x (xx,xx) | xx.x (xx,xx) |
|  | min, max | min, max | min, max |
|  | n (%) | n (%) | n (%) |
| **CAMPHOR – Activity score**  Mean (SD) |  |  |  |
|  | xx (xx.x) | xx (xx.x) | xx (xx.x) |
| Median (IQR) | xx.x (xx,xx) | xx.x (xx,xx) | xx.x (xx,xx) |
| Range  Missing | min, max | min, max | min, max |
|  | n (%) | n (%) | n (%) |
| **CAMPHOR – Quality of life (QoL) score** |  |  |  |
| Mean (SD) | xx (xx.x) | xx (xx.x) | xx (xx.x) |
| Median (IQR) | xx.x (xx,xx) | xx.x (xx,xx) | xx.x (xx,xx) |
| Range  Missing | min, max | min, max | min, max |
|  | n (%) | n (%) | n (%) |
| **HADS – Anxiety score** |  |  |  |
| Mean (SD) | xx (xx.x) | xx (xx.x) | xx (xx.x) |
| Median (IQR) | xx.x (xx,xx) | xx.x (xx,xx) | xx.x (xx,xx) |
| Range  Missing | min, max | min, max | min, max |
|  | n (%) | n (%) | n (%) |
| **HADS – Depression score** |  |  |  |
| Mean (SD) | xx (xx.x) | xx (xx.x) | xx (xx.x) |
| Median (IQR) | xx.x (xx,xx) | xx.x (xx,xx) | xx.x (xx,xx) |
| Range | min, max | min, max | min, max |
| Missing | n (%) | n (%) | n (%) |
| **General Self Efficacy score** |  |  |  |
| Mean (SD) | xx (xx.x) | xx (xx.x) | xx (xx.x) |
| Median (IQR) | xx.x (xx,xx) | xx.x (xx,xx) | xx.x (xx,xx) |
| Range | min, max | min, max | min, max |
| Missing | n (%) | n (%) | n (%) |
| **Fatigue Severity Score** |  |  |  |
| Mean (SD) | xx (xx.x) | xx (xx.x) | xx (xx.x) |
| Median (IQR) | xx.x (xx,xx) | xx.x (xx,xx) | xx.x (xx,xx) |
| Range | min, max | min, max | min, max |
| Missing | n (%) | n (%) | n (%) |
| **EQ-5D-5L Utility Score**  Mean (SD)  Median (IQR)  Range  Missing |  |  |  |
|  | xx (xx.x) | xx (xx.x) | xx (xx.x) |
|  | xx.x (xx,xx) | xx.x (xx,xx) | xx.x (xx,xx) |
|  | min, max | min, max | min, max |
|  | n (%) | n (%) | n (%) |
| **EQ-5D-5L VAS Score**  Mean (SD)  Median (IQR)  Range  Missing |  |  |  |
|  | xx (xx.x) | xx (xx.x) | xx (xx.x) |
|  | xx.x (xx,xx) | xx.x (xx,xx) | xx.x (xx,xx) |
|  | min, max | min, max | min, max |
|  | n (%) | n (%) | n (%) |

Note: * Primary outcome; All percentages are based on randomised patients.

**PARTICIPANT FOLLOW-UP**

**Table 6: Overall summary of withdrawals by treatment arm**

|  | **Usual care** | **SPHERe Intervention** | **TOTAL** |
| --- | --- | --- | --- |
| Withdrawn from trial completely (discontinue intervention, and withdraw from follow-up and routine data and data collection) | n (%) | n (%) | n (%) |
| Withdrawn from consent for study use of data |  |  |  |
| Discontinue intervention and withdraw from follow-up only | n (%) | n (%) | n (%) |
| Discontinuation from intervention only | n (%) | n (%) | n (%) |

**Table 7: Reasons for withdrawal from trial (all withdrawals)**

| **Reasons** | **Usual care** | **SPHERe Intervention** | **TOTAL** |
| --- | --- | --- | --- |
| Participant does not have time to take part/too burdensome | n (%) | n (%) | n (%) |
| Participant does not believe the study will benefit them | n (%) | n (%) | n (%) |
| Participant finds travelling to site too difficult/burdensome | n (%) | n (%) | n (%) |
| Participant had a preference for the opposite study arm | n (%) | n (%) | n (%) |
| Withdrawal was practitioner decision | n (%) | n (%) | n (%) |
| No reason given | n (%) | n (%) | n (%) |
| Other | n (%) | n (%) | n (%) |
| Missing | n (%) | n (%) | n (%) |

**Table 8: Reasons for discontinuation from intervention, but remained on follow-up**

| **Reasons** | **SPHERe Intervention** | **TOTAL** |
| --- | --- | --- |
| Participant does not have time to take part/too burdensome | n (%) | n (%) |
| Participant does not believe the study will benefit them | n (%) | n (%) |
| Participant had a preference for the opposite study arm | n (%) | n (%) |
| Withdrawal was practitioner decision | n (%) | n (%) |
| No reason given | n (%) | n (%) |
| Other | n (%) | n (%) |
| Missing | n (%) | n (%) |

**Table 9: Follow-up rates throughout the trial**

| **Treatment Group** | **Time-point** | **Total to reach time point** | **Not followed up** | | **Follow-up due** | **Follow-up outcome** | | **Total completed**** |
| --- | --- | --- | --- | --- | --- | --- | --- | --- |
|  |  |  | **Deceased** | **Withdrawn completely** |  | **Completed*** | **Non Responder** |  |
| **Usual Care** | **Baseline** | N | n (%) | n (%) | N | n (%) | n (%) | n (%) |
|  | **4 month** | N | n (%) | n (%) | N | n (%) | n (%) | n (%) |
|  | **12 month** | N | n (%) | n (%) | N | n (%) | n (%) | n (%) |
| **SPHERe Intervention** | **Baseline** | N | n (%) | n (%) | N | n (%) | n (%) | n (%) |
|  | **4 month** | N | n (%) | n (%) | N | n (%) | n (%) | n (%) |
|  | **12 month** | N | n (%) | n (%) | N | n (%) | n (%) | n (%) |

*% out of follow-up due

**% out of total randomised

**Table 10: Timing of complete withdrawals throughout the trial**

|  | **Usual care** | **SPHERe Intervention** | **TOTAL** |
| --- | --- | --- | --- |
| Post-randomisation to < 4-month follow-up | n (%) | n (%) | n |
| ……… |  |  |  |
|  |  |  |  |
|  |  |  |  |
| Overall | n (%) | n (%) | n |

**Table 11: Protocol deviations and violations**

| **TNO** | **Issue** | **Date aware** | **Date resolved/actions implemented** | **Deviation/ violation** |  |
| --- | --- | --- | --- | --- | --- |
|  |  |  |  |  |  |
|  |  |  |  |  |  |

**INTERVENTION DATA**

**Table 12: Summary of adherence to trial intervention**

|  | Usual care | SPHERe Intervention | Total |
| --- | --- | --- | --- |
| Completed (fully complied) | n (%) | n (%) | n (%) |
| Partially completed | n (%) | n (%) | n (%) |
| Not completed | n (%) | n (%) | n (%) |
| *Reason 1* | n (%) | n (%) | n (%) |
| *Reason 2* | n (%) | n (%) | n (%) |
| *…* | n (%) | n (%) | n (%) |

Note: Full adherence will be considered as attending at least 6 out of 8 (75%) exercise sessions and at least 4 out of 6 (67%) psychosocial/motivational support sessions. Partial adherence will be defined as completion of the initial assessment/familiarisation and at least half of the, expected group online home exercise sessions, guided home exercise, and psychosocial/motivational sessions.

**Table 13: Summary of active intervention attendance**

|  | Usual care | SPHERe Intervention |
| --- | --- | --- |
| **Time from randomisation to initial 1:1 consultation (days)** |  |  |
| N |  |  |
| Mean (SD) |  |  |
| Median (IQR) |  |  |
| Didn’t attend first one-to-one |  |  |
| Missing |  |  |
| **Time from randomisation to first live group session (days)**  N  Mean (SD)  Median (IQR)  Didn’t attend first live session  Missing |  |  |
|  |  |  |
|  |  |  |
|  |  |  |
|  |  |  |
|  |  |  |
| **Live exercise session attendance (sessions)** |  |  |
| N |  |  |
| Mean (SD) |  |  |
| Median (IQR) |  |  |
| Attended none |  |  |
| Attended 1-5 sessions |  |  |
| Attended 6+ sessions |  |  |
| Attended 8 sessions |  |  |
| Missing |  |  |
| **Home exercise training and bike programme** |  |  |
| N |  |  |
| Mean (SD) |  |  |
| Median (IQR) |  |  |
| Attended none |  |  |
| Missing |  |  |
| **Psychological support session attendance (sessions)** |  |  |
| N |  |  |
| Mean (SD) |  |  |
| Median (IQR) |  |  |
| Attended none |  |  |
| Attended 1-3 sessions |  |  |
| Attended at least 4 sessions |  |  |
| Attended all 6 sessions |  |  |
| Missing |  |  |
| **Group size at randomisation** |  |  |
| N |  |  |
| Mean (SD) |  |  |
| Median (IQR) |  |  |
| Missing |  |  |

**STUDY OUTCOME DATA**

**Table 14: Study outcomes at 4 months follow-up**

|  | **Usual care** | **SPHERe Intervention** | **TOTAL** | **Unadjusted estimate (95% CI); p-value** | **Adjusted estimate (95% CI)**; p-value** |
| --- | --- | --- | --- | --- | --- |
| **Incremental Shuttle Walk Test (metres)*** |  |  |  |  |  |
| N  Mean (SD)  Median (IQR)  Range  Missing | n | n | n |  |  |
|  | xx (xx.x) | xx (xx.x) | xx (xx.x) |  |  |
|  | xx.x (xx,xx) | xx.x (xx,xx) | xx.x (xx,xx) |  |  |
|  | min, max | min, max | min, max |  |  |
|  | n (%) | n (%) | n (%) |  |  |
| **WHO Functional Class** |  |  |  |  |  |
| Class II  Class III & IV  Missing | n (%) | n (%) | n (%) |  |  |
|  | n (%) | n (%) | n (%) |  |  |
|  | n (%) | n (%) | n (%) |  |  |
| **Adverse Events since baseline assessment**  Yes  No  Missing |  |  |  |  |  |
|  | n (%) | n (%) | n (%) |  |  |
|  | n (%) | n (%) | n (%) |  |  |
|  | n (%) | n (%) | n (%) |  |  |
| **Taking PH related medication?** |  |  |  |  |  |
| Yes | n (%) | n (%) | n (%) |  |  |
| No  Missing | n (%) | n (%) | n (%) |  |  |
|  | n (%) | n (%) | n (%) |  |  |
| **CAMPHOR – Symptoms score**  N  Mean (SD)  Median (IQR)  Range  Missing |  |  |  |  |  |
|  | n | n | n |  |  |
|  | xx (xx.x) | xx (xx.x) | xx (xx.x) |  |  |
|  | xx.x (xx,xx) | xx.x (xx,xx) | xx.x (xx,xx) |  |  |
|  | min, max | min, max | min, max |  |  |
|  | n (%) | n (%) | n (%) |  |  |
| **CAMPHOR – Activity score**  N |  |  |  |  |  |
|  | n | n | n |  |  |
| Mean (SD) | xx (xx.x) | xx (xx.x) | xx (xx.x) |  |  |
| Median (IQR) | xx.x (xx,xx) | xx.x (xx,xx) | xx.x (xx,xx) |  |  |
| Range  Missing | min, max | min, max | min, max |  |  |
|  | n (%) | n (%) | n (%) |  |  |
| **CAMPHOR – Quality of life (QoL) score** |  |  |  |  |  |
| N | n | n | n |  |  |
| Mean (SD) | xx (xx.x) | xx (xx.x) | xx (xx.x) |  |  |
| Median (IQR) | xx.x (xx,xx) | xx.x (xx,xx) | xx.x (xx,xx) |  |  |
| Range  Missing | min, max | min, max | min, max |  |  |
|  | n (%) | n (%) | n (%) |  |  |
| **HADS – Anxiety score** |  |  |  |  |  |
| N | n | n | n |  |  |
| Mean (SD) | xx (xx.x) | xx (xx.x) | xx (xx.x) |  |  |
| Median (IQR) | xx.x (xx,xx) | xx.x (xx,xx) | xx.x (xx,xx) |  |  |
| Range  Missing | min, max | min, max | min, max |  |  |
|  | n (%) | n (%) | n (%) |  |  |
| **HADS – Depression score** |  |  |  |  |  |
| N | n | n | n |  |  |
| Mean (SD) | xx (xx.x) | xx (xx.x) | xx (xx.x) |  |  |
| Median (IQR) | xx.x (xx,xx) | xx.x (xx,xx) | xx.x (xx,xx) |  |  |
| Range | min, max | min, max | min, max |  |  |
| Missing | n (%) | n (%) | n (%) |  |  |
| **General Self Efficacy score** |  |  |  |  |  |
| N | n | n | n |  |  |
| Mean (SD) | xx (xx.x) | xx (xx.x) | xx (xx.x) |  |  |
| Median (IQR) | xx.x (xx,xx) | xx.x (xx,xx) | xx.x (xx,xx) |  |  |
| Range | min, max | min, max | min, max |  |  |
| Missing | n (%) | n (%) | n (%) |  |  |
| **Fatigue Severity Score** |  |  |  |  |  |
| N | n | n | n |  |  |
| Mean (SD) | xx (xx.x) | xx (xx.x) | xx (xx.x) |  |  |
| Median (IQR) | xx.x (xx,xx) | xx.x (xx,xx) | xx.x (xx,xx) |  |  |
| Range | min, max | min, max | min, max |  |  |
| Missing | n (%) | n (%) | n (%) |  |  |
| **EQ-5D-5L Utility Score**  N  Mean (SD)  Median (IQR)  Range  Missing |  |  |  |  |  |
|  | n | n | n |  |  |
|  | xx (xx.x) | xx (xx.x) | xx (xx.x) |  |  |
|  | xx.x (xx,xx) | xx.x (xx,xx) | xx.x (xx,xx) |  |  |
|  | min, max | min, max | min, max |  |  |
|  | n (%) | n (%) | n (%) |  |  |
| **EQ-5D-5L VAS Score**  N  Mean (SD)  Median (IQR)  Range  Missing |  |  |  |  |  |
|  | n | n | n |  |  |
|  | xx (xx.x) | xx (xx.x) | xx (xx.x) |  |  |
|  | xx.x (xx,xx) | xx.x (xx,xx) | xx.x (xx,xx) |  |  |
|  | min, max | min, max | min, max |  |  |
|  | n (%) | n (%) | n (%) |  |  |

* Primary outcome ** Based on heteroscedastic linear regression model adjusted for WHO functional class, PH group, age, gender, centre, and duration of time in follow-up to the ISWT. The group effect was included as a random effect to account for partial clustering.

**Table 15: Study outcomes at 12 months follow-up**

|  | **Usual care** | **SPHERe Intervention** | **TOTAL** | **Unadjusted estimate (95% CI); p-value** | **Adjusted estimate (95% CI)**; p-value** |
| --- | --- | --- | --- | --- | --- |
| **Incremental Shuttle Walk Test (metres)*** |  |  |  |  |  |
| N  Mean (SD)  Median (IQR)  Range  Missing | n | n | n |  |  |
|  | xx (xx.x) | xx (xx.x) | xx (xx.x) |  |  |
|  | xx.x (xx,xx) | xx.x (xx,xx) | xx.x (xx,xx) |  |  |
|  | min, max | min, max | min, max |  |  |
|  | n (%) | n (%) | n (%) |  |  |
| **WHO Functional Class** |  |  |  |  |  |
| Class II | n (%) | n (%) | n (%) |  |  |
| Class III & IV | n (%) | n (%) | n (%) |  |  |
| Missing | n (%) | n (%) | n (%) |  |  |
| **Adverse Events since baseline assessment**  Yes  No  Missing |  |  |  |  |  |
|  | n (%) | n (%) | n (%) |  |  |
|  | n (%) | n (%) | n (%) |  |  |
|  | n (%) | n (%) | n (%) |  |  |
| **Taking PH related medication** |  |  |  |  |  |
| Yes | n (%) | n (%) | n (%) |  |  |
| No | n (%) | n (%) | n (%) |  |  |
| Missing | n (%) | n (%) | n (%) |  |  |
|  |  |  |  |  |  |
| **CAMPHOR – Symptoms score**  N  Mean (SD)  Median (IQR)  Range  Missing |  |  |  |  |  |
|  | n | n | n |  |  |
|  | xx (xx.x) | xx (xx.x) | xx (xx.x) |  |  |
|  | xx.x (xx,xx) | xx.x (xx,xx) | xx.x (xx,xx) |  |  |
|  | min, max | min, max | min, max |  |  |
|  | n (%) | n (%) | n (%) |  |  |
| **CAMPHOR – Activity score**  N |  |  |  |  |  |
|  | n | n | n |  |  |
| Mean (SD) | xx (xx.x) | xx (xx.x) | xx (xx.x) |  |  |
| Median (IQR) | xx.x (xx,xx) | xx.x (xx,xx) | xx.x (xx,xx) |  |  |
| Range  Missing | min, max | min, max | min, max |  |  |
|  | n (%) | n (%) | n (%) |  |  |
| **CAMPHOR – Quality of life (QoL) score** |  |  |  |  |  |
| N | n | n | n |  |  |
| Mean (SD) | xx (xx.x) | xx (xx.x) | xx (xx.x) |  |  |
| Median (IQR) | xx.x (xx,xx) | xx.x (xx,xx) | xx.x (xx,xx) |  |  |
| Range  Missing | min, max | min, max | min, max |  |  |
|  | n (%) | n (%) | n (%) |  |  |
| **HADS – Anxiety score** |  |  |  |  |  |
| N | n | n | n |  |  |
| Mean (SD) | xx (xx.x) | xx (xx.x) | xx (xx.x) |  |  |
| Median (IQR) | xx.x (xx,xx) | xx.x (xx,xx) | xx.x (xx,xx) |  |  |
| Range  Missing | min, max | min, max | min, max |  |  |
|  | n (%) | n (%) | n (%) |  |  |
| **HADS – Depression score** |  |  |  |  |  |
| N | n | n | n |  |  |
| Mean (SD) | xx (xx.x) | xx (xx.x) | xx (xx.x) |  |  |
| Median (IQR) | xx.x (xx,xx) | xx.x (xx,xx) | xx.x (xx,xx) |  |  |
| Range | min, max | min, max | min, max |  |  |
| Missing | n (%) | n (%) | n (%) |  |  |
| **General Self Efficacy score** |  |  |  |  |  |
| N | n | n | n |  |  |
| Mean (SD) | xx (xx.x) | xx (xx.x) | xx (xx.x) |  |  |
| Median (IQR) | xx.x (xx,xx) | xx.x (xx,xx) | xx.x (xx,xx) |  |  |
| Range | min, max | min, max | min, max |  |  |
| Missing | n (%) | n (%) | n (%) |  |  |
| **Fatigue Severity Score** |  |  |  |  |  |
| N | n | n | n |  |  |
| Mean (SD) | xx (xx.x) | xx (xx.x) | xx (xx.x) |  |  |
| Median (IQR) | xx.x (xx,xx) | xx.x (xx,xx) | xx.x (xx,xx) |  |  |
| Range | min, max | min, max | min, max |  |  |
| Missing | n (%) | n (%) | n (%) |  |  |
| **EQ-5D-5L Utility Score**  N  Mean (SD)  Median (IQR)  Range  Missing |  |  |  |  |  |
|  | n | n | n |  |  |
|  | xx (xx.x) | xx (xx.x) | xx (xx.x) |  |  |
|  | xx.x (xx,xx) | xx.x (xx,xx) | xx.x (xx,xx) |  |  |
|  | min, max | min, max | min, max |  |  |
|  | n (%) | n (%) | n (%) |  |  |
| **EQ-5D-5L VAS Score**  N  Mean (SD)  Median (IQR)  Range  Missing |  |  |  |  |  |
|  | n | n | n |  |  |
|  | xx (xx.x) | xx (xx.x) | xx (xx.x) |  |  |
|  | xx.x (xx,xx) | xx.x (xx,xx) | xx.x (xx,xx) |  |  |
|  | min, max | min, max | min, max |  |  |
|  | n (%) | n (%) | n (%) |  |  |

* Primary outcome ** Based on heteroscedastic linear regression model adjusted for WHO functional class, PH group, age, gender, centre, and duration of time in follow-up to the ISWT. The group effect was included as a random effect to account for partial clustering.

**Table 16: ITT and CACE model estimates of treatment difference at each time point (using full compliance)**

|  | **ITT model** | | **CACE model** | |
| --- | --- | --- | --- | --- |
|  | **Mean difference (95% CI)*** | **p-value** | **Mean difference (95% CI)^†^** | **p-value** |
| **Incremental Shuttle Walk Test (metres)** |  |  |  |  |
| 4 months | Xx (xx,xx) | xxx | Xx (xx,xx) | xxx |
| 12 months | Xx (xx,xx) | xxx | Xx (xx,xx) | xxx |

* Based on heteroscedastic linear regression model adjusted for WHO functional class, PH group, age, gender, centre, and duration of time in follow-up to the ISWT. The group effect was included as a random effect to account for partial clustering.

†Based on a single equation instrumental variable regression model with outcome adjusted for WHO functional class, PH group, age, gender, centre, and duration of time in follow-up to the ISWT. Full adherence will be considered as attending at least 6 out of 8 (75%) exercise sessions and at least 4 out of 6 (67%) psychosocial/motivational support sessions.

**Table 17: Sub-group analyses of the 4-month Incremental Shuttle Walk Test (metres) outcome**

| **Subgroups** | **Usual care**  **N; mean(95% CI)** | **SPHERe Intervention**  **N; mean(95% CI)** | **Interaction effect (95% CI); p-value*** |
| --- | --- | --- | --- |
|  |  |  |  |
| **WHO Class** |  |  | P=xxx |
| Class II | n; xx.x (xx, xx) | n; xx.x (xx, xx) |  |
| Class III & IV | n; xx.x (xx, xx) | n; xx.x (xx, xx) |  |
|  |  |  |  |
| **PH group**  Group 2&3  Group 1,4,&5 |  |  | P=xxx |
|  | n; xx.x (xx, xx) | n; xx.x (xx, xx) |  |
|  | n; xx.x (xx, xx) | n; xx.x (xx, xx) |  |
|  |  |  |  |

*Based on heteroscedastic linear regression model adjusted for WHO functional class, PH group, age, gender, centre, and duration of time in follow-up to the ISWT. The group effect was included as a random effect to account for partial clustering.

**Table 18: Treatment effectiveness estimate based on the primary outcomes at each time point having using imputed datasets.**

|  | **Usual care**  **N=** | **SPHERe Intervention**  **N=** | **Unadjusted estimate (95% CI); p-value** | **Adjusted estimate (95% CI); p-value*** |
| --- | --- | --- | --- | --- |
| **Incremental Shuttle Walk Test (metres)** |  |  | xx.x (x.x, xx); p= | xx.x (x.x, x.x); p= |
| N | n | n |  |  |
| Mean (SD) | xx.x (x.x) | xx.x (x.x) |  |  |
| Median (IQR) | xx.x (x.x, x.x) | xx.x (x.x, x.x) |  |  |
| Missing | xx | xx |  |  |

*** Based on heteroscedastic linear regression model adjusted for WHO functional class, PH group, age, gender, centre, and duration of time in follow-up to the ISWT. The group effect was included as a random effect to account for partial clustering.

**Table 19: ITT and CACE model estimates of treatment difference at each time point (using alternative definition of partial compliance)**

|  | **ITT model** | | **CACE model** | |
| --- | --- | --- | --- | --- |
|  | **Mean difference (95% CI)*** | **p-value** | **Mean difference (95% CI)^†^** | **p-value** |
| **Incremental Shuttle Walk Test (metres)** |  |  |  |  |
| 4 months | xx (xx, xx) | xxx | xx (xx, xx) | xxx |
|  |  |  |  |  |

*** Based on heteroscedastic linear regression model adjusted for WHO functional class, PH group, age, gender, centre, and duration of time in follow-up to the ISWT. The group effect was included as a random effect to account for partial clustering.

†Based on a single equation instrumental variable regression model with outcome adjusted for WHO functional class, PH group, age, gender, and duration of time in follow-up to the ISWT. Alternative partial adherence will be defined as completion of the initial assessment/familiarisation and at least half of the, expected group online home exercise sessions, guided home exercise, and psychosocial/motivational sessions.

**ADVERSE EVENTS AND SERIOUS ADVERSE EVENTS**

**Table 20: Adverse events (AE) and serious adverse events (SAE) summarised by treatment group***

|  | **Usual care**  **N=** | **SPHERe Intervention**  **N=** | **Total**  **N=** |
| --- | --- | --- | --- |
| **AEs** |  |  |  |
| Number of AEs reported | n (%) | n (%) | n (%) |
| **SAEs** |  |  |  |
| Number of SAEs reported | n (%) | n (%) | n (%) |
| **Reason Serious Adverse Event deemed serious** |  |  |  |
| Death | n (%) | n (%) | n (%) |
| Life-threatening | n (%) | n (%) | n (%) |
| Hospitalisation or prolongation of existing hospitalisation | n (%) | n (%) | n (%) |
| Persistent or significant disability or incapacity | n (%) | n (%) | n (%) |
| Congenital anomaly/birth defect | n (%) | n (%) | n (%) |
| Important medical condition | n (%) | n (%) | n (%) |
| Other | n (%) | n (%) | n (%) |
| **SAE severity assessment** |  |  |  |
| Mild | n (%) | n (%) | n (%) |
| Moderate | n (%) | n (%) | n (%) |
| Severe | n (%) | n (%) | n (%) |
| Fatal/life threatening | n (%) | n (%) | n (%) |

* % out of total randomised.

**Table 21: Assessment of SAEs summarised by treatment group***

| **Assessment of SAE’s** | **Usual care**  **N=** | **SPHERe Intervention**  **N=** | **TOTAL**  **N=** |
| --- | --- | --- | --- |
| **SAE related to trial intervention:** |  |  |  |
| Definitely | n (%) | n (%) | n (%) |
| Probably | n (%) | n (%) | n (%) |
| Possibly | n (%) | n (%) | n (%) |
| Unlikely | n (%) | n (%) | n (%) |
| Unrelated | n (%) | n (%) | n (%) |
| **Expectedness of SAE:** |  |  |  |
| Expected | n (%) | n (%) | n (%) |
| Unexpected | n (%) | n (%) | n (%) |

* % out of total randomised.

**Table 22: List of adverse events by treatment group**

| **TNO** | **Date** | **Details** |
| --- | --- | --- |
|  |  |  |
|  |  |  |
|  |  |  |

**Table 23: List of serious adverse events by treatment group**

| **TNO** | **Date** | **Type** | **Related** | **Details** |
| --- | --- | --- | --- | --- |
|  |  |  |  |  |
|  |  |  |  |  |
|  |  |  |  |  |

**Table 24: Allocation concealment by treatment group**

| **Allocation Concealment** | **Usual Care** | **SPHERe Intervention** | **Total** |
| --- | --- | --- | --- |
| **Yes** | n (%) | n (%) | n (%) |
| **No** | n (%) | n (%) | n (%) |
| **Missing** | n (%) | n (%) | n (%) |
| **If yes, which study arm**  SPHERe intervention  Usual care |  |  |  |
|  | n (%) | n (%) | n (%) |
|  | n (%) | n (%) | n (%) |
| **If yes, how made aware of the study arm**  Participant said  Other |  |  |  |
|  | n (%) | n (%) | n (%) |
|  | n (%) | n (%) | n (%) |

# SECTION 11: Template Tables (Health Economics Analysis Plan)

### Table 1: Completeness of data by follow-up visit

|  | **Control** | | **Intervention** | | **Total** | |
| --- | --- | --- | --- | --- | --- | --- |
|  | **n** | **(%, N)** | **n** | **(%, N)** | **n** | **(%, N)** |
| **Health status^1^** |  |  |  |  |  |  |
| EQ-5D Baseline |  |  |  |  |  |  |
| EQ-5D 4 months |  |  |  |  |  |  |
| EQ-5D 12 months |  |  |  |  |  |  |
| EQ-5D All visits |  |  |  |  |  |  |
| **Resource use^2^** |  |  |  |  |  |  |
| Inpatient |  |  |  |  |  |  |
| Outpatient |  |  |  |  |  |  |
| Community |  |  |  |  |  |  |
| Personal social services |  |  |  |  |  |  |
| Work absence |  |  |  |  |  |  |

1.EQ-5D-5L index score

2. Range shown (4M-12M)

### Table 2: Health Status, resource use and cost (complete cases)

|  | **Control** | | **Intervention** | | **Total** | |
| --- | --- | --- | --- | --- | --- | --- |
|  | **mean** | **(SD)** | **mean** | **(SD)** | **mean** | **(SD)** |
| **Health status^1^** |  |  |  |  |  |  |
| EQ-5D Baseline |  |  |  |  |  |  |
| EQ-5D 4 months |  |  |  |  |  |  |
| EQ-5D 12 months |  |  |  |  |  |  |
| QALYs |  |  |  |  |  |  |
| **Resource use** (all visits) |  |  |  |  |  |  |
| Inpatient days |  |  |  |  |  |  |
| Outpatient visits |  |  |  |  |  |  |
| A&E Visits |  |  |  |  |  |  |
| Community |  |  |  |  |  |  |
| GP surgery visits |  |  |  |  |  |  |
| GP home visits |  |  |  |  |  |  |
| GP telephone contacts |  |  |  |  |  |  |
| Practice nurse contacts |  |  |  |  |  |  |
| District nurse contacts |  |  |  |  |  |  |
| Community Physiotherapy contacts |  |  |  |  |  |  |
| Other physiotherapy contacts |  |  |  |  |  |  |
| NHS Direct contacts |  |  |  |  |  |  |
| Calls for ambulance/paramedic |  |  |  |  |  |  |
| Occupational therapy contacts |  |  |  |  |  |  |
| Other community contacts |  |  |  |  |  |  |
| Personal social services^2^ |  |  |  |  |  |  |
| Work absence (days)  Medications |  |  |  |  |  |  |
| **Cost**^3^ |  |  |  |  |  |  |
| A: Cost (study procedures) |  |  |  |  |  |  |
| B: Cost (NHS contacts) |  |  |  |  |  |  |
| C: Cost (Personal social services) |  |  |  |  |  |  |
| Cost (Total, A+B+C) |  |  |  |  |  |  |
|  |  |  |  |  |  |  |

1 EQ-5D-5L index score

2 Includes: meals on wheels, laundry services, social worker, care worker, home helper and other specified contacts

3 Time from work is not included in the analytic perspective, which includes health service and personal social services costs

### Table 3: Cost-effectiveness results

|  |  | **Incremental cost**  **(95%CI)** | **Incremental QALYs**  **(95%CI)** | **ICER**  **(95%CI)** | **p^1^** | **p^2^** | **NMB^1^** | **NMB^2^** |
| --- | --- | --- | --- | --- | --- | --- | --- | --- |
| Base case | |  |  |  |  |  |  |  |
|  | Imputed costs and QALYs, baseline EQ-5D adjusted |  |  |  |  |  |  |  |
|  |  |  |  |  |  |  |  |  |
| Sensitivity analyses | |  |  |  |  |  |  |  |
| 1 | Inclusion of societal costs |  |  |  |  |  |  |  |
|  |  |  |  |  |  |  |  |  |
| 2 | Complete case analysis |  |  |  |  |  |  |  |
|  |  |  |  |  |  |  |  |  |
| 3 | Base case: sub-group analyses specified in the SAP |  |  |  |  |  |  |  |
|  |  |  |  |  |  |  |  |  |
| **^1^** probability cost-effective or net monetary benefit if willing to pay £20,000/QALY. **^2^** probability cost-effective or net monetary benefit if willing to pay £30,000/QALY | | | | | | | | |
